# Supplementary material for: Tuning the Anthranilamide Peptidomimetic Design to Selectively Target Planktonic Bacteria and Biofilm
Source: Antibiotics (Basel). 2023 Mar 15;12(3):585. doi: 10.3390/antibiotics12030585 (PMC10044445; doi:10.3390/antibiotics12030585)

The Supplementary Data for

Tuning the anthranilamide peptidomimetic design to selectively target planktonic bacteria and biofilm

Rajesh Kuppusamy, Muhammad Yasir, Tsz Tin Yu, Florida Voli, Orazio Vittorio, Michael J. Miller, Peter Lewis, David StC Black, Mark Willcox and Naresh Kumar

The analytical data for compounds upto 6a and the intermediate **10a** and the final compound **12a** were published already [20]

^{1}H NMR of *tert*-butyl (S)-(2-(2-(2-amino-5-bromobenzamido)-3-(1*H*-indol-3-yl)propanamido)ethyl)carbamate (6a)

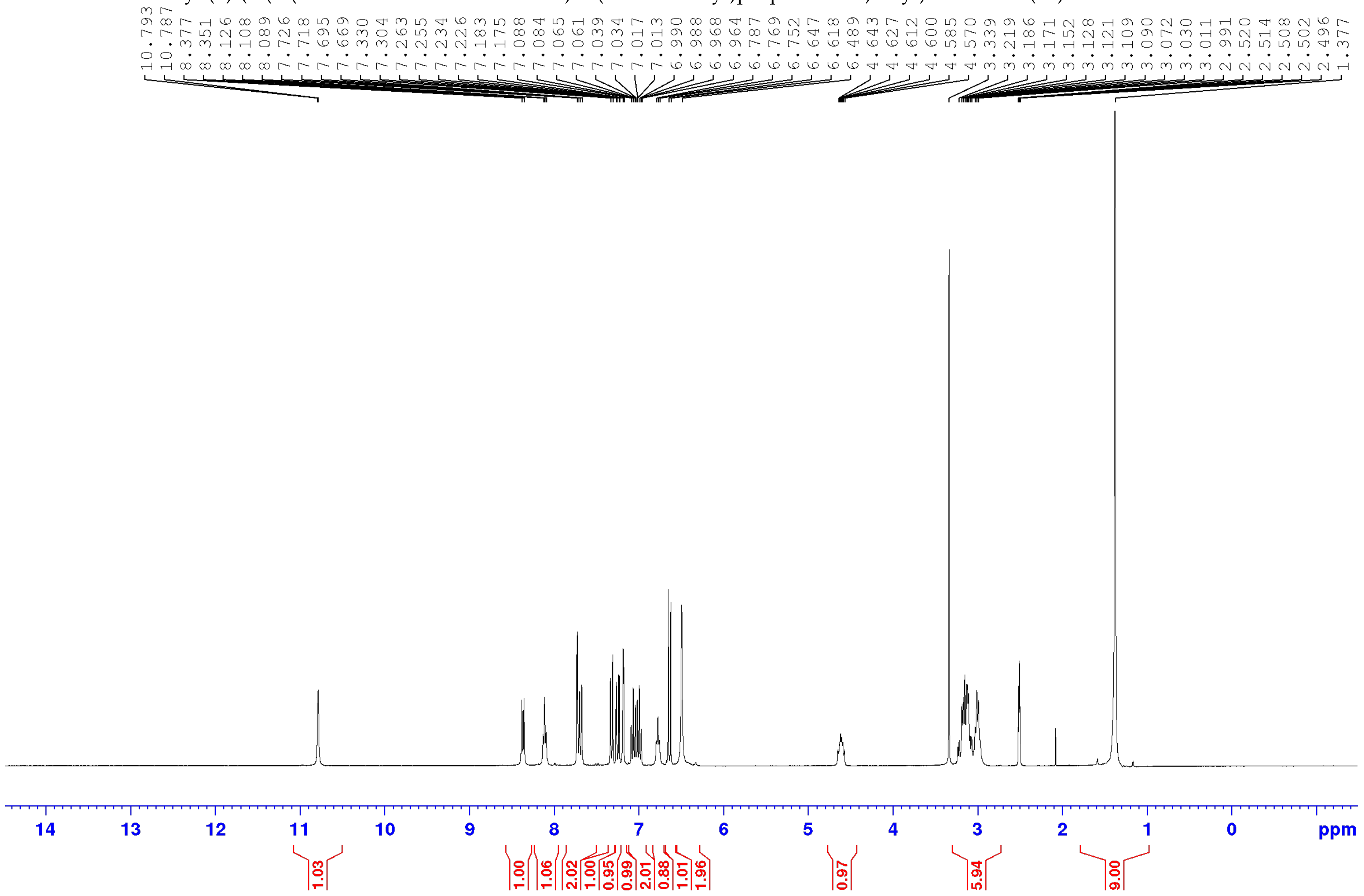

^{13}C NMR of *tert*-butyl (S)-(2-(2-(2-amino-5-bromobenzamido)-3-(1*H*-indol-3-yl)propanamido)ethyl)carbamate (6a)

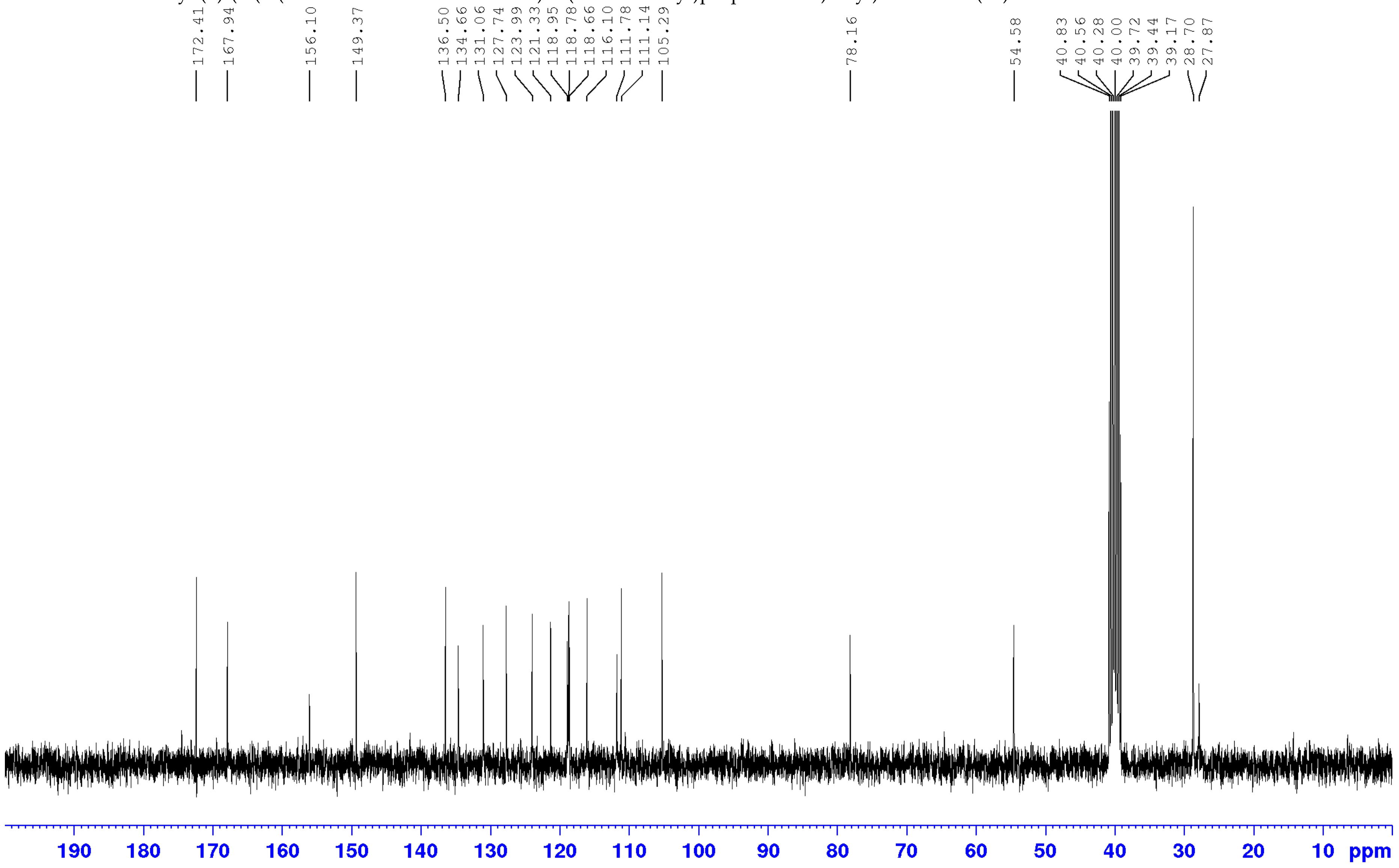

^{1}H NMR of *tert*-butyl (S)-(3-(2-(2-amino-5-bromobenzamido)-3-(1*H*-indol-3-yl)propanamido)propyl)carbamate (6b)

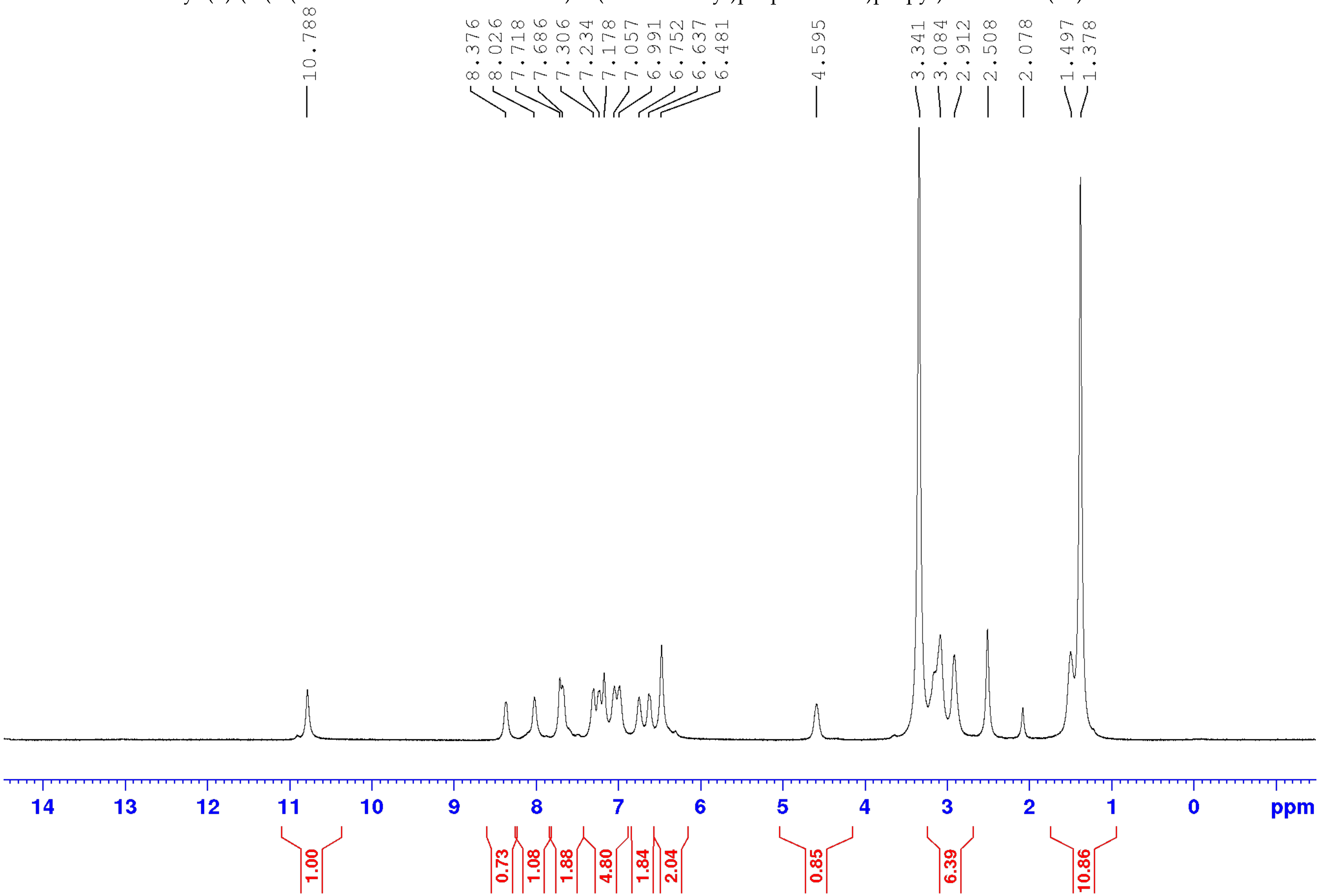

^{13}C NMR of *tert*-butyl (S)-(3-(2-(2-amino-5-bromobenzamido)-3-(1*H*-indol-3-yl)propanamido)propyl)carbamate (6b)

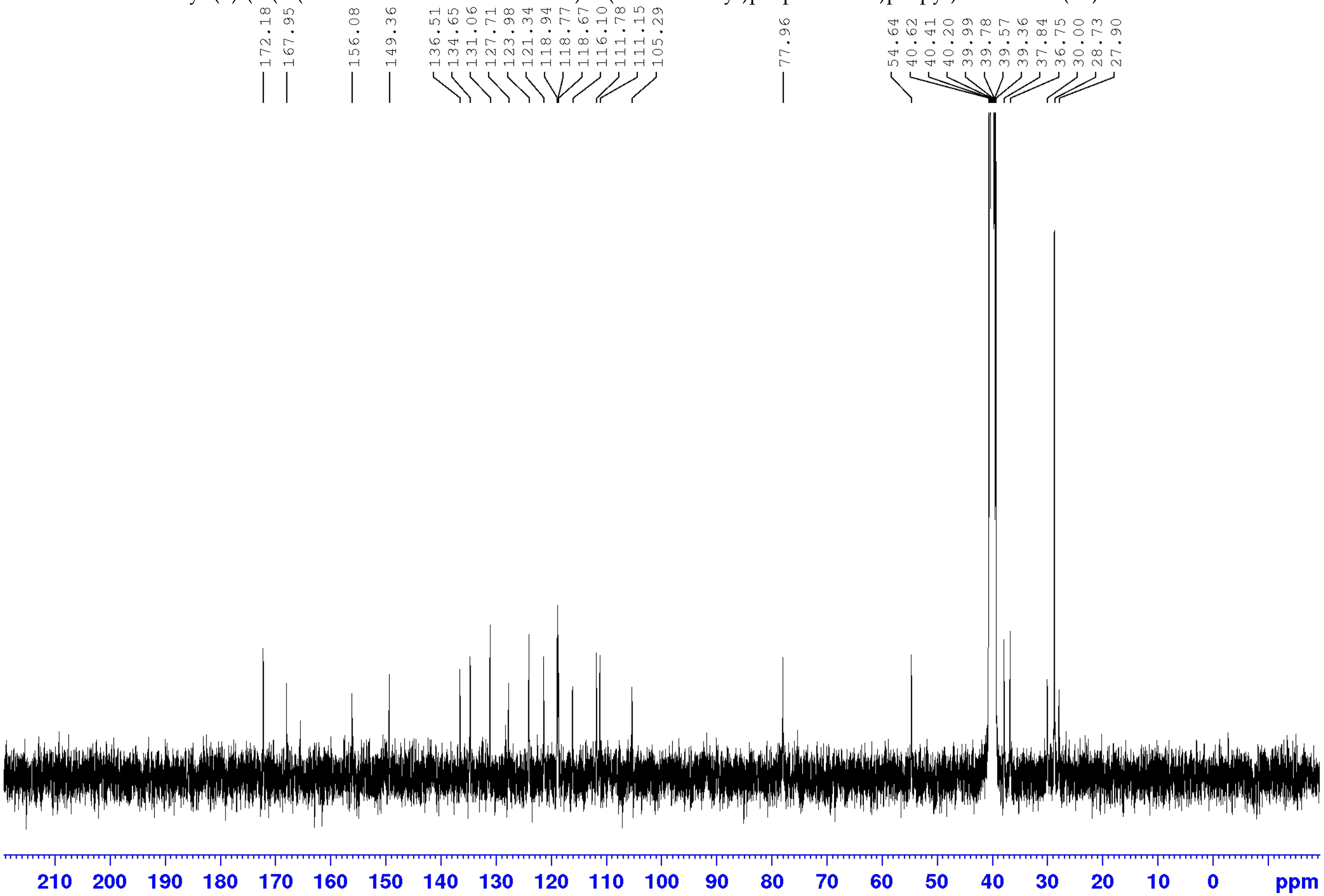

^{1}H NMR of *tert*-butyl (S)-(2-(2-(5-bromo-2-(2-(naphthalen-1-yl)acetamido)benzamido)-3-(1*H*-indol-3-yl)propanamido)ethyl)carbamate (10b)

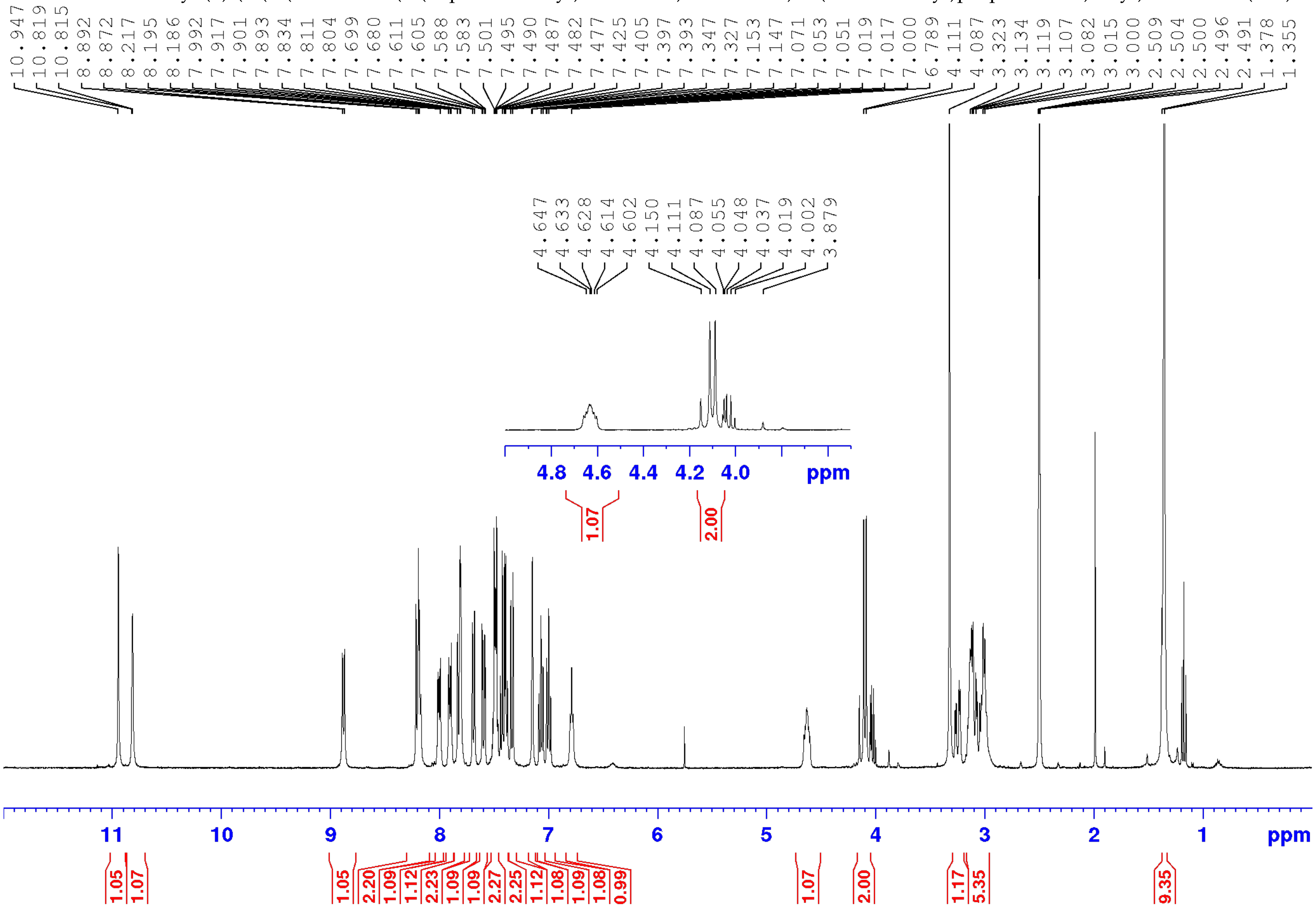

^{13}C NMR of *tert*-butyl (S)-(2-(2-(5-bromo-2-(2-(naphthalen-1-yl)acetamido)benzamido)-3-(1*H*-indol-3-yl)propanamido)ethyl)carbamate (10b)

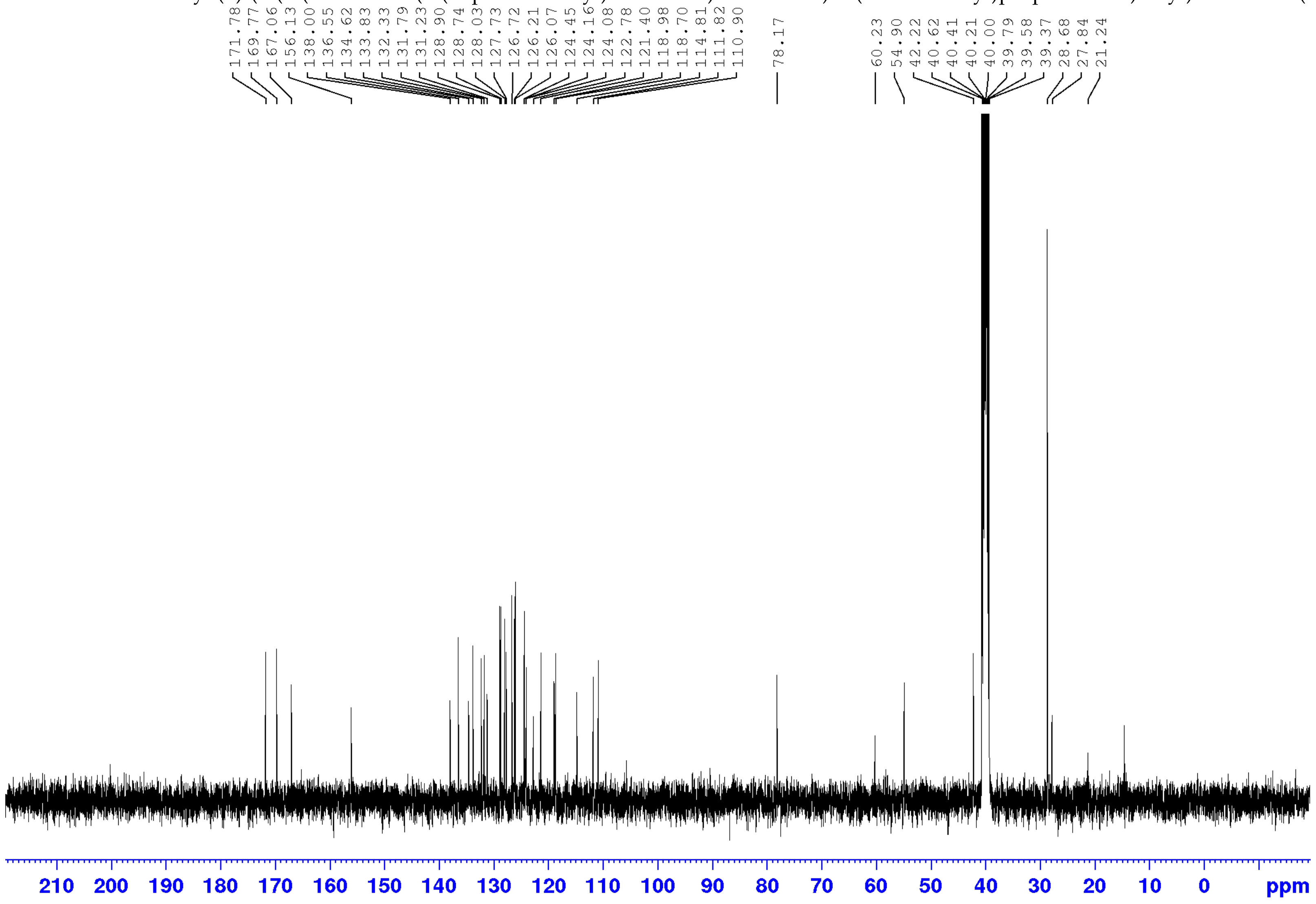

^{1}H NMR of *tert*-butyl (S)-(2-(2-(5-bromo-2-(2-methoxy-1-naphthamido)benzamido)-3-(1H-indol-3-yl)propanamido)ethyl)carbamate (10c)

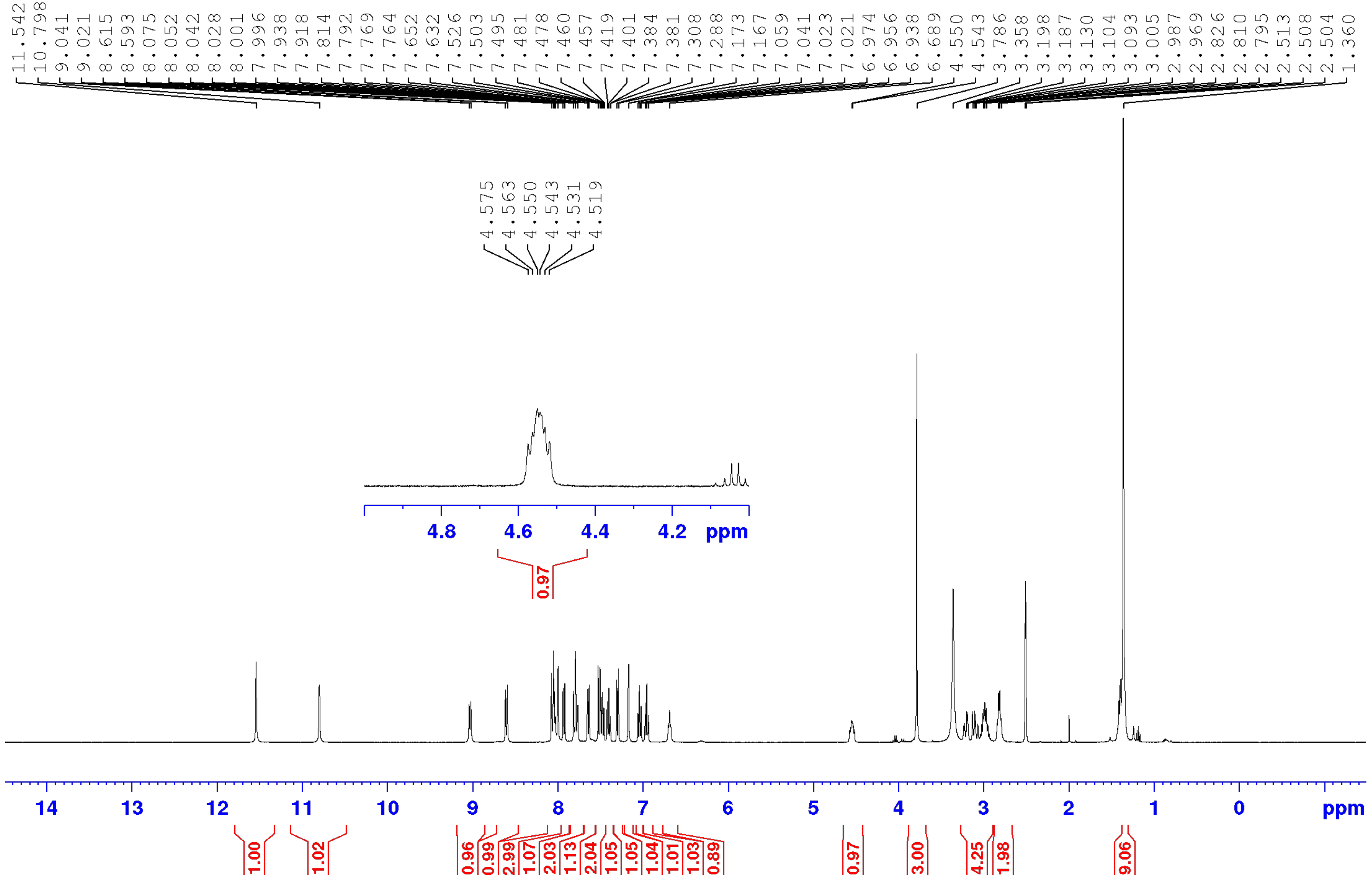

^{13}C NMR of *tert*-butyl (S)-(2-(2-(5-bromo-2-(2-methoxy-1-naphthamido)benzamido)-3-(1H-indol-3-yl)propanamido)ethyl)carbamate (10c)

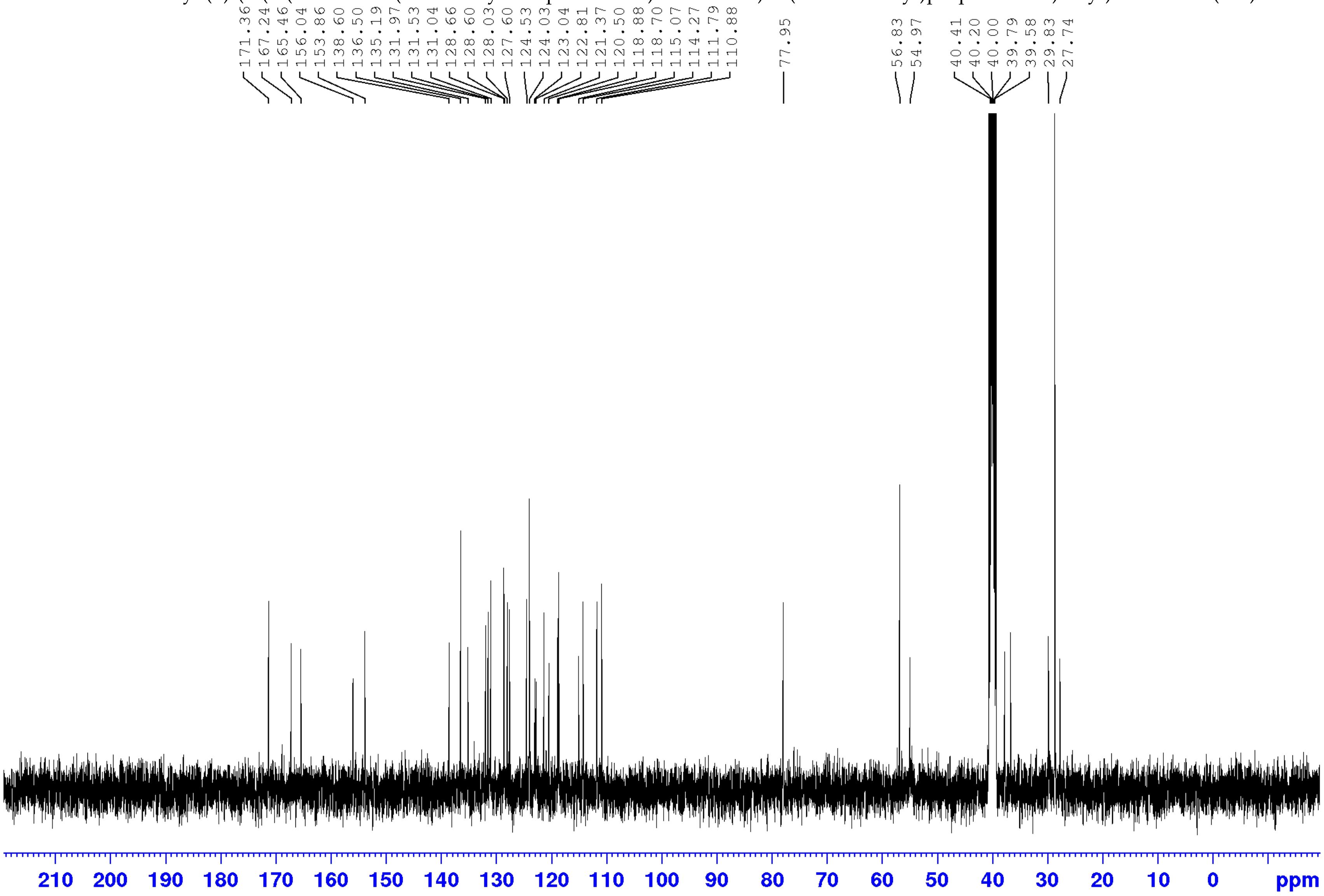

^{1}H NMR of *tert*-butyl (S)-(2-(2-(5-bromo-2-(3-methoxy-2-naphthamido)benzamido)-3-(1H-indol-3-yl)propanamido)ethyl)carbamate (10d)

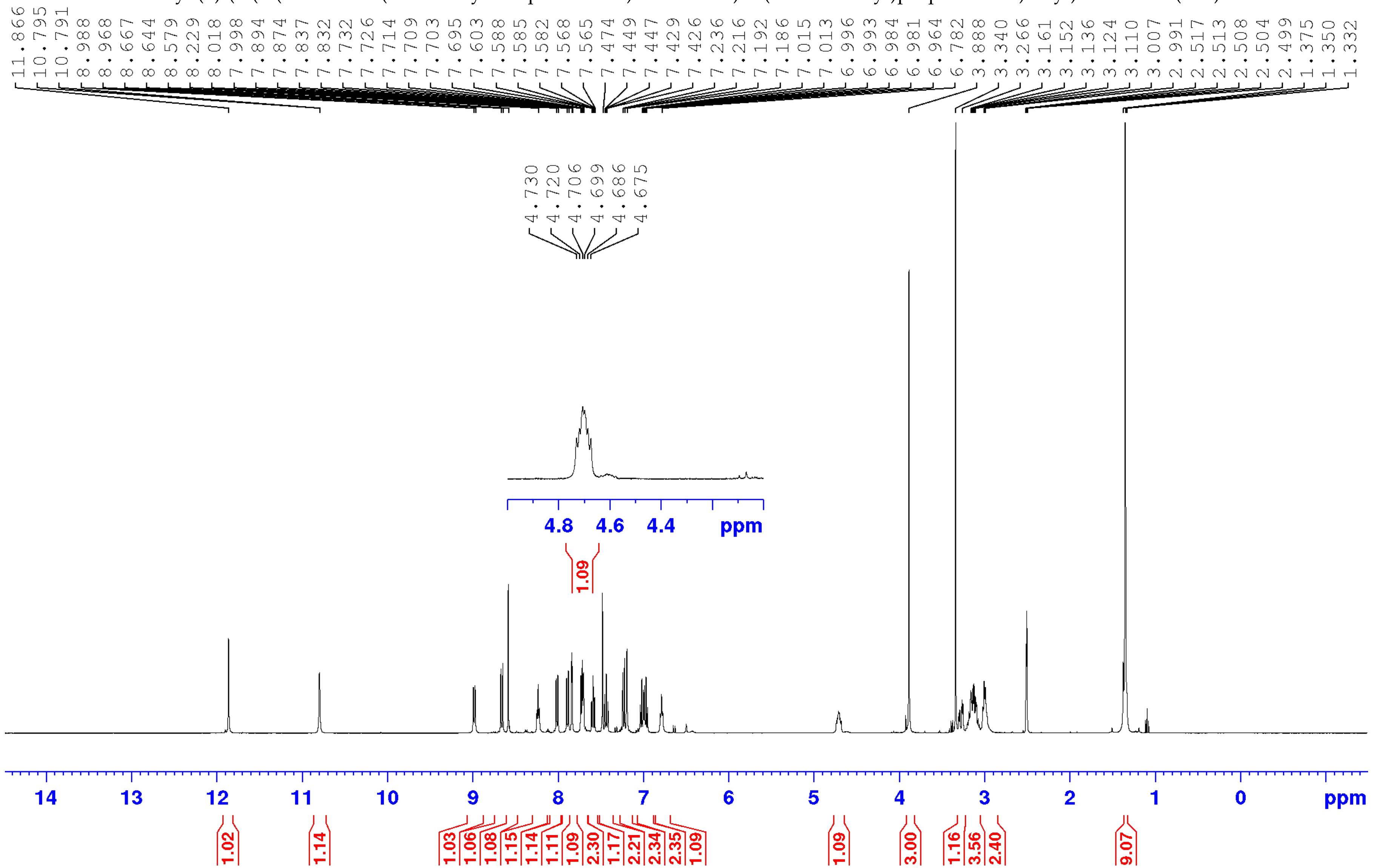

^{13}C NMR of *tert*-butyl (S)-(2-(2-(5-bromo-2-(3-methoxy-2-naphthamido)benzamido)-3-(1H-indol-3-yl)propanamido)ethyl)carbamate (10d)

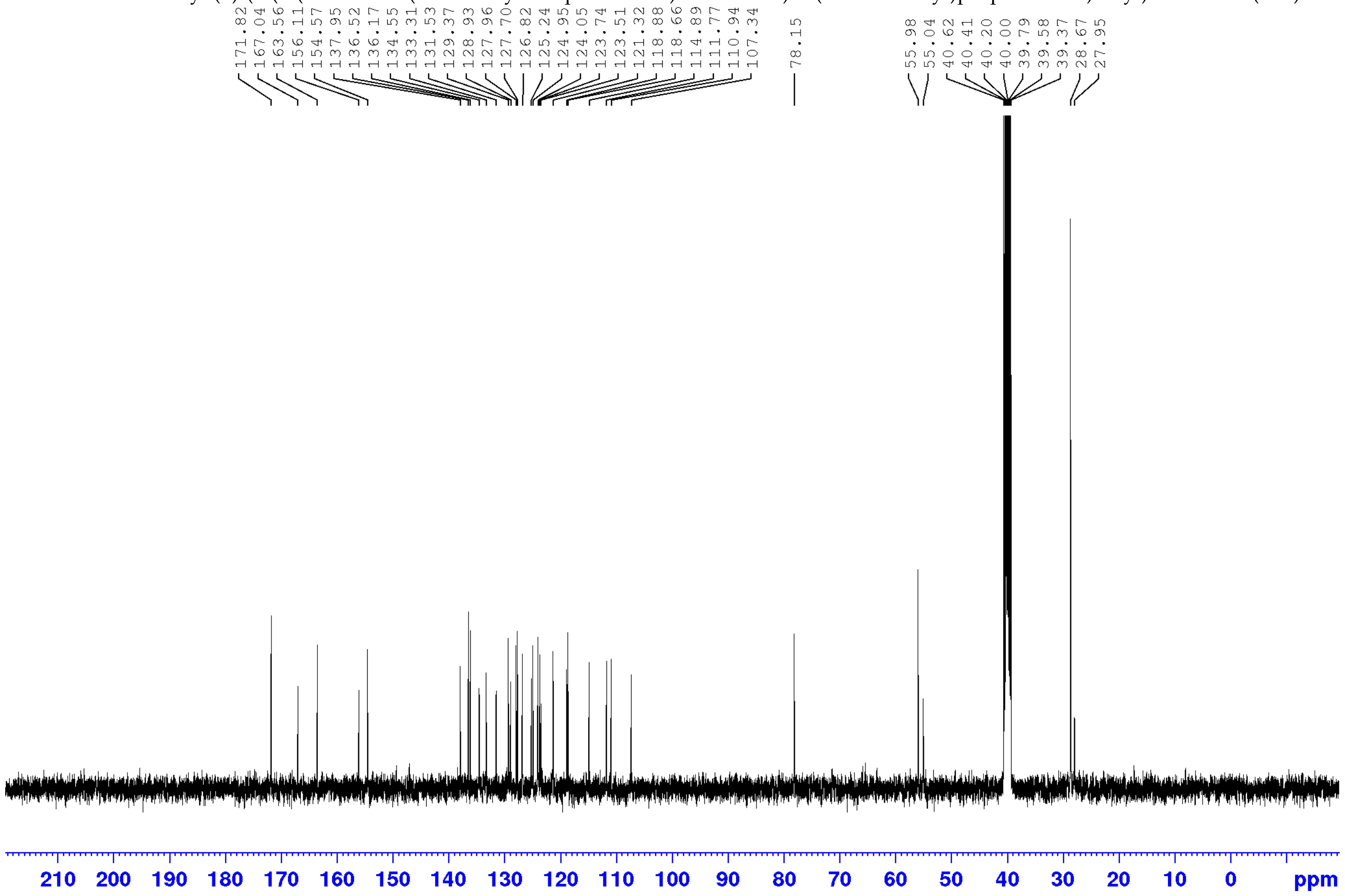

^{1}H NMR of *tert*-butyl (S)-(2-(2-(5-bromo-2-(quinoline-2-carboxamido)benzamido)-3-(1H-indol-3-yl)propanamido)ethyl)carbamate (10e)

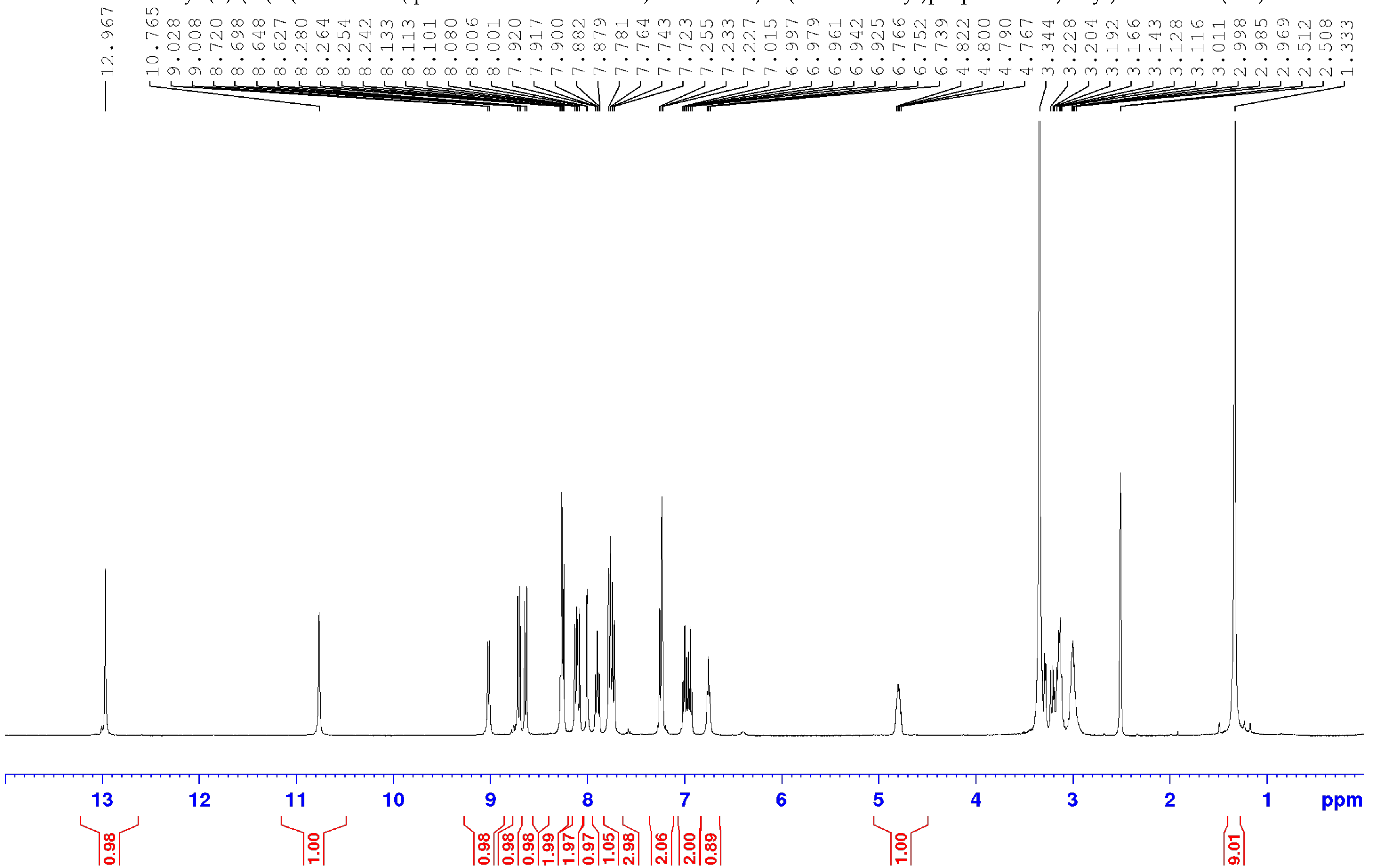

^{1}H NMR of *tert*-butyl (S)-(2-(2-(5-bromo-2-(1H-indole-2-carboxamido)benzamido)-3-(1H-indol-3-yl)propanamido)ethyl)carbamate (10f)

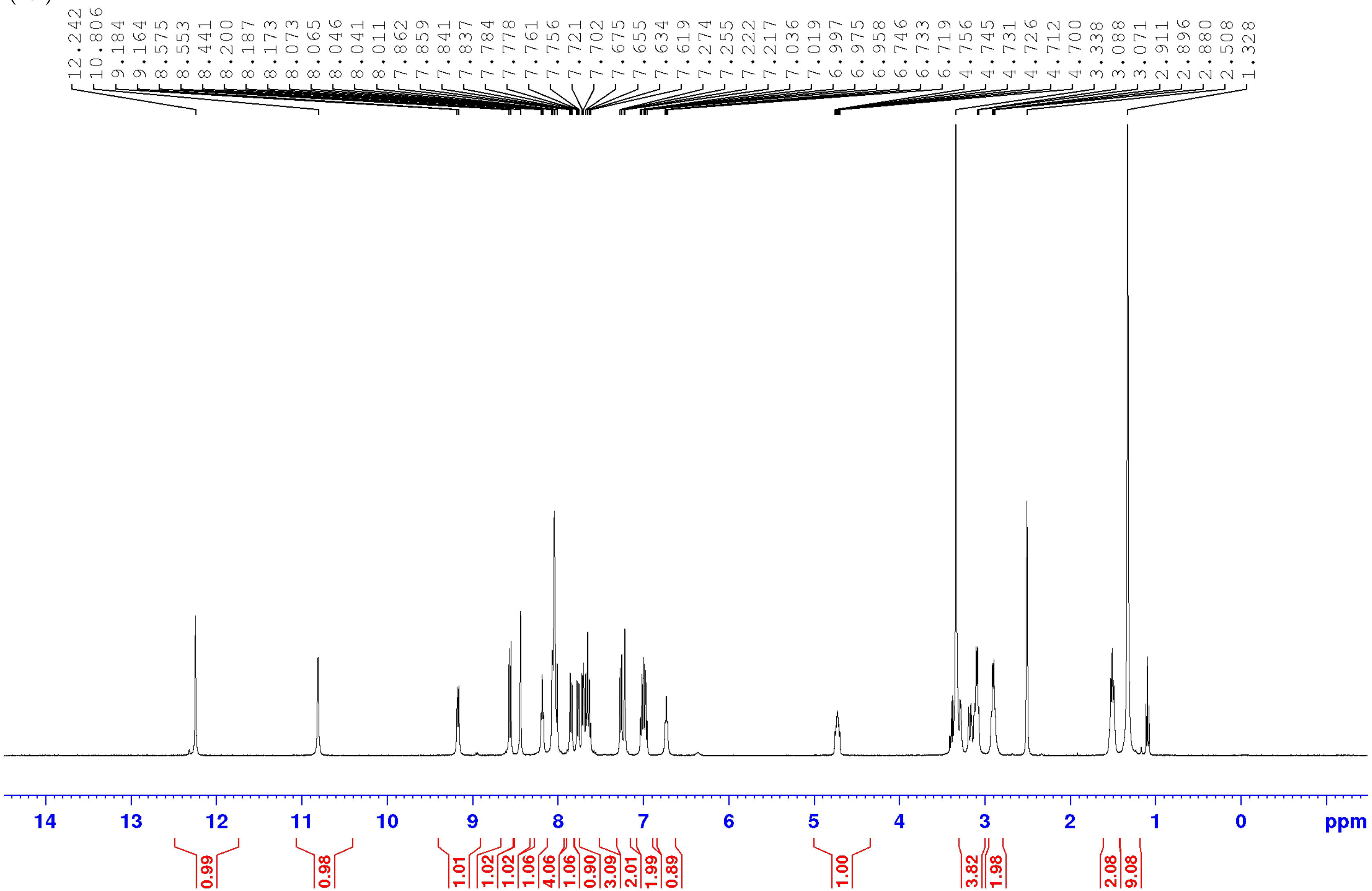

^{13}C NMR of *tert*-butyl (S)-(2-(2-(5-bromo-2-(1H-indole-2-carboxamido)benzamido)-3-(1H-indol-3-yl)propanamido)ethyl)carbamate (10f)

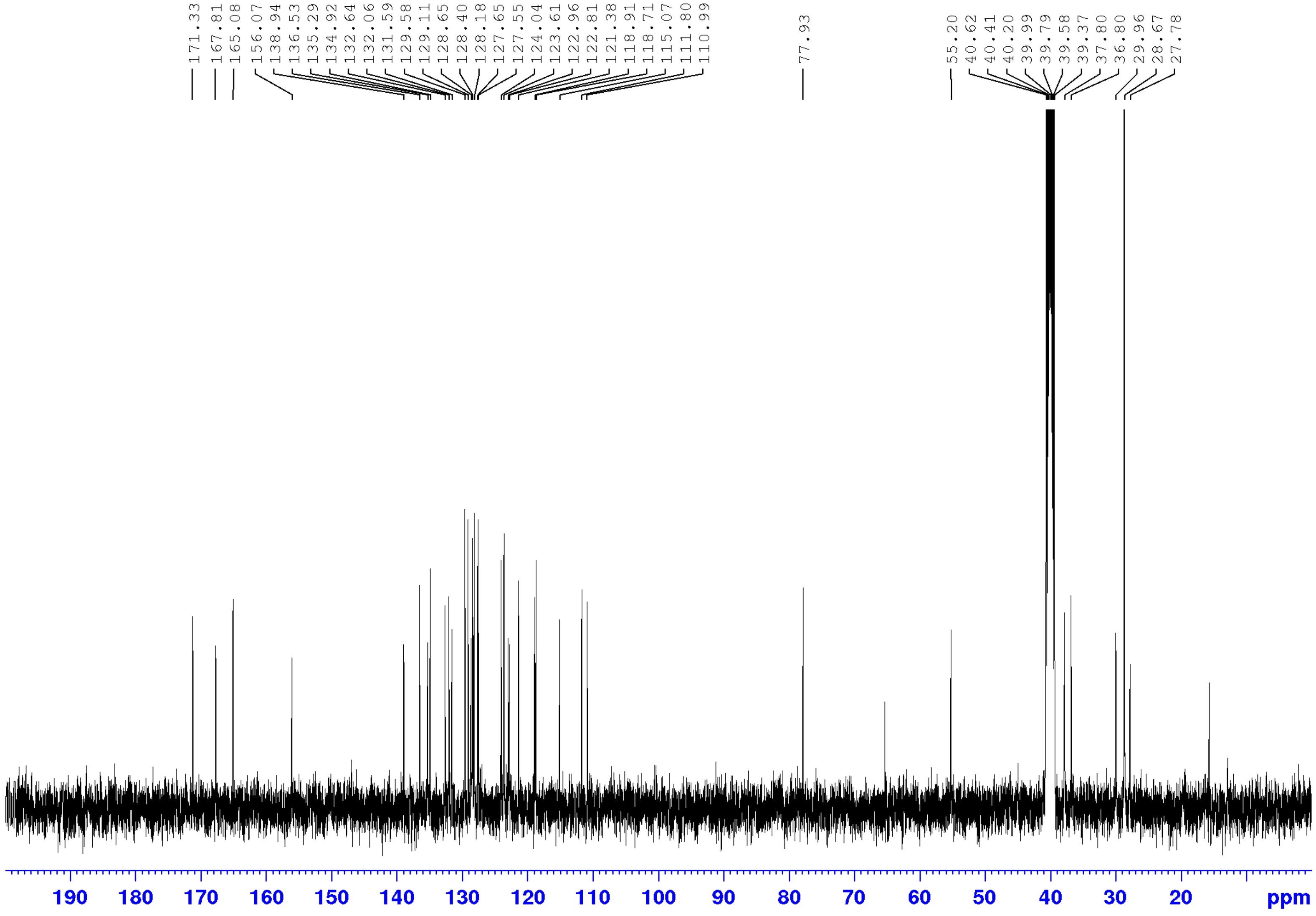

^{1}H NMR of *tert*-butyl (S)-(2-(2-(5-bromo-2-(thiophene-2-carboxamido)benzamido)-3-(1H-indol-3-yl)propanamido)ethyl)carbamate (10g)

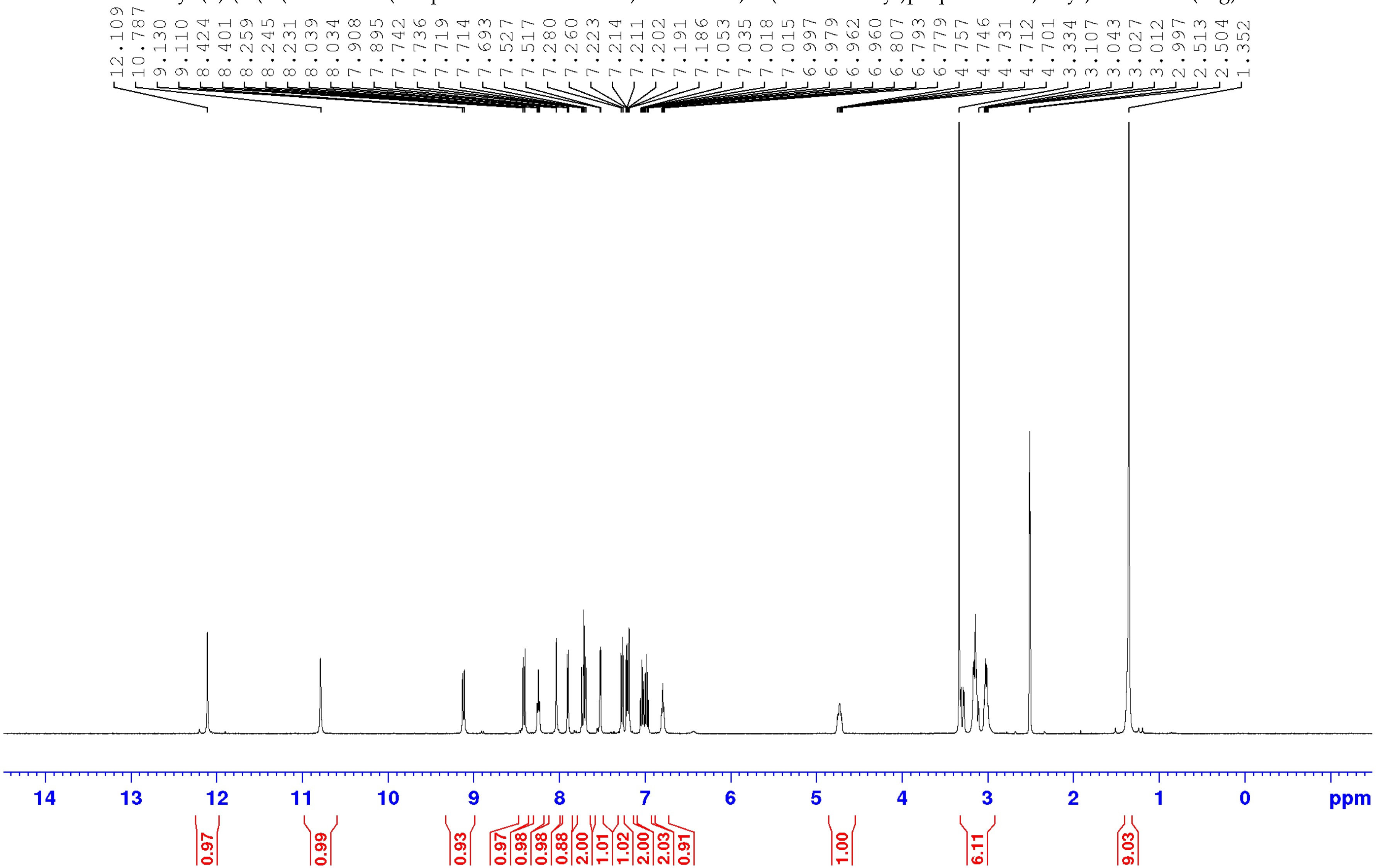

^{13}C NMR of *tert*-butyl (S)-(2-(2-(5-bromo-2-(thiophene-2-carboxamido)benzamido)-3-(1H-indol-3-yl)propanamido)ethyl)carbamate (10g)

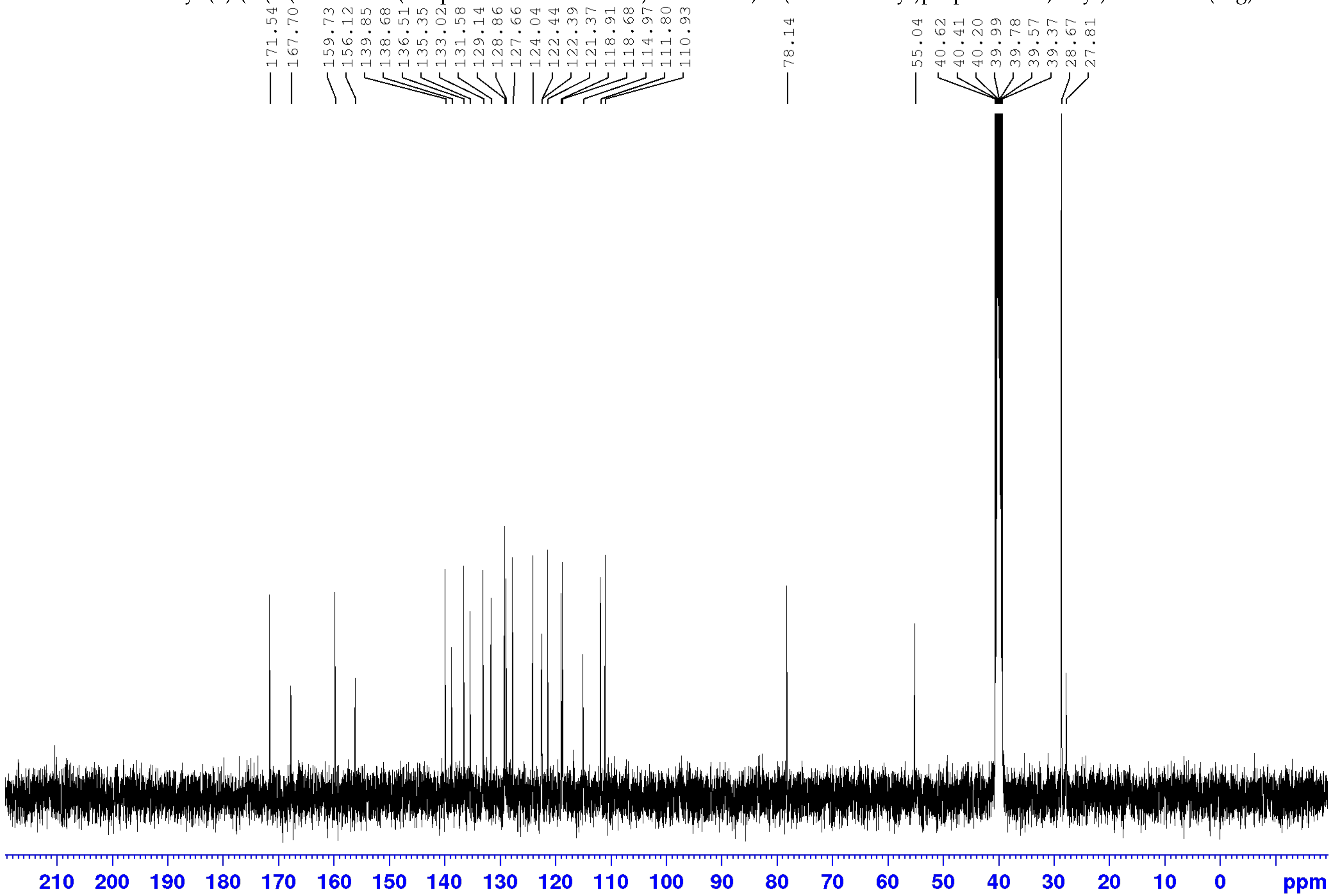

^{1}H NMR of *tert*-butyl (S)-(2-(2-(5-bromo-2-(thiophene-3-carboxamido)benzamido)-3-(1H-indol-3-yl)propanamido)ethyl)carbamate (10h)

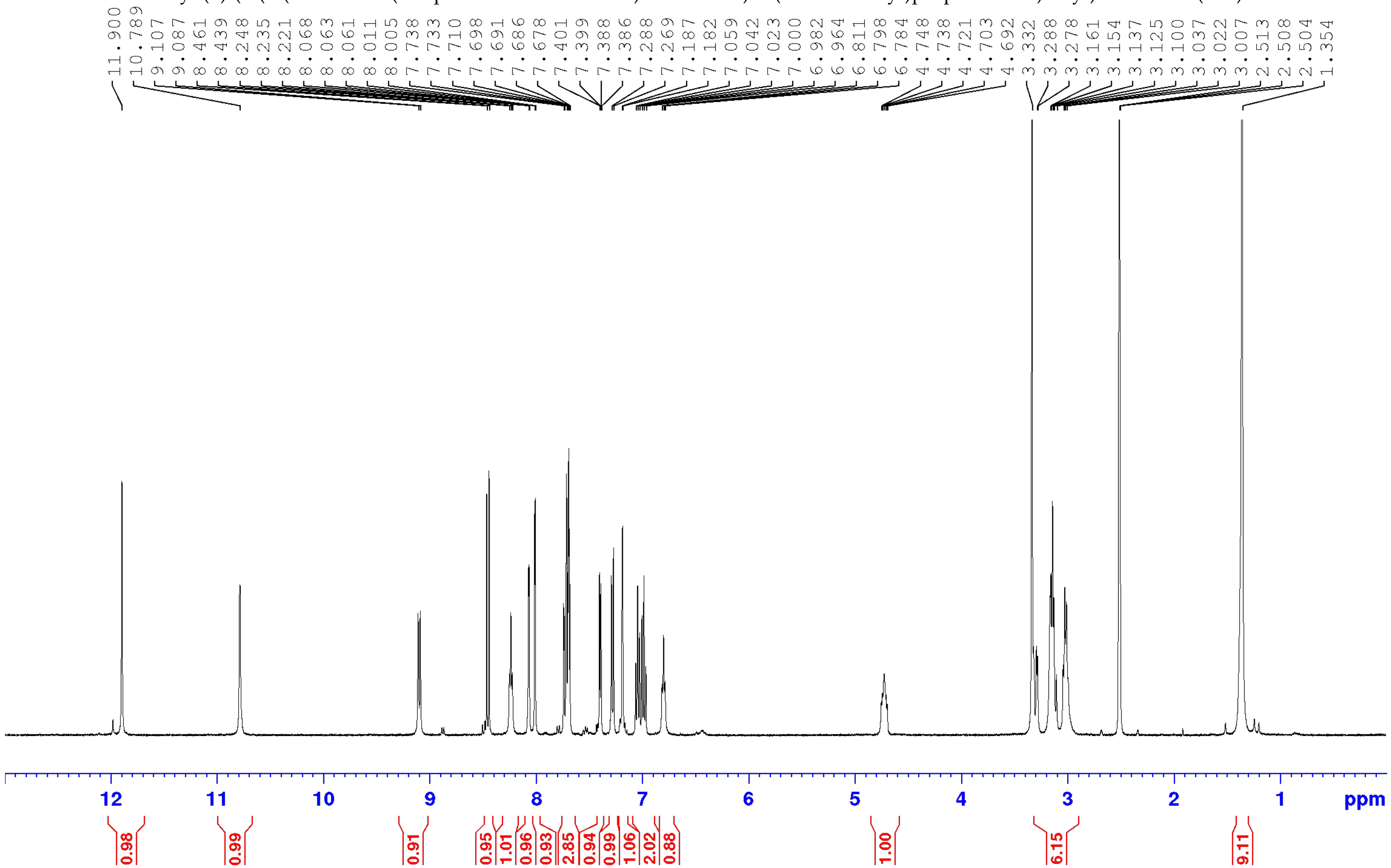

^{13}C NMR of *tert*-butyl (S)-(2-(2-(5-bromo-2-(thiophene-3-carboxamido)benzamido)-3-(1H-indol-3-yl)propanamido)ethyl)carbamate (10h)

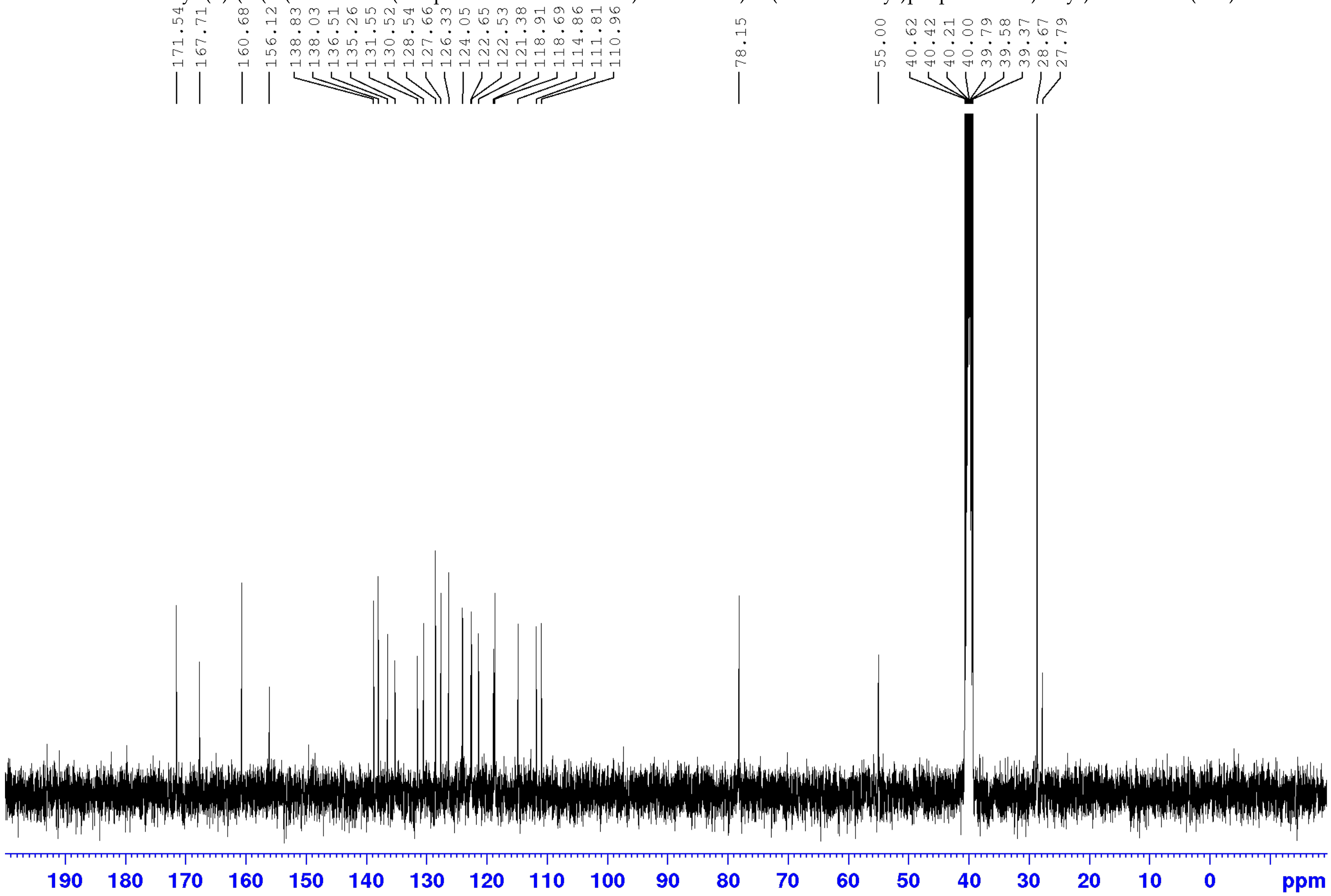

^{1}H NMR of *tert*-butyl (S)-(2-(2-(2-([1,1'-biphenyl]-2-carboxamido)-5-bromobenzamido)-3-(1H-indol-3-yl)propanamido)ethyl)carbamate (10i)

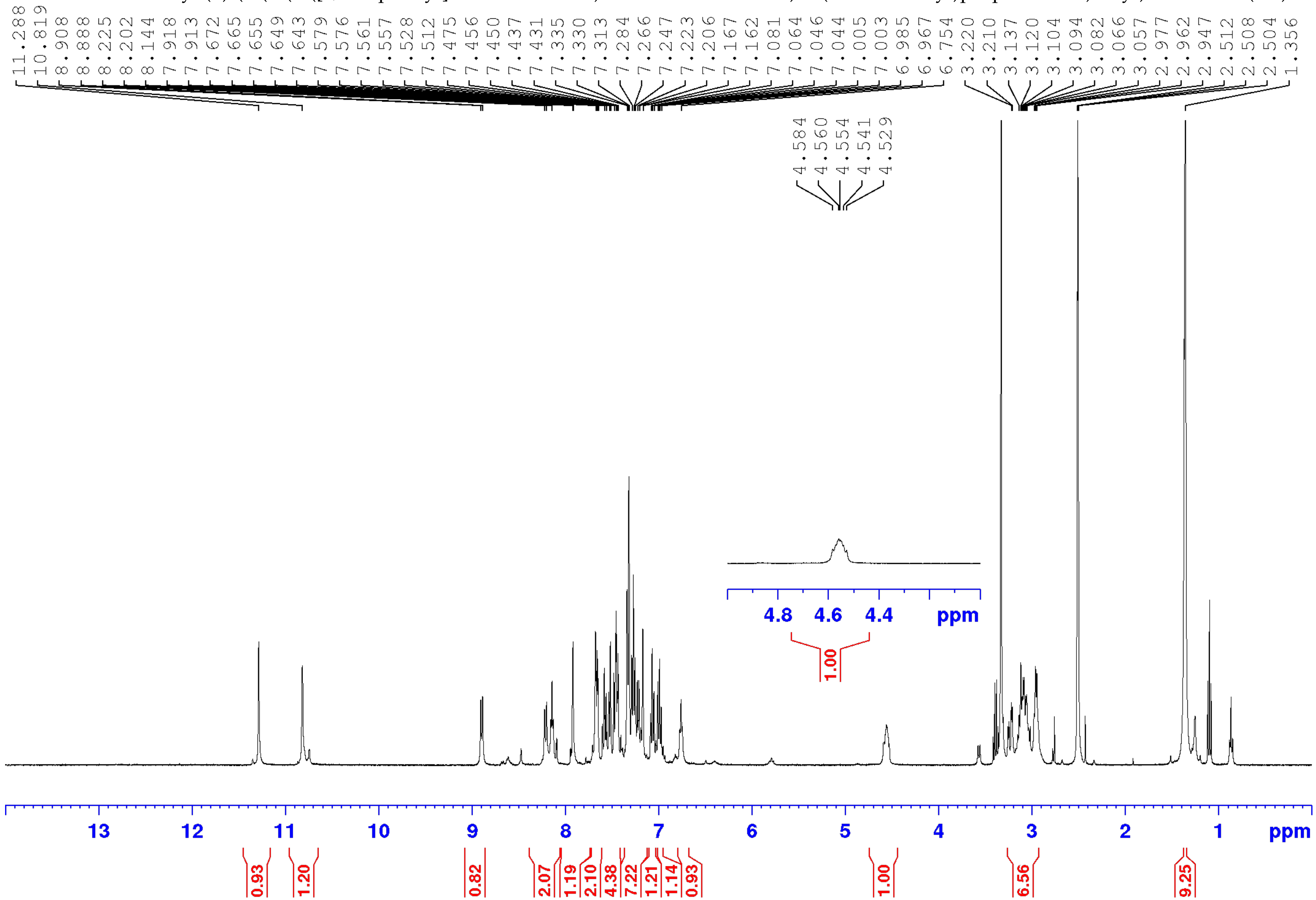

^{13}C NMR of *tert*-butyl (S)-(2-(2-(2-([1,1'-biphenyl]-2-carboxamido)-5-bromobenzamido)-3-(1H-indol-3-yl)propanamido)ethyl)carbamate (10i)

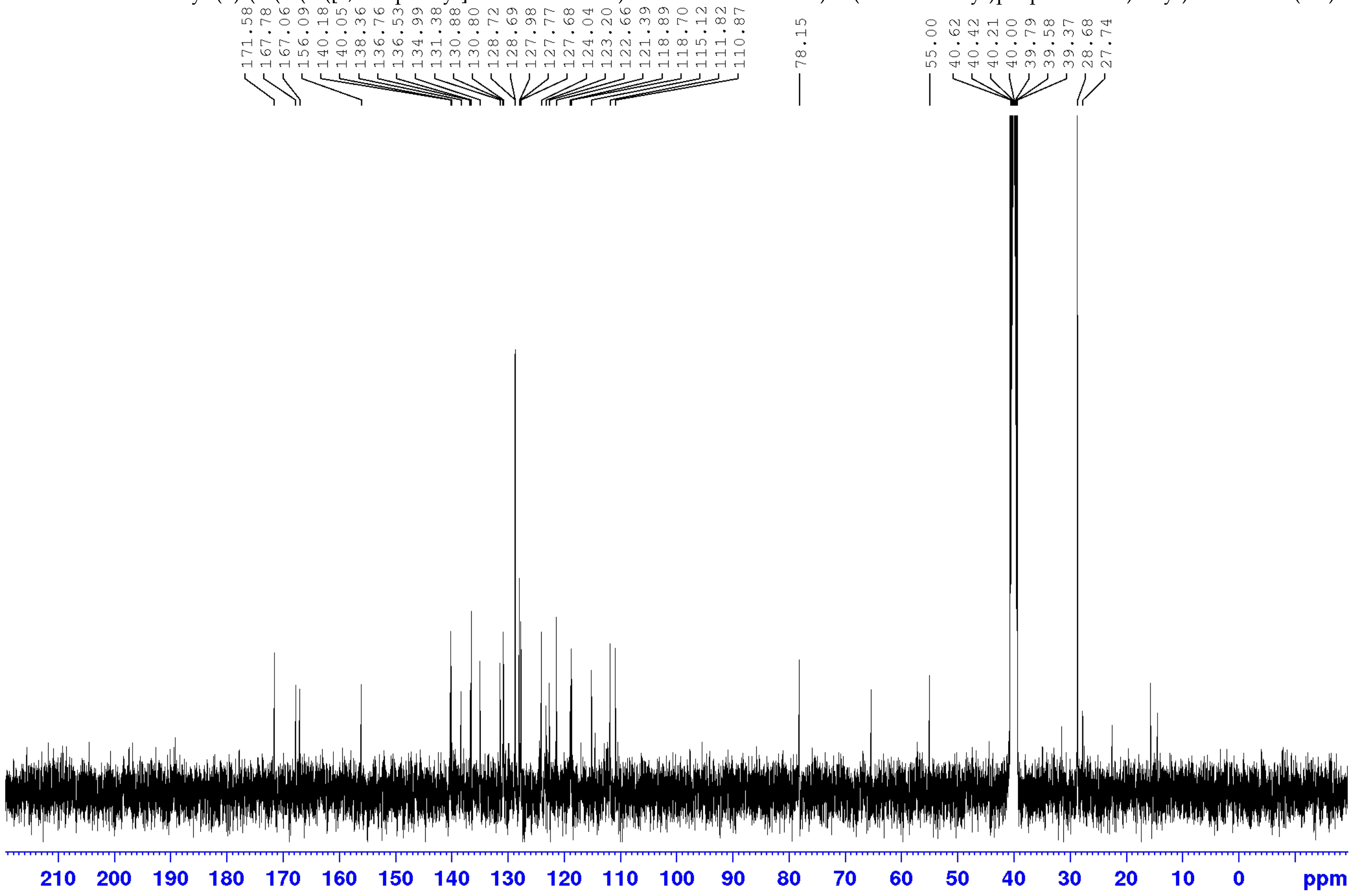

^{1}H NMR of *tert*-butyl (S)-(2-(2-(2-([1,1'-biphenyl]-3-carboxamido)-5-bromobenzamido)-3-(1*H*-indol-3-yl)propanamido)ethyl)carbamate (10j)

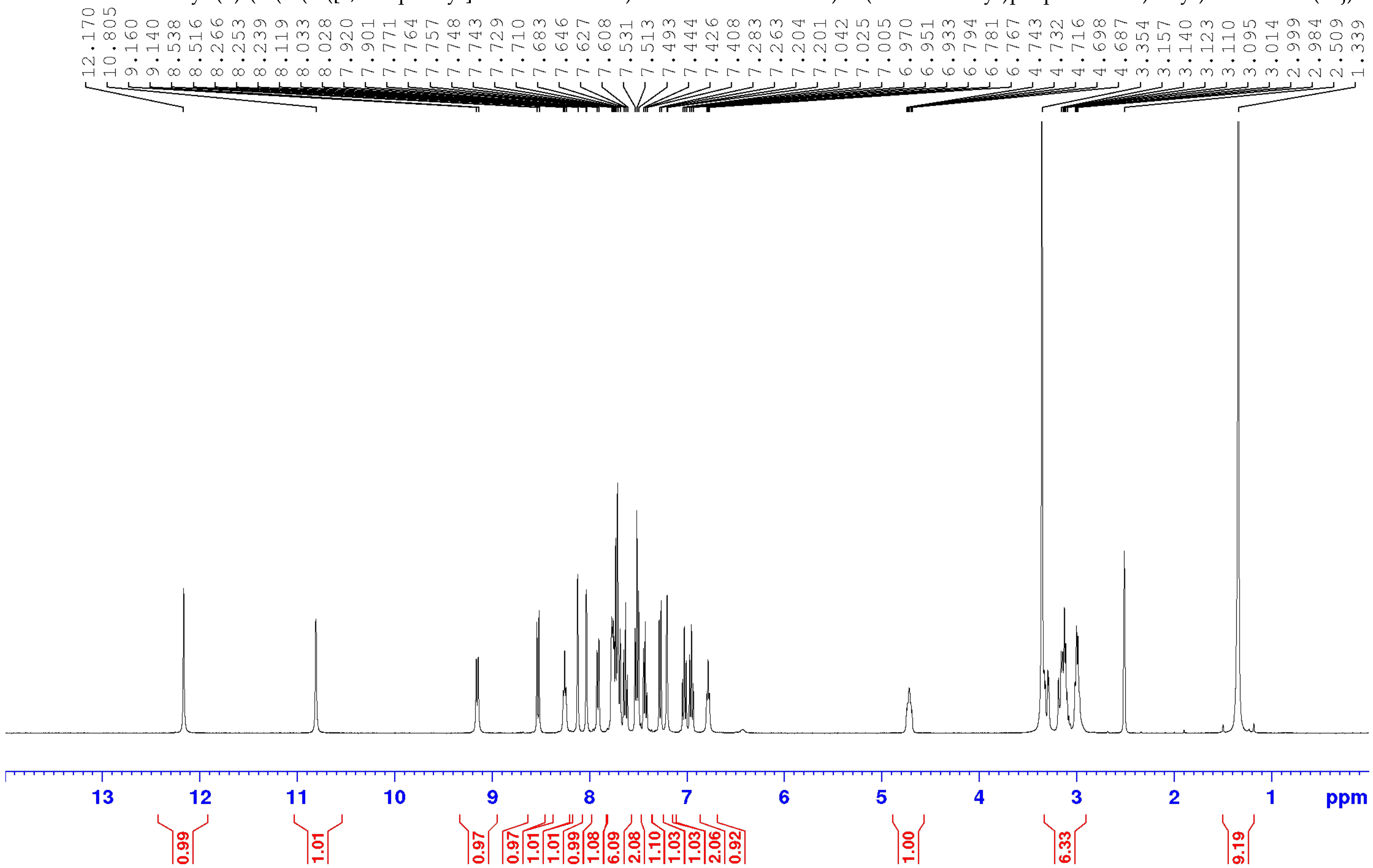

^{13}C NMR of *tert*-butyl (S)-(2-(2-(2-([1,1'-biphenyl]-3-carboxamido)-5-bromobenzamido)-3-(1*H*-indol-3-yl)propanamido)ethyl)carbamate (10j)

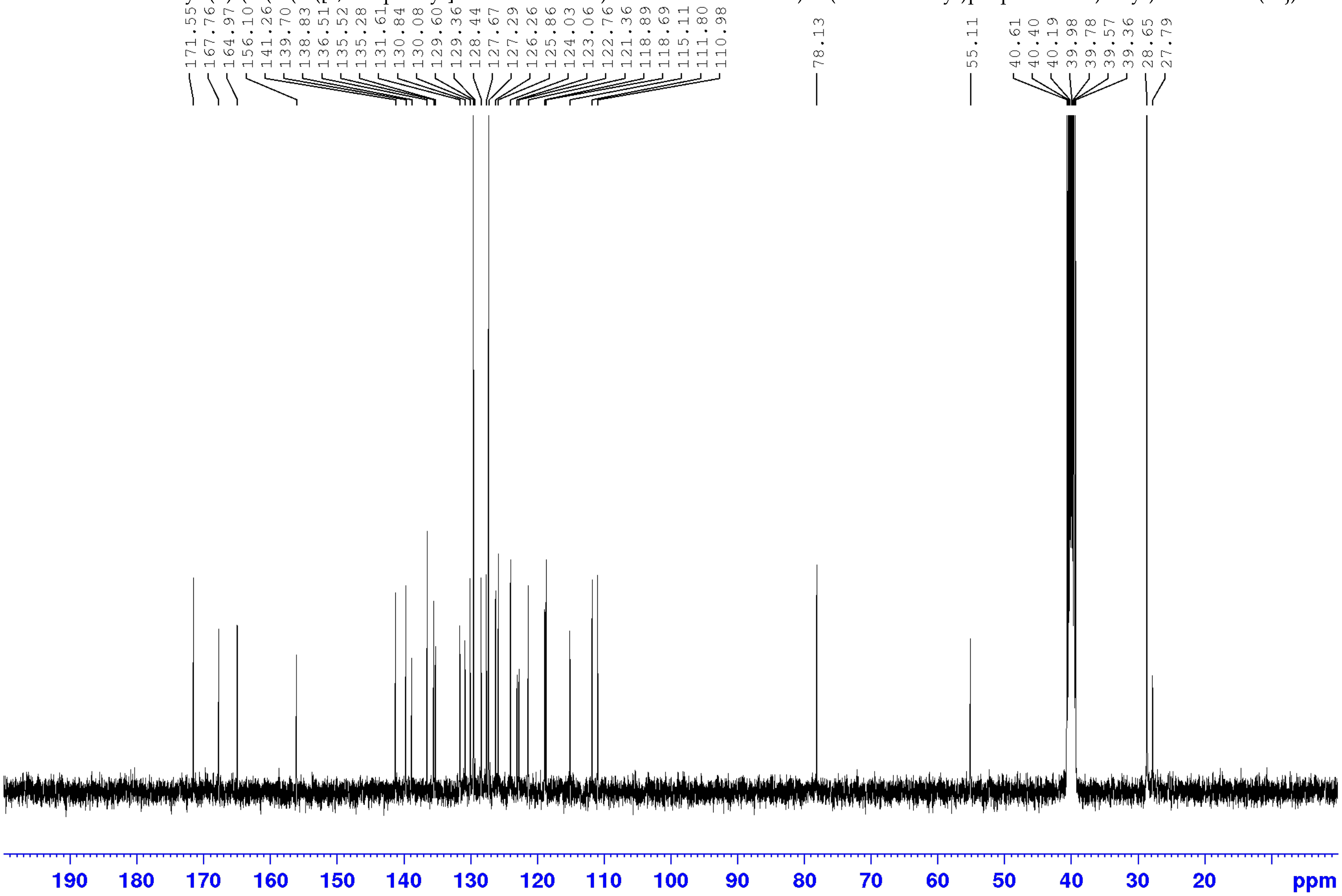

^{1}H NMR of *tert*-butyl (S)-(2-(2-(2-([1,1'-biphenyl]-4-carboxamido)-5-bromobenzamido)-3-(1*H*-indol-3-yl)propanamido)ethyl)carbamate (10k)

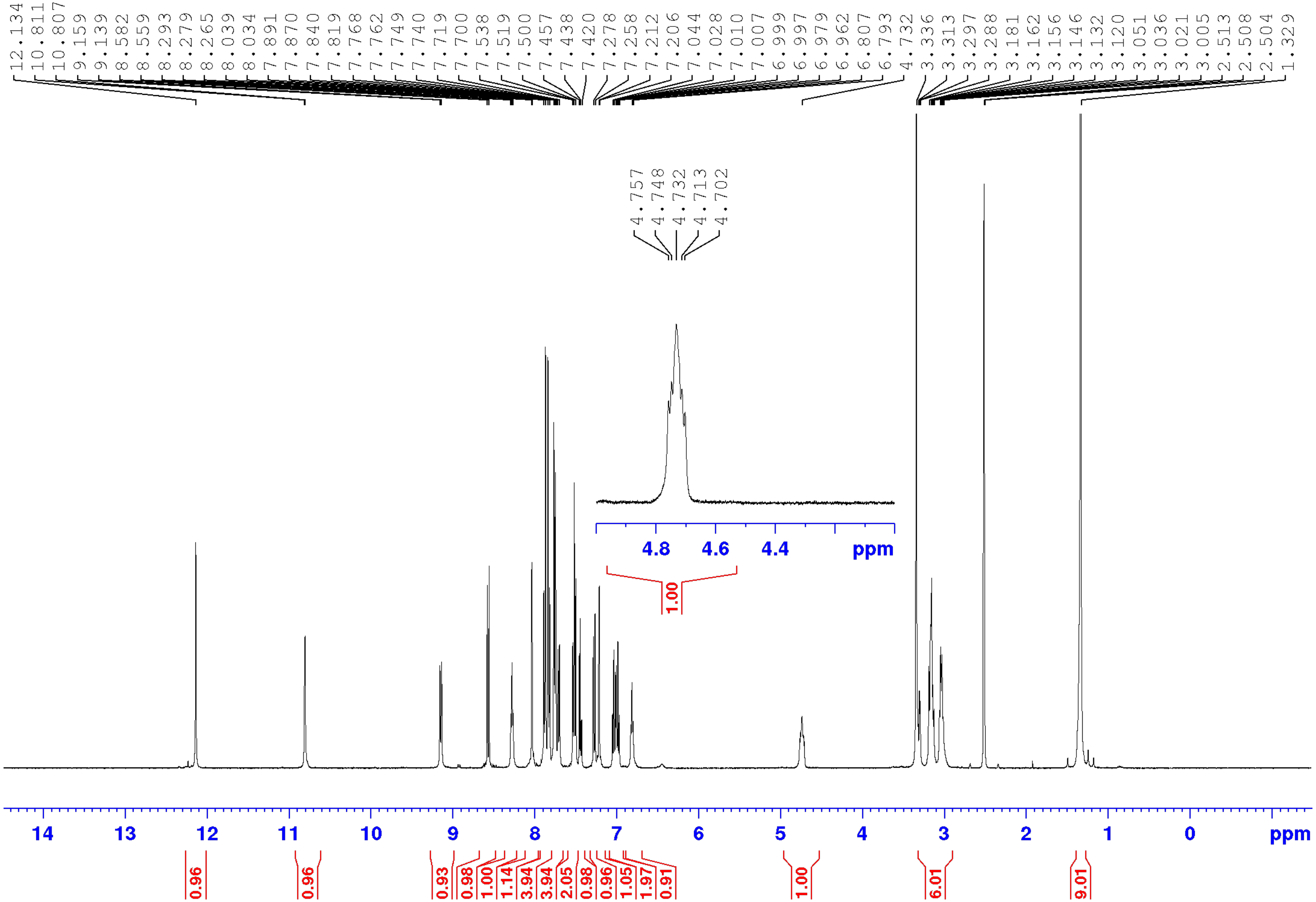

^{13}C NMR of *tert*-butyl (S)-(2-(2-(2-([1,1'-biphenyl]-4-carboxamido)-5-bromobenzamido)-3-(1*H*-indol-3-yl)propanamido)ethyl)carbamate (10k)

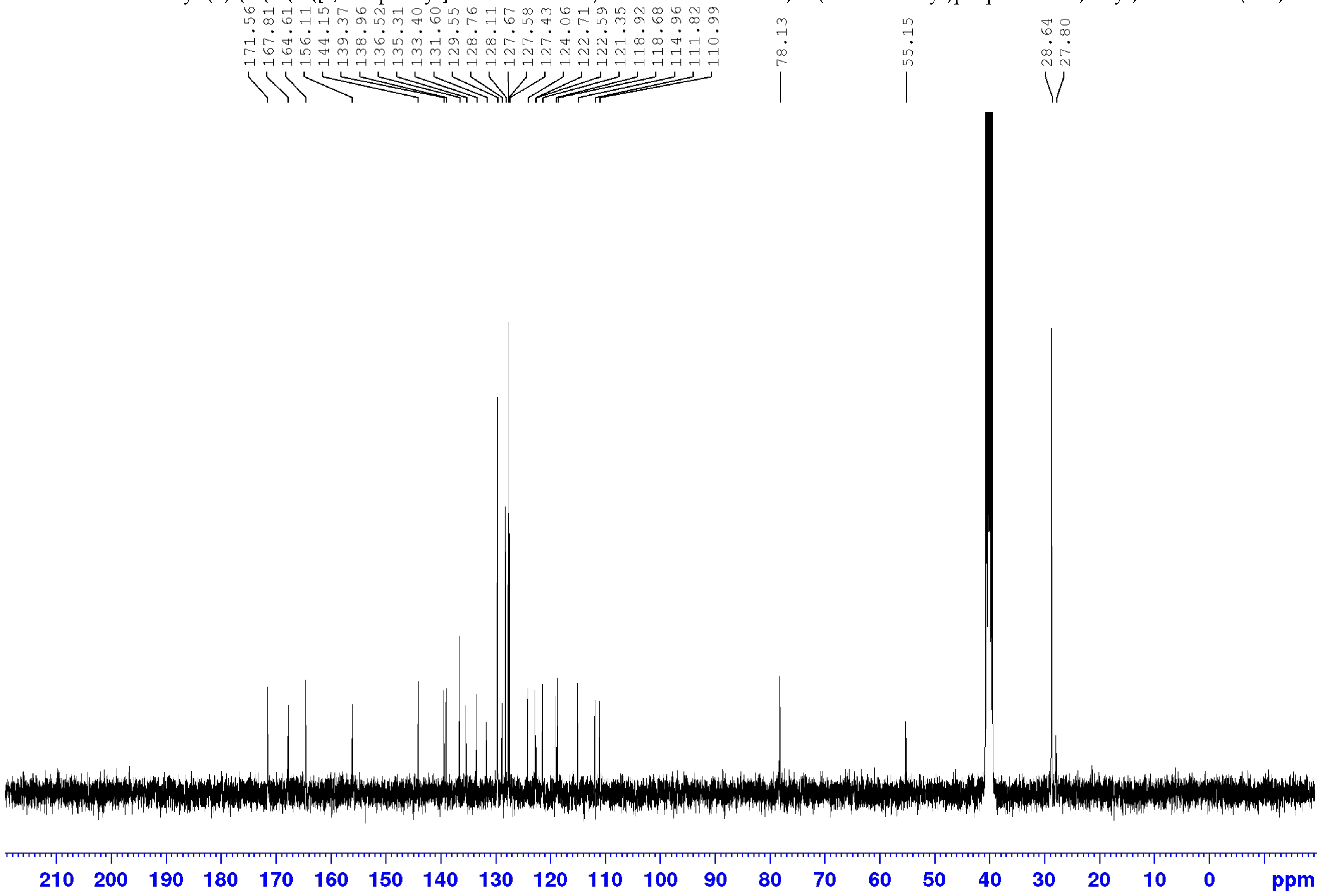

^{1}H NMR of *tert*-butyl (S)-(3-(2-(2-(2-naphthamido)-5-bromobenzamido)-3-(1H-indol-3-yl)propanamido)propyl)carbamate (11a)

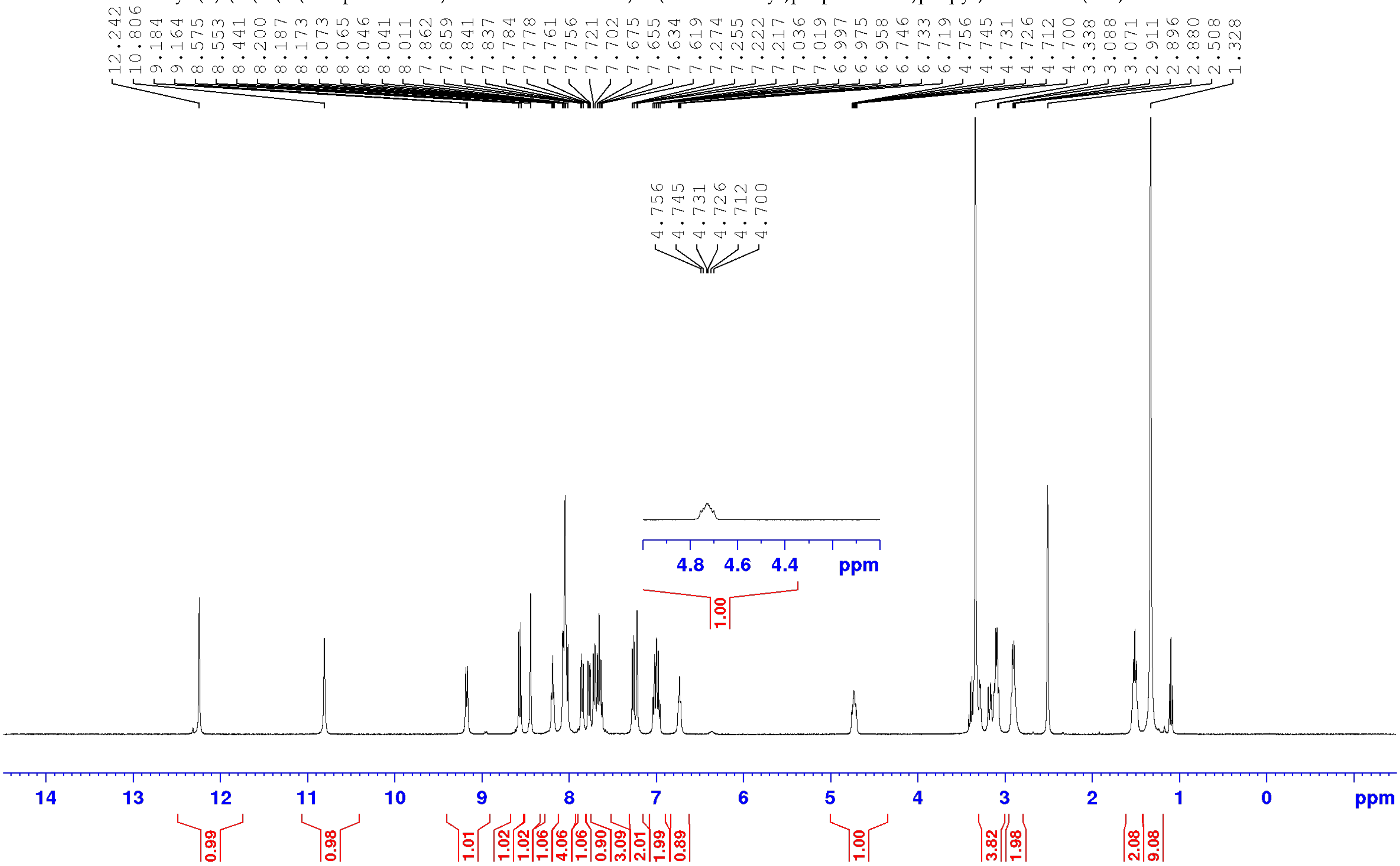

^{13}C NMR of *tert*-butyl (S)-(3-(2-(2-(2-naphthamido)-5-bromobenzamido)-3-(1H-indol-3-yl)propanamido)propyl)carbamate (11a)

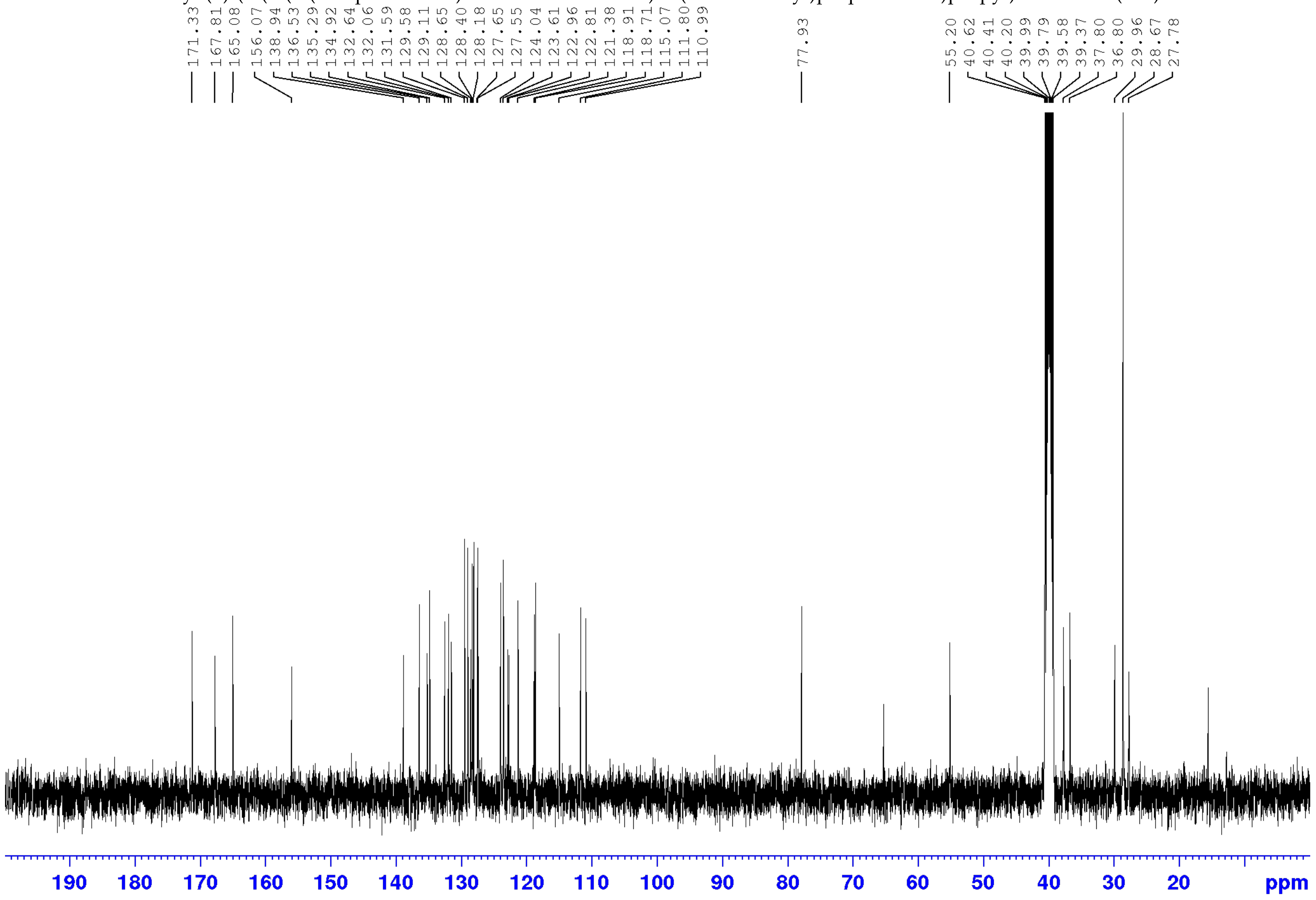

^{1}H NMR of *tert*-butyl (S)-(3-(2-(5-bromo-2-(2-(naphthalen-1-yl)acetamido)benzamido)-3-(1*H*-indol-3-yl)propanamido)propyl)carbamate (11b)

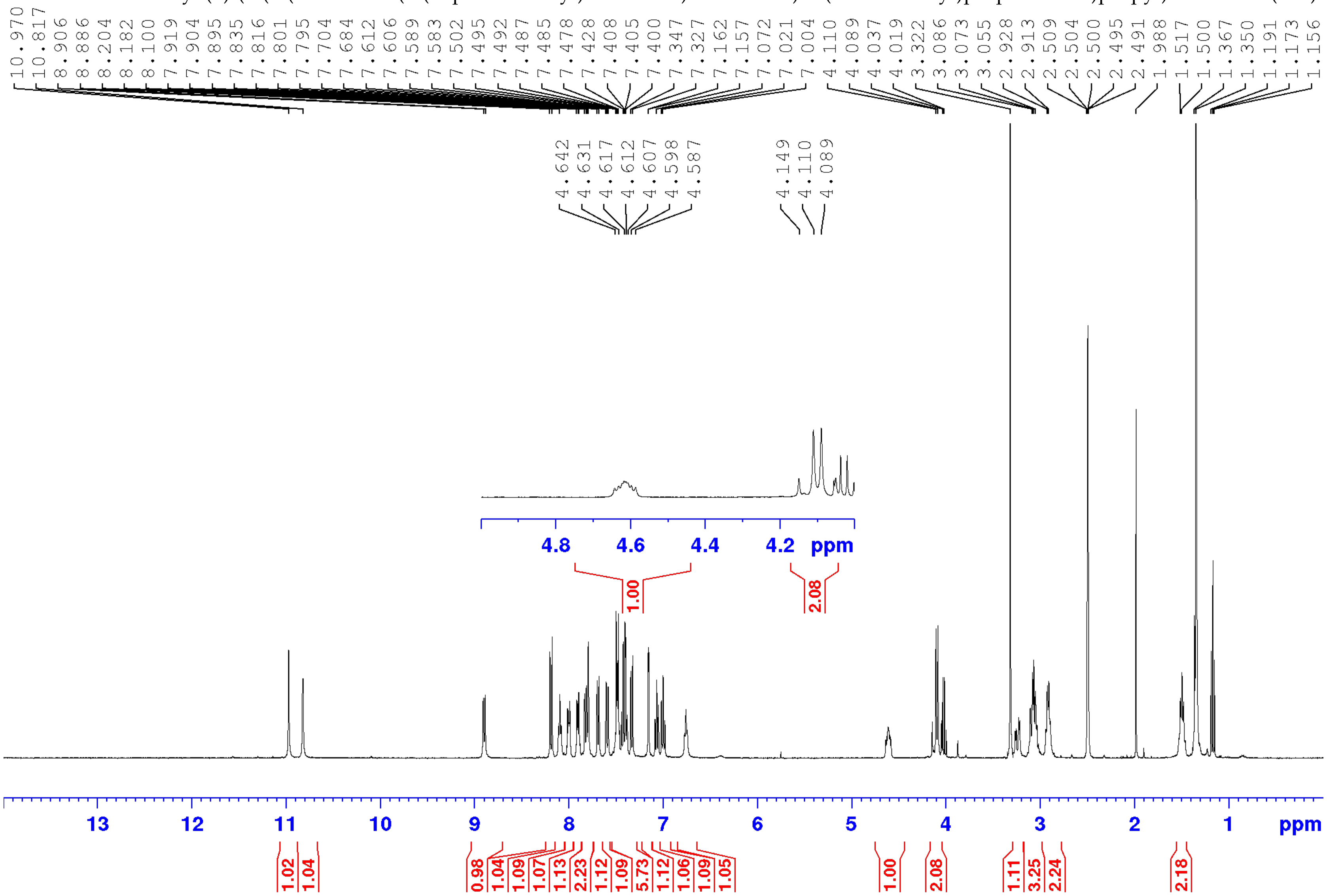

^{13}C NMR of *tert*-butyl (S)-(3-(2-(5-bromo-2-(2-(naphthalen-1-yl)acetamido)benzamido)-3-(1*H*-indol-3-yl)propanamido)propyl)carbamate (11b)

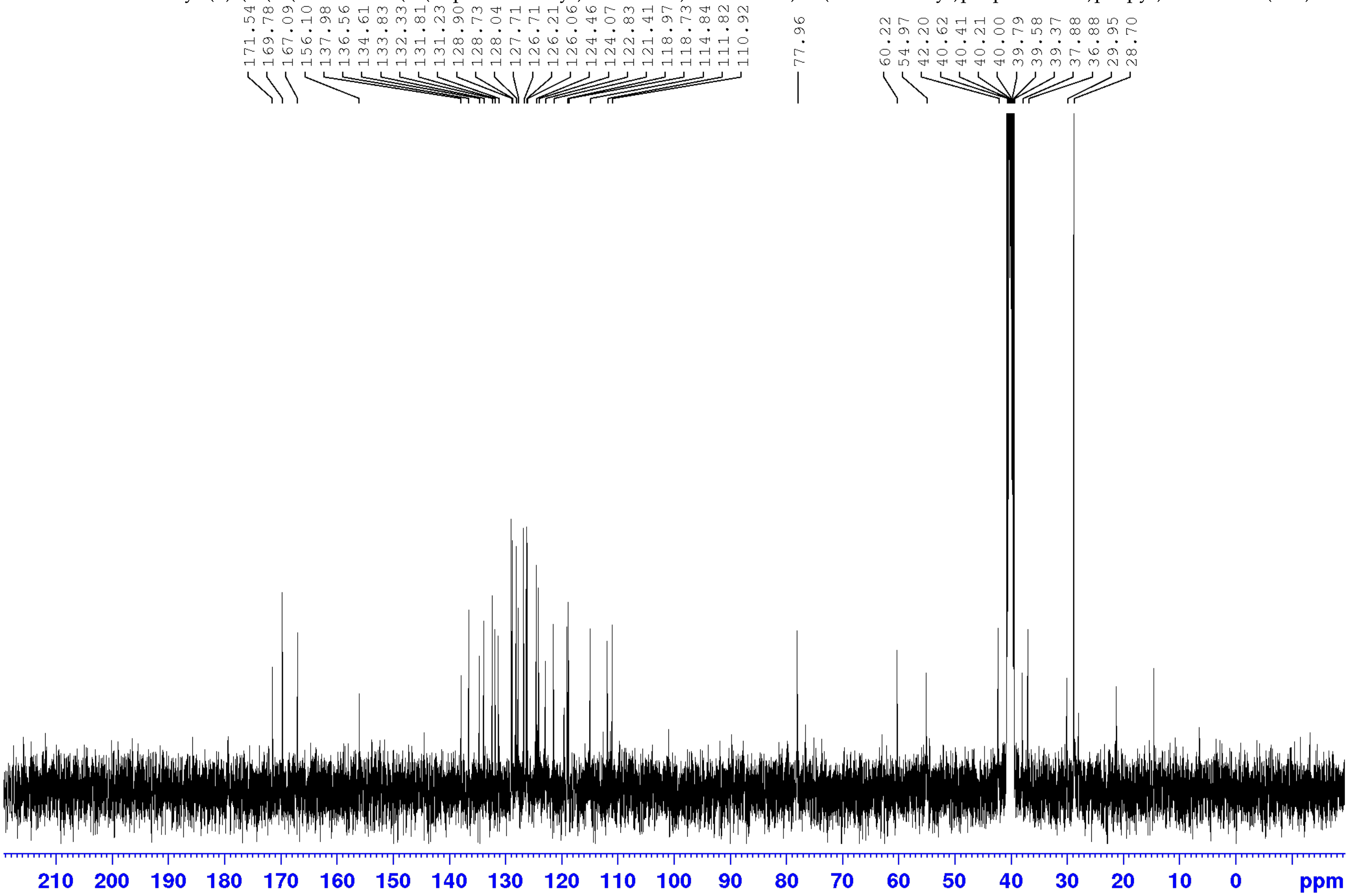

^{1}H NMR of *tert*-butyl (S)-(3-(2-(5-bromo-2-(2-methoxy-1-naphthamido)benzamido)-3-(1*H*-indol-3-yl)propanamido)propyl)carbamate (11c)

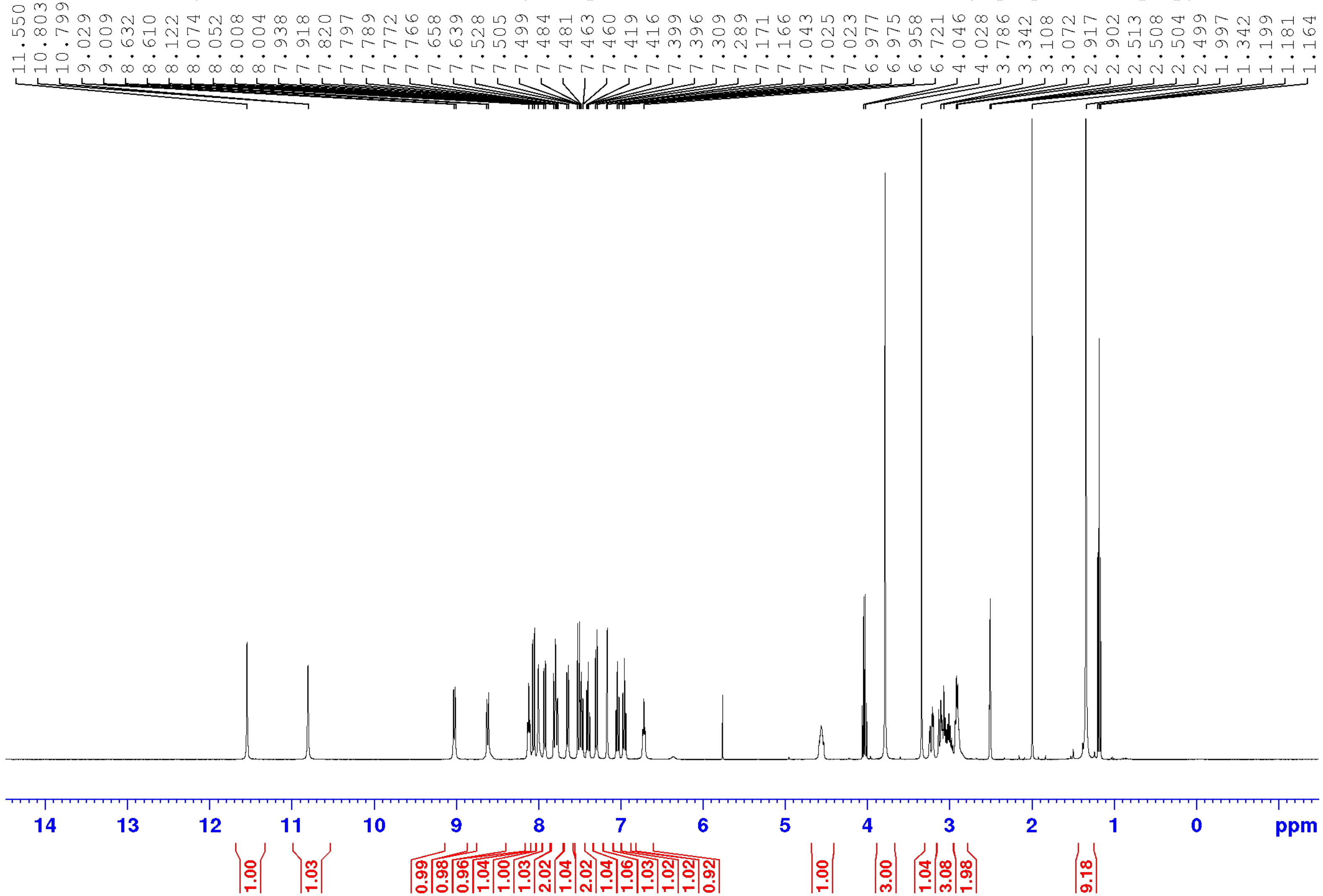

^{13}C NMR of *tert*-butyl (S)-(3-(2-(5-bromo-2-(2-methoxy-1-naphthamido)benzamido)-3-(1*H*-indol-3-yl)propanamido)propyl)carbamate (11c)

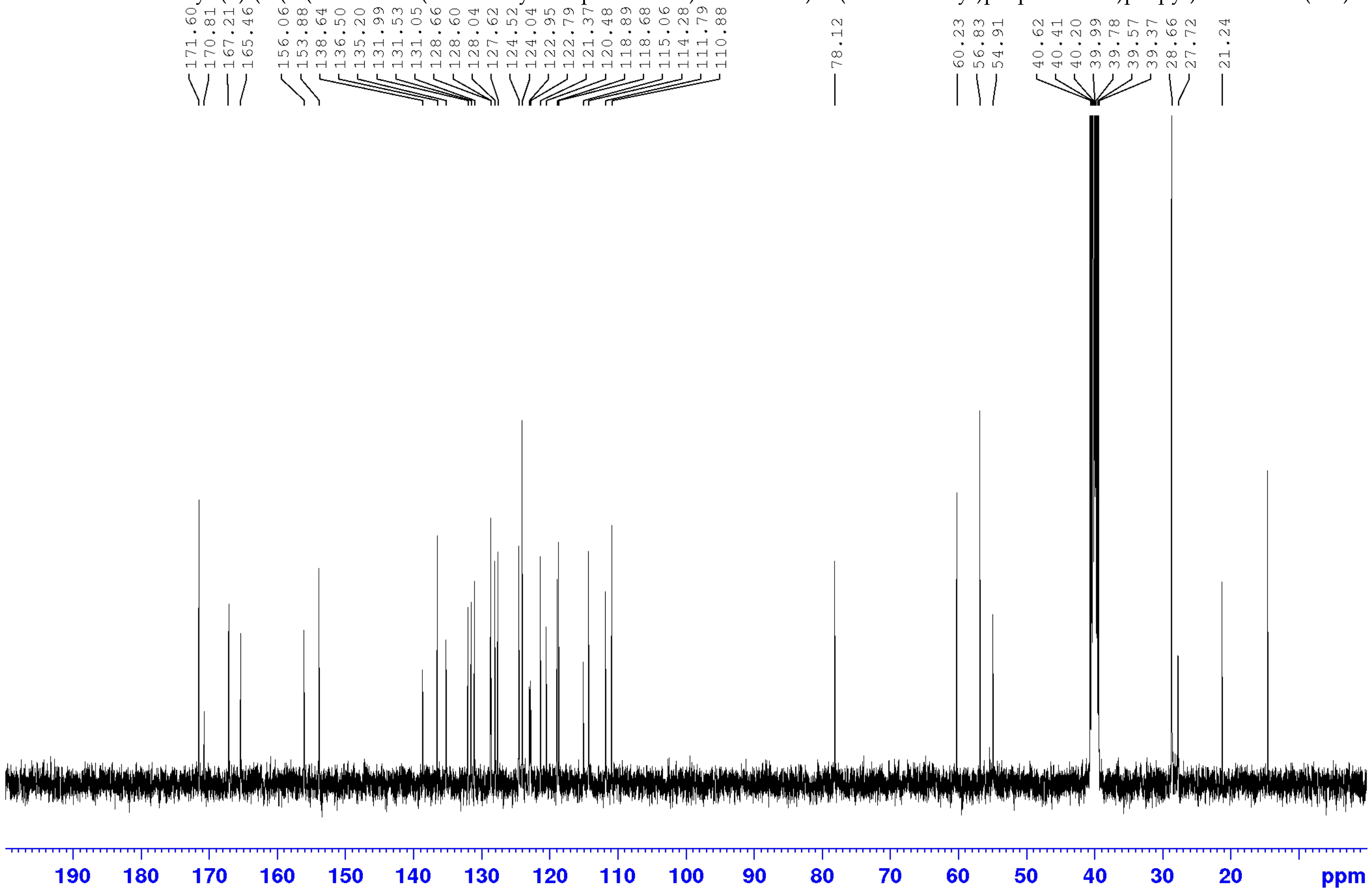

^{1}H NMR of *tert*-butyl (S)-(3-(2-(5-bromo-2-(3-methoxy-2-naphthamido)benzamido)-3-(1H-indol-3-yl)propanamido)propyl)carbamate (11d)

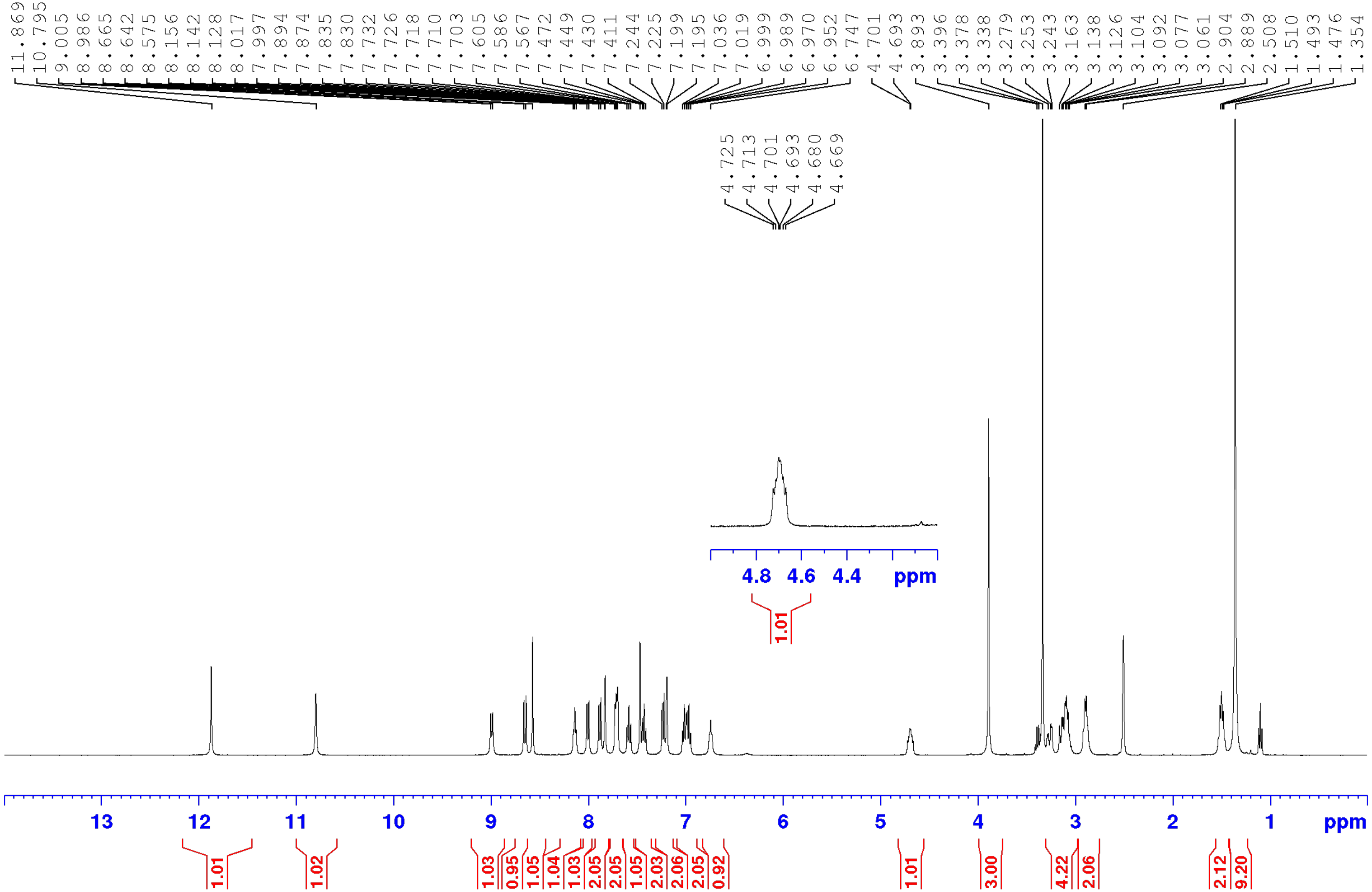

^{13}C NMR of *tert*-butyl (S)-(3-(2-(5-bromo-2-(3-methoxy-2-naphthamido)benzamido)-3-(1H-indol-3-yl)propanamido)propyl)carbamate (11d)

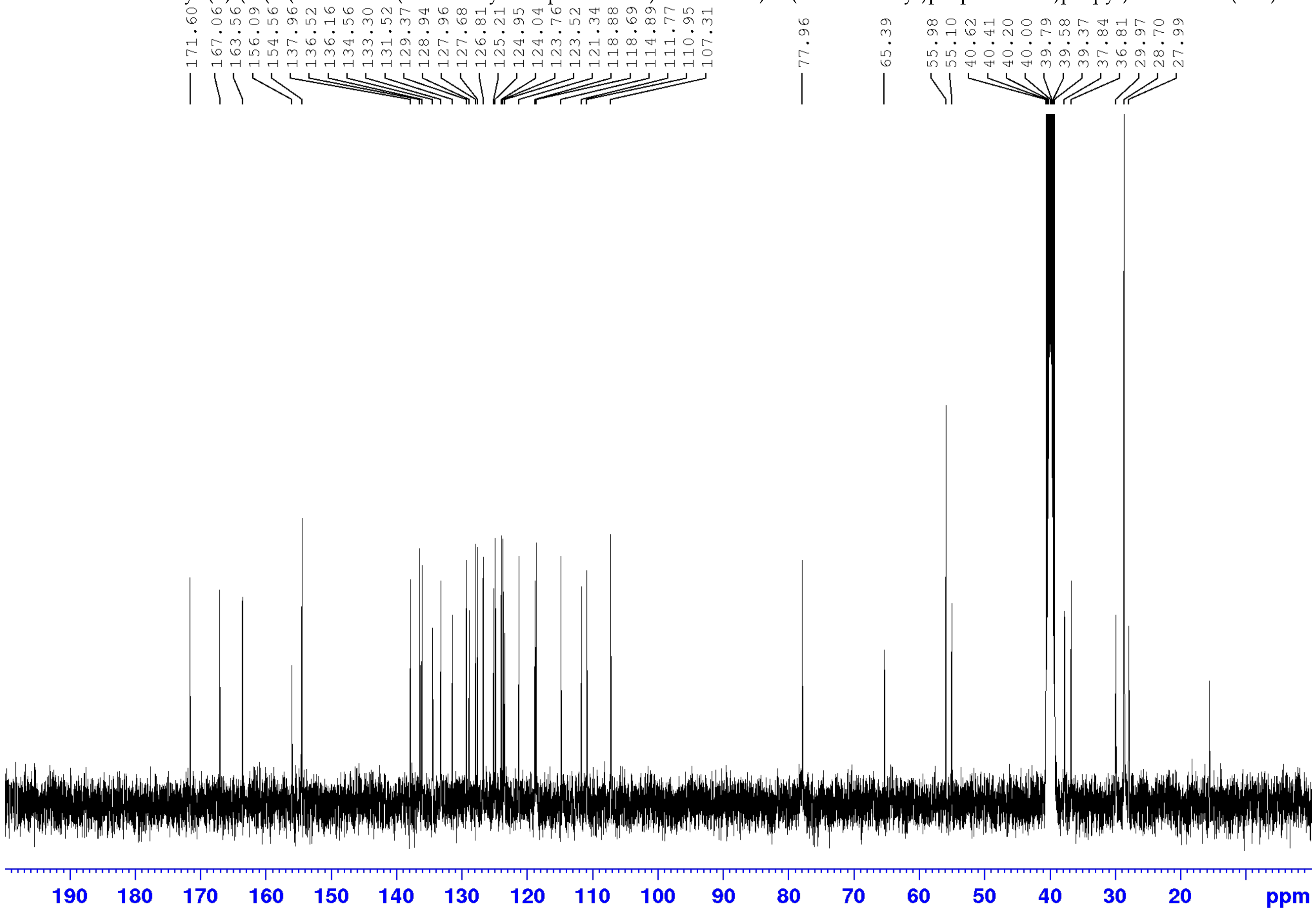

^{1}H NMR of *tert*-butyl (S)-(3-(2-(5-bromo-2-(quinoline-2-carboxamido)benzamido)-3-(1*H*-indol-3-yl)propanamido)propyl)carbamate (11e)

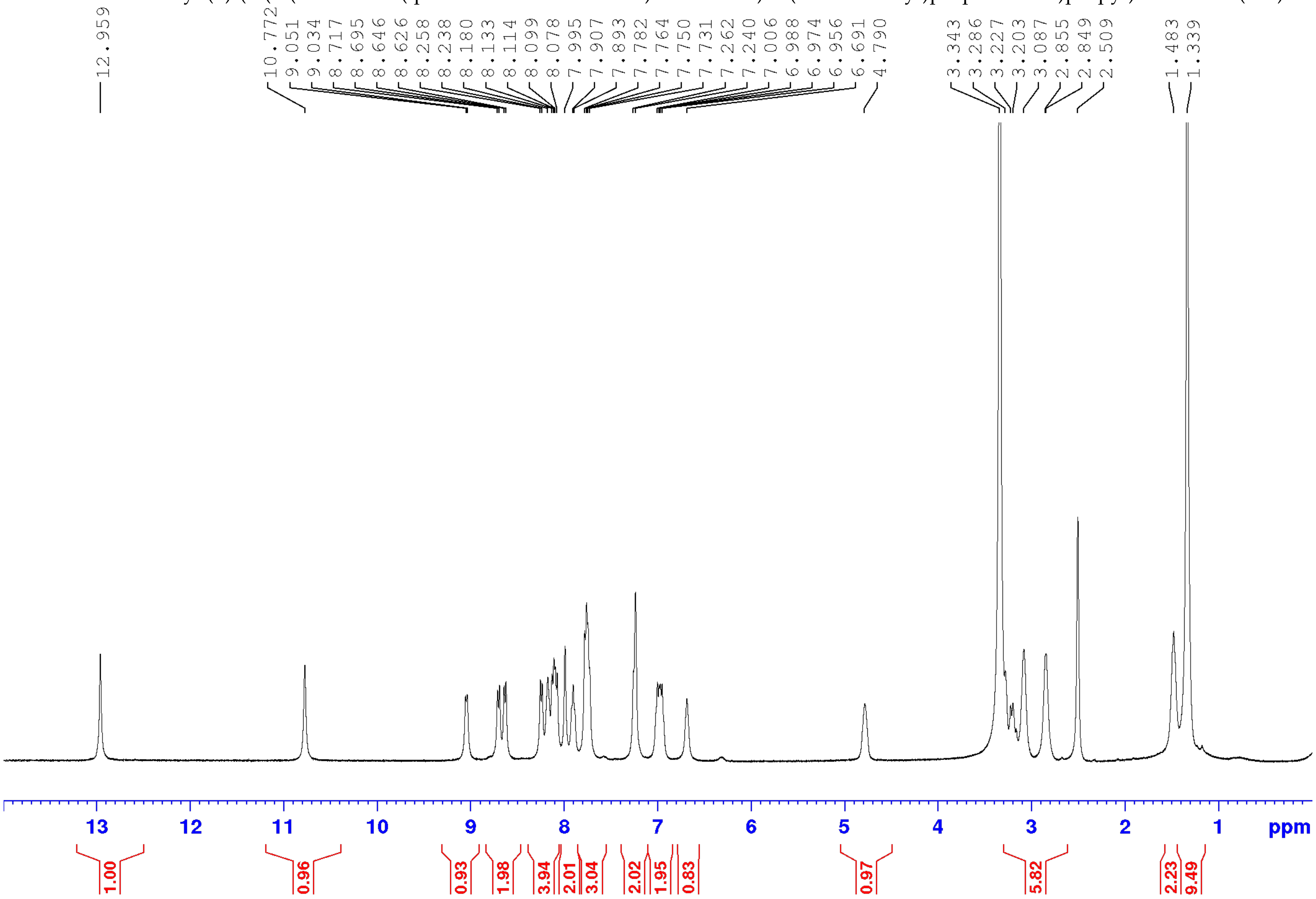

^{13}C NMR of *tert*-butyl (S)-(3-(2-(5-bromo-2-(quinoline-2-carboxamido)benzamido)-3-(1*H*-indol-3-yl)propanamido)propyl)carbamate (11e)

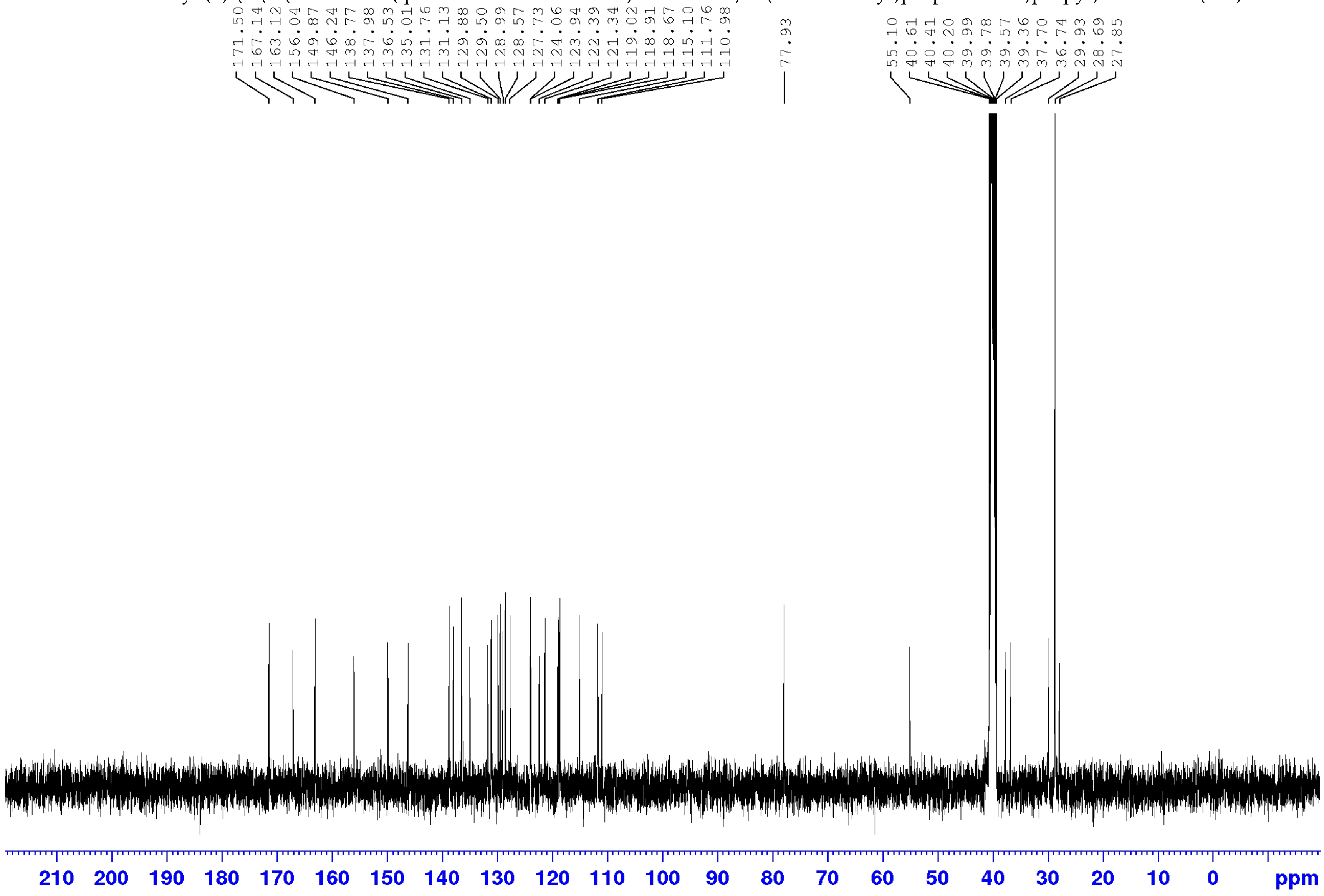

^{1}H NMR of (S)-N-(1-((2-aminoethyl)amino)-3-(1H-indol-3-yl)-1-oxopropan-2-yl)-5-bromo-2-(2-(naphthalen-1-yl)acetamido)benzamide (12b)

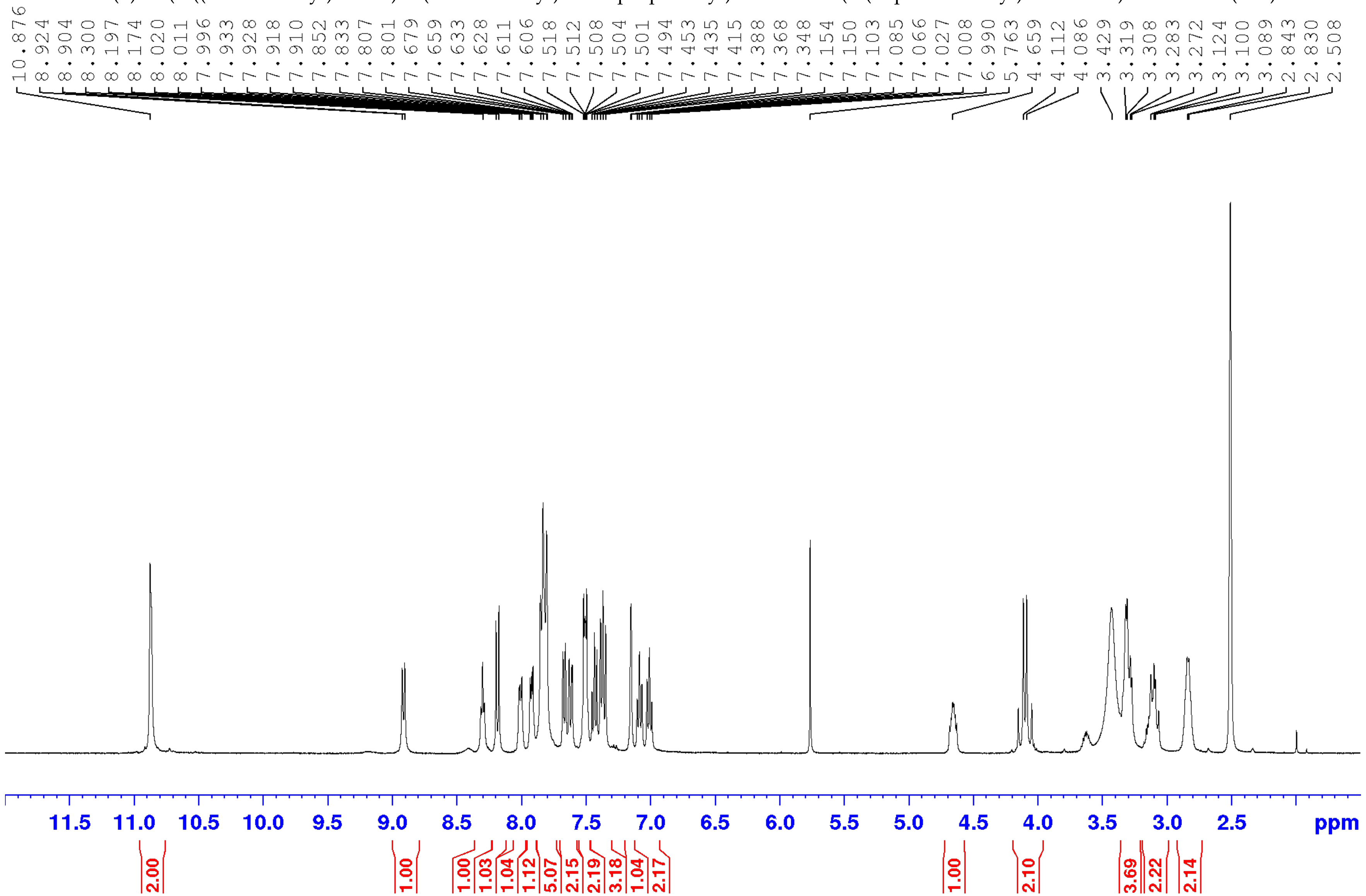

13C NMR of (S)-N-(1-((2-aminoethyl)amino)-3-(1H-indol-3-yl)-1-oxopropan-2-yl)-5-bromo-2-(2-(naphthalen-1-yl)acetamido)benzamide (12b)

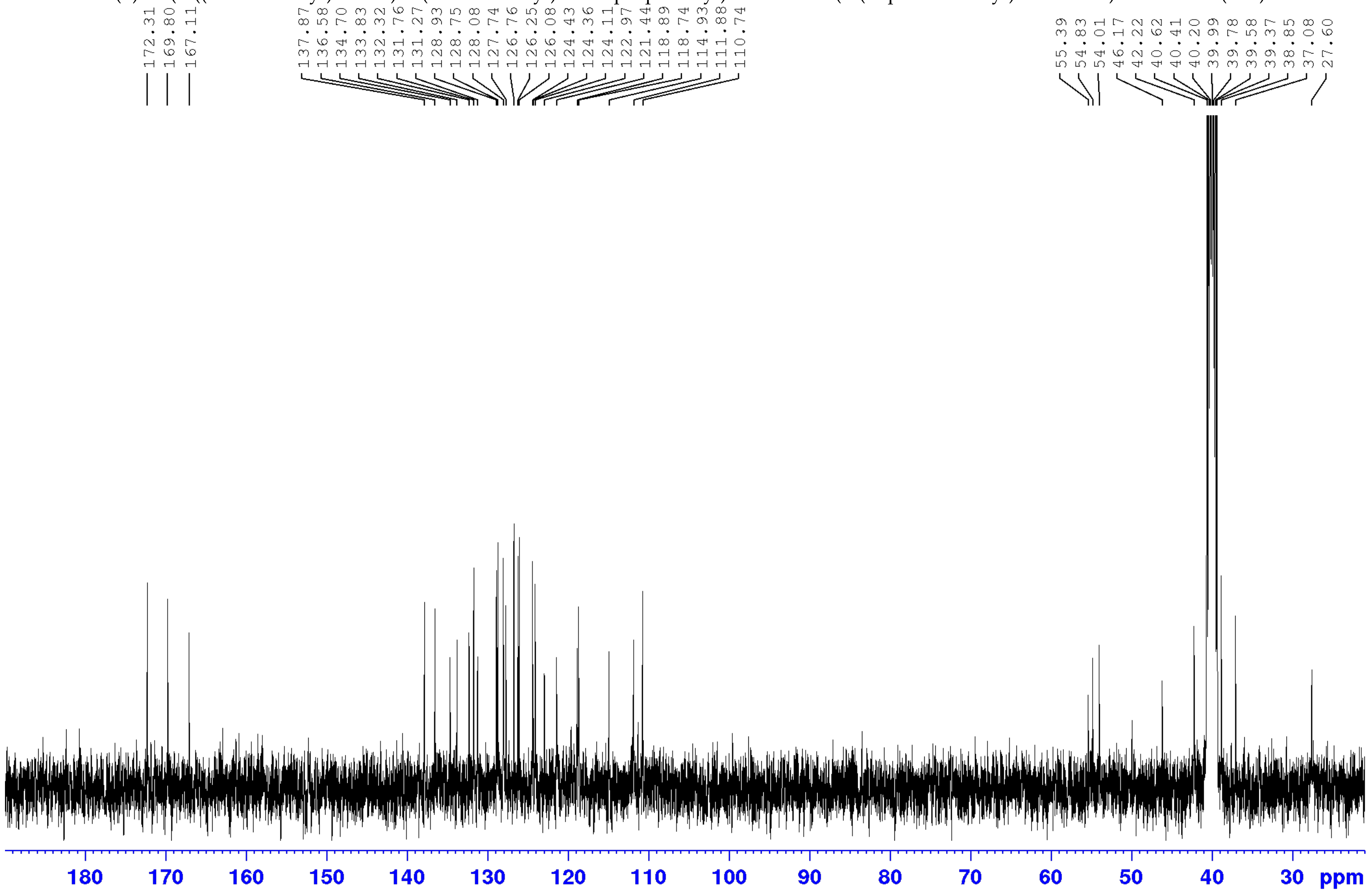

^{1}H NMR of (S)-*N*-(2-((1-((2-aminoethyl)amino)-3-(1*H*-indol-3-yl)-1-oxopropan-2-yl)carbamoyl)-4-bromophenyl)-2-methoxy-1-naphthamide (12c)

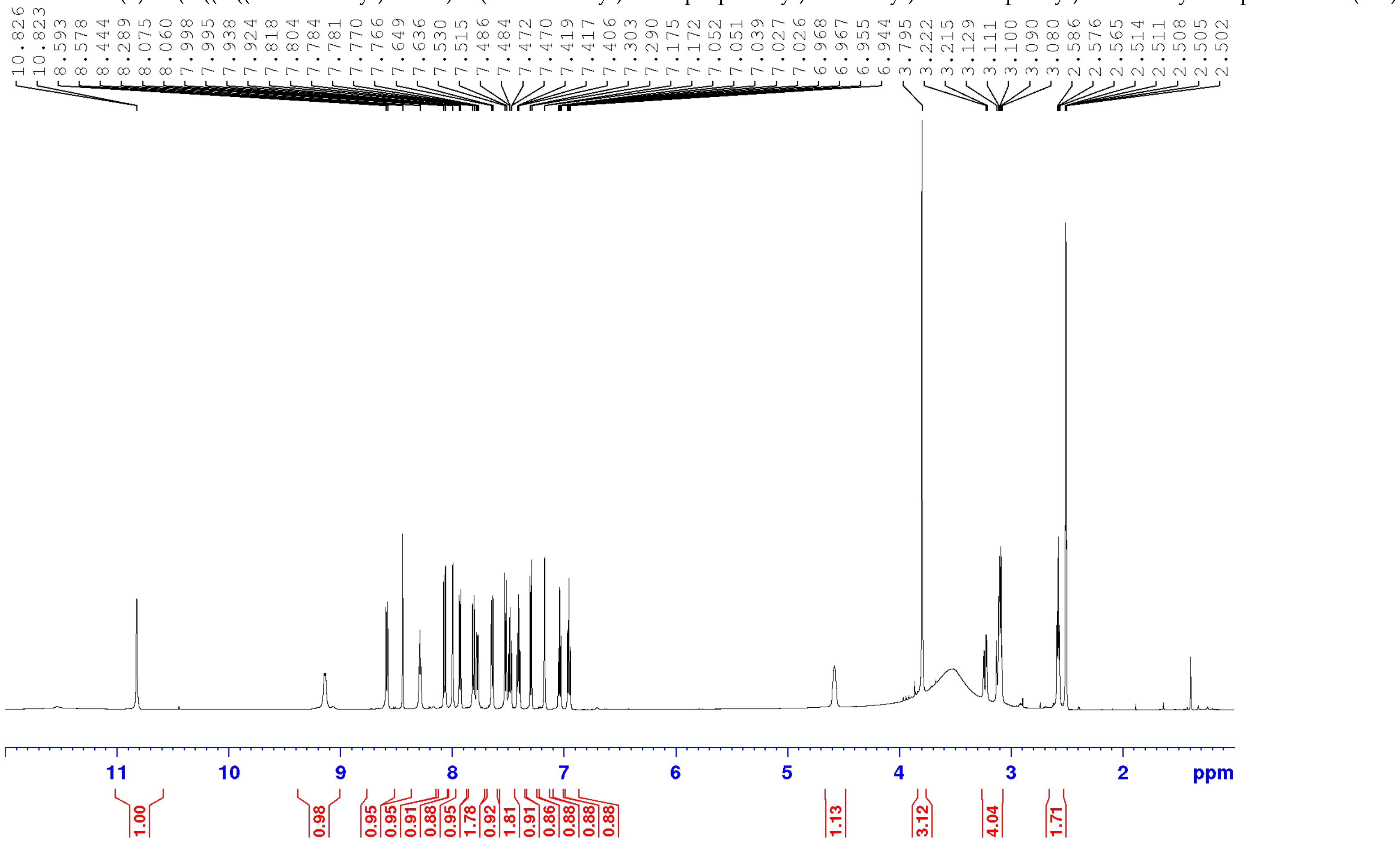

^{13}C NMR of (S)-*N*-(2-((1-((2-aminoethyl)amino)-3-(1*H*-indol-3-yl)-1-oxopropan-2-yl)carbamoyl)-4-bromophenyl)-2-methoxy-1-naphthamide (12c)

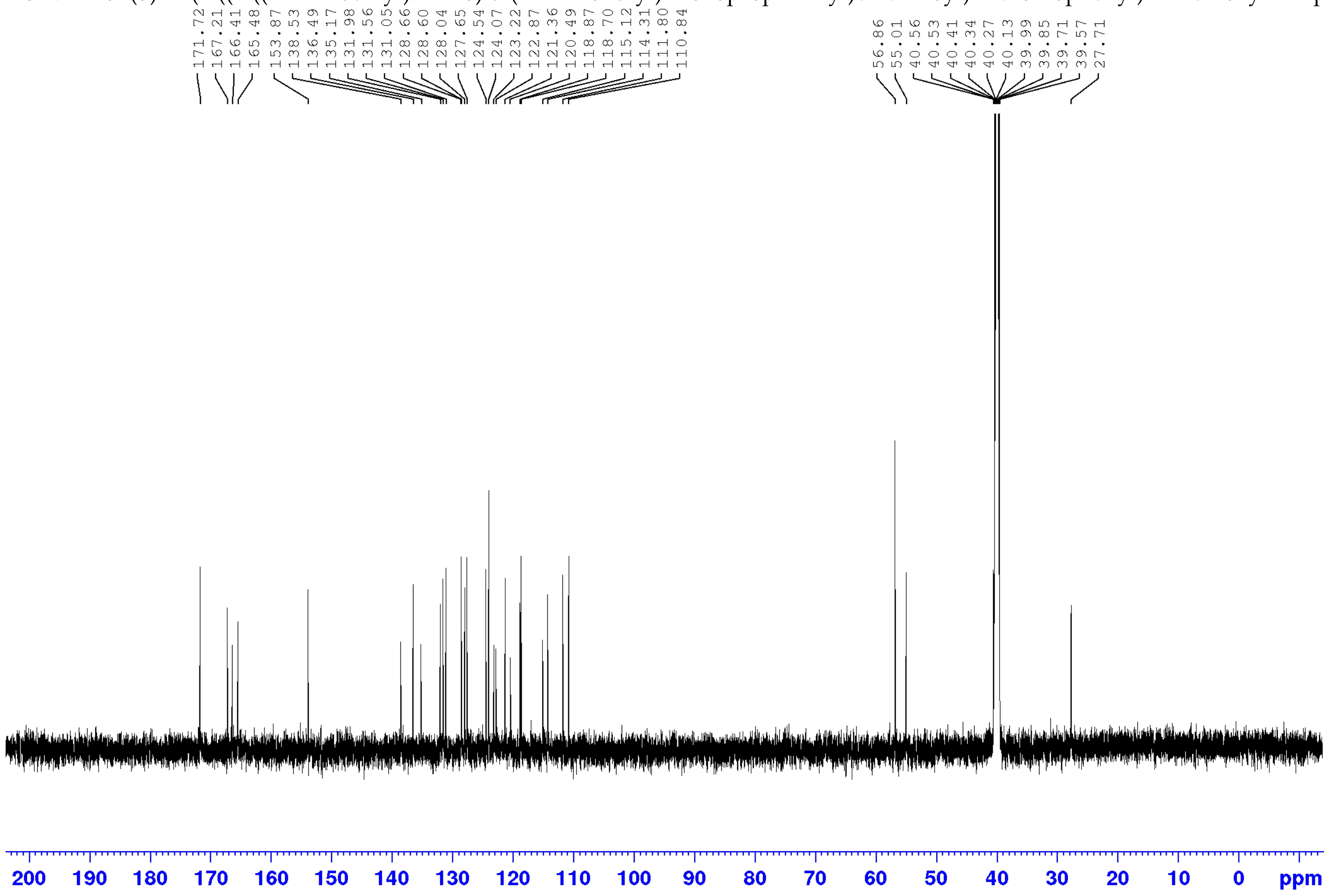

^{1}H NMR of (S)-*N*-(2-((1-((2-aminoethyl)amino)-3-(1*H*-indol-3-yl)-1-oxopropan-2-yl)carbamoyl)-4-bromophenyl)-3-methoxy-2-naphthamide (12d)

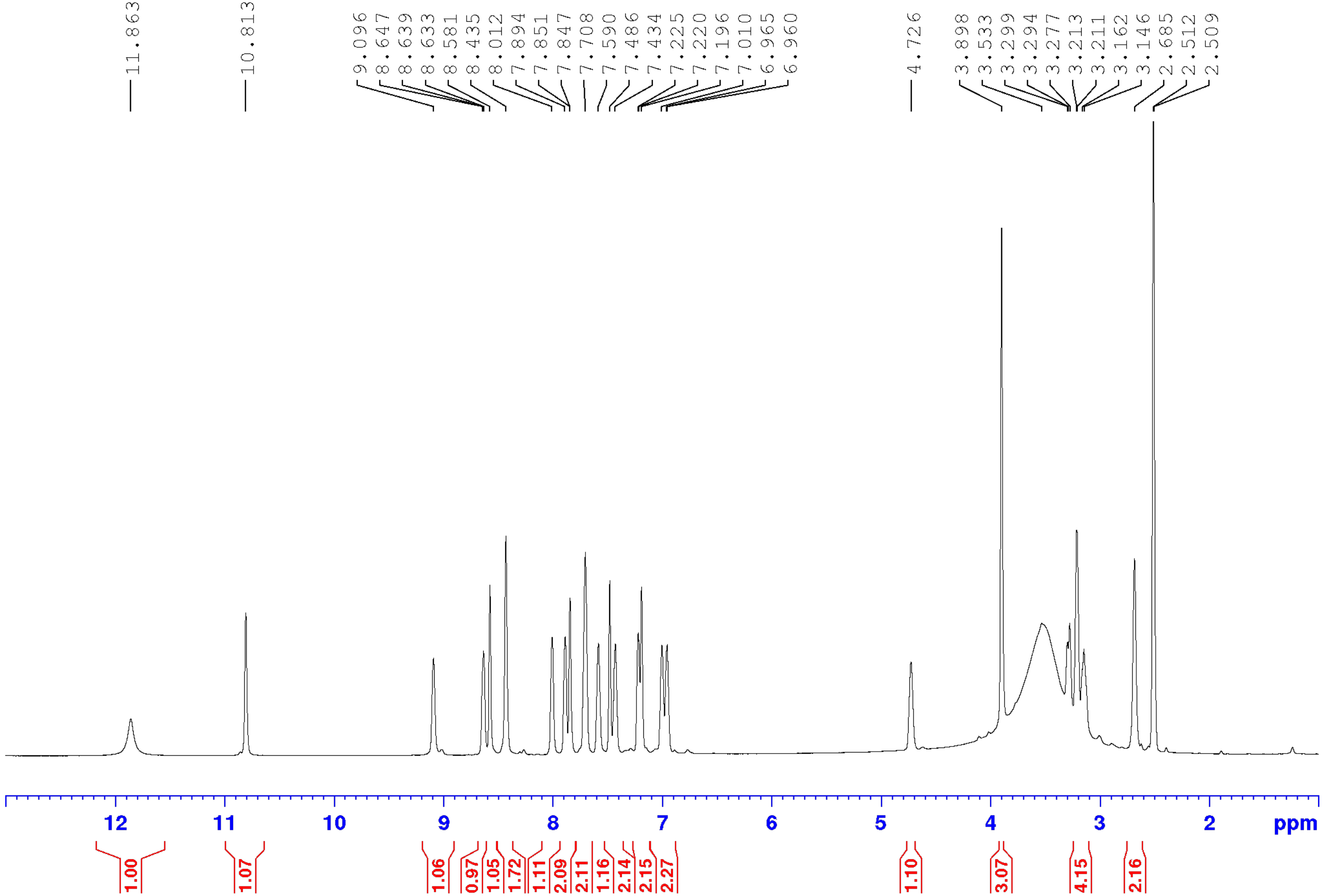

^{1}H NMR of (*S*)-N-(2-((1-((2-aminoethyl)amino)-3-(1*H*-indol-3-yl)-1-oxopropan-2-yl)carbamoyl)-4-bromophenyl)quinoline-2-carboxamide (12e)

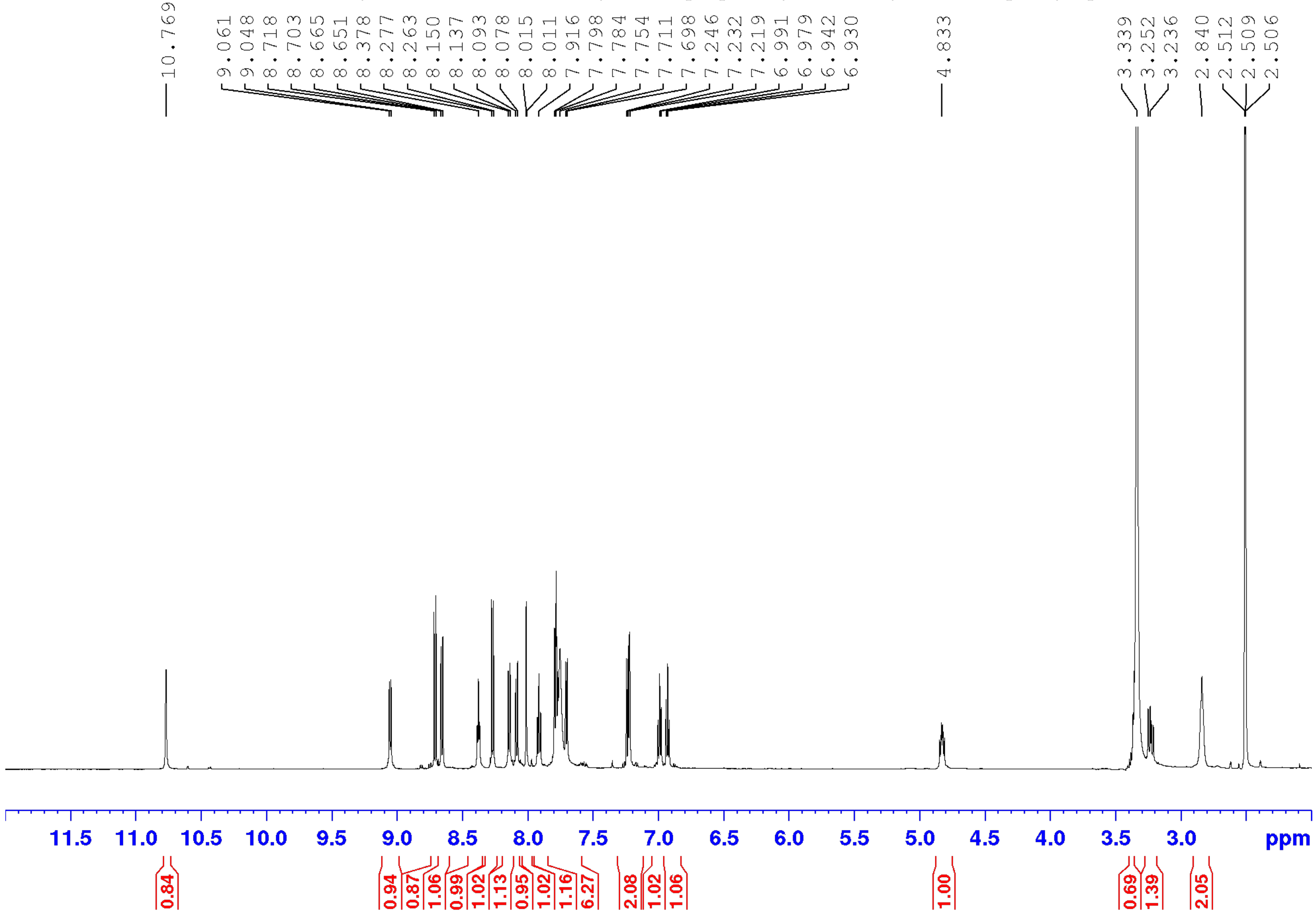

^{1}H NMR of (*S*)-N-(2-((1-((2-aminoethyl)amino)-3-(1*H*-indol-3-yl)-1-oxopropan-2-yl)carbamoyl)-4-bromophenyl)quinoline-2-carboxamide (12e)

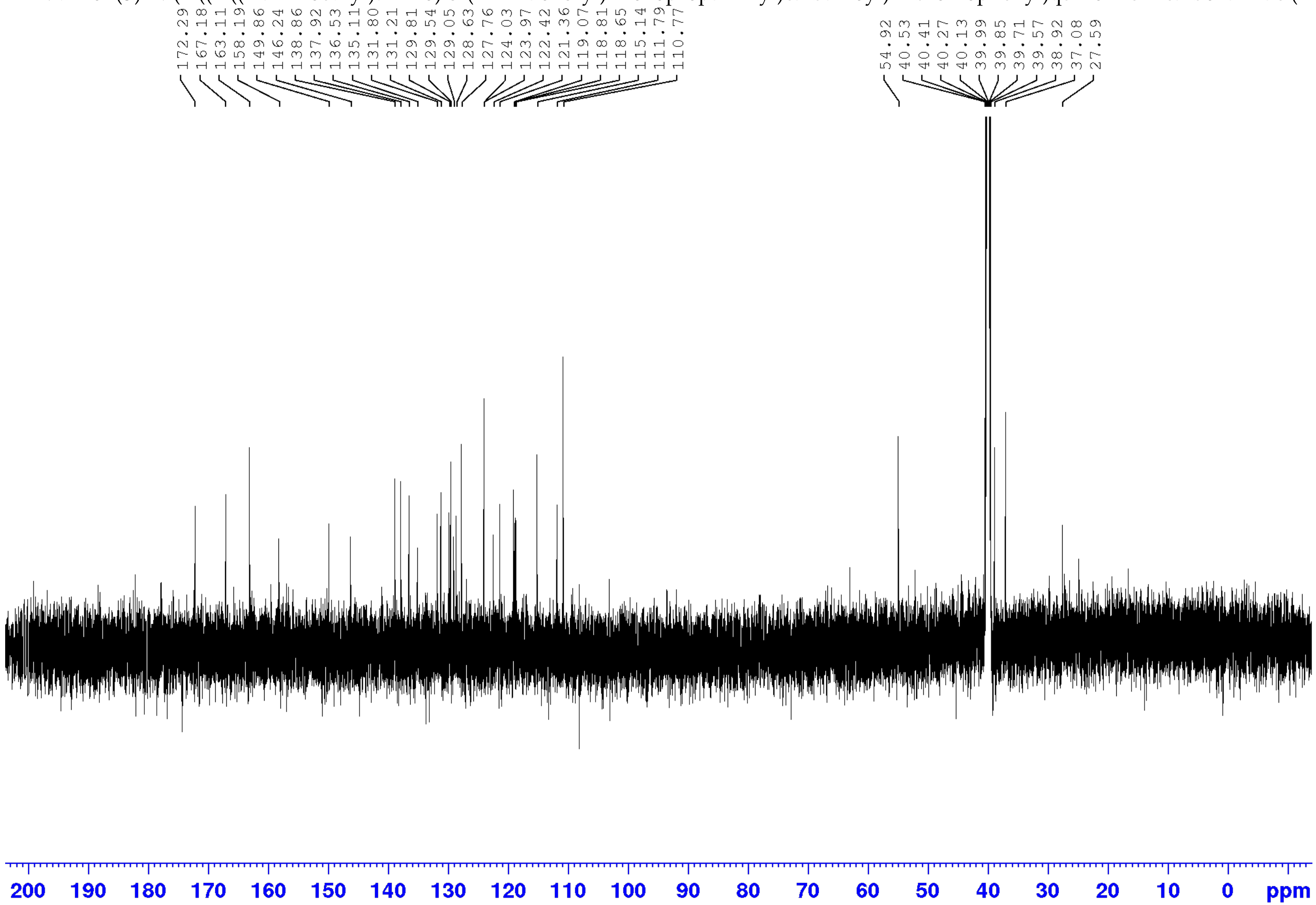

^{1}H NMR of (*S*)-N-(2-((1-((2-aminoethyl)amino)-3-(1*H*-indol-3-yl)-1-oxopropan-2-yl)carbamoyl)-4-bromophenyl)-1H-indole-2-carboxamide (12f)

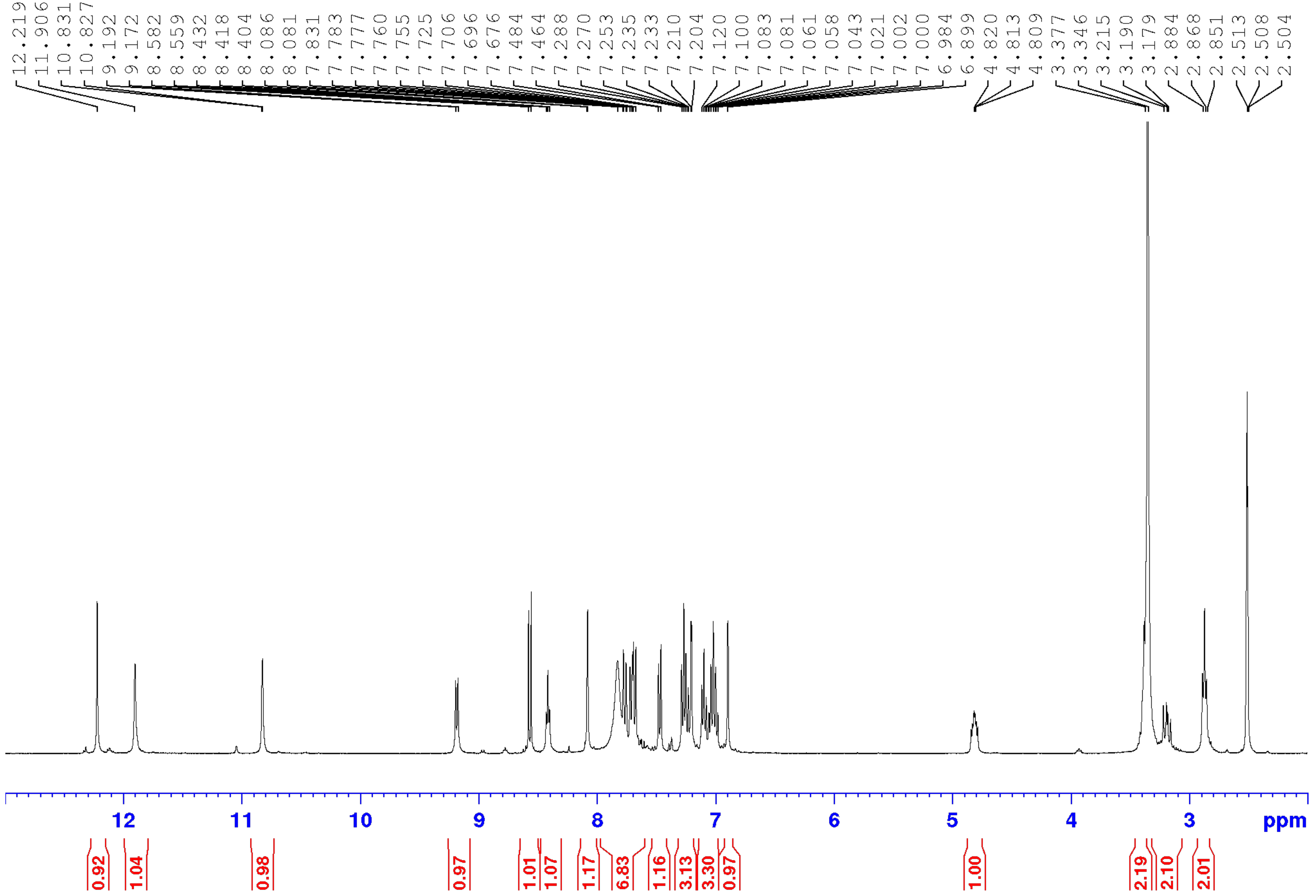

^{13}C NMR of (*S*)-N-(2-((1-((2-aminoethyl)amino)-3-(1*H*-indol-3-yl)-1-oxopropan-2-yl)carbamoyl)-4-bromophenyl)-1H-indole-2-carboxamide (12f)

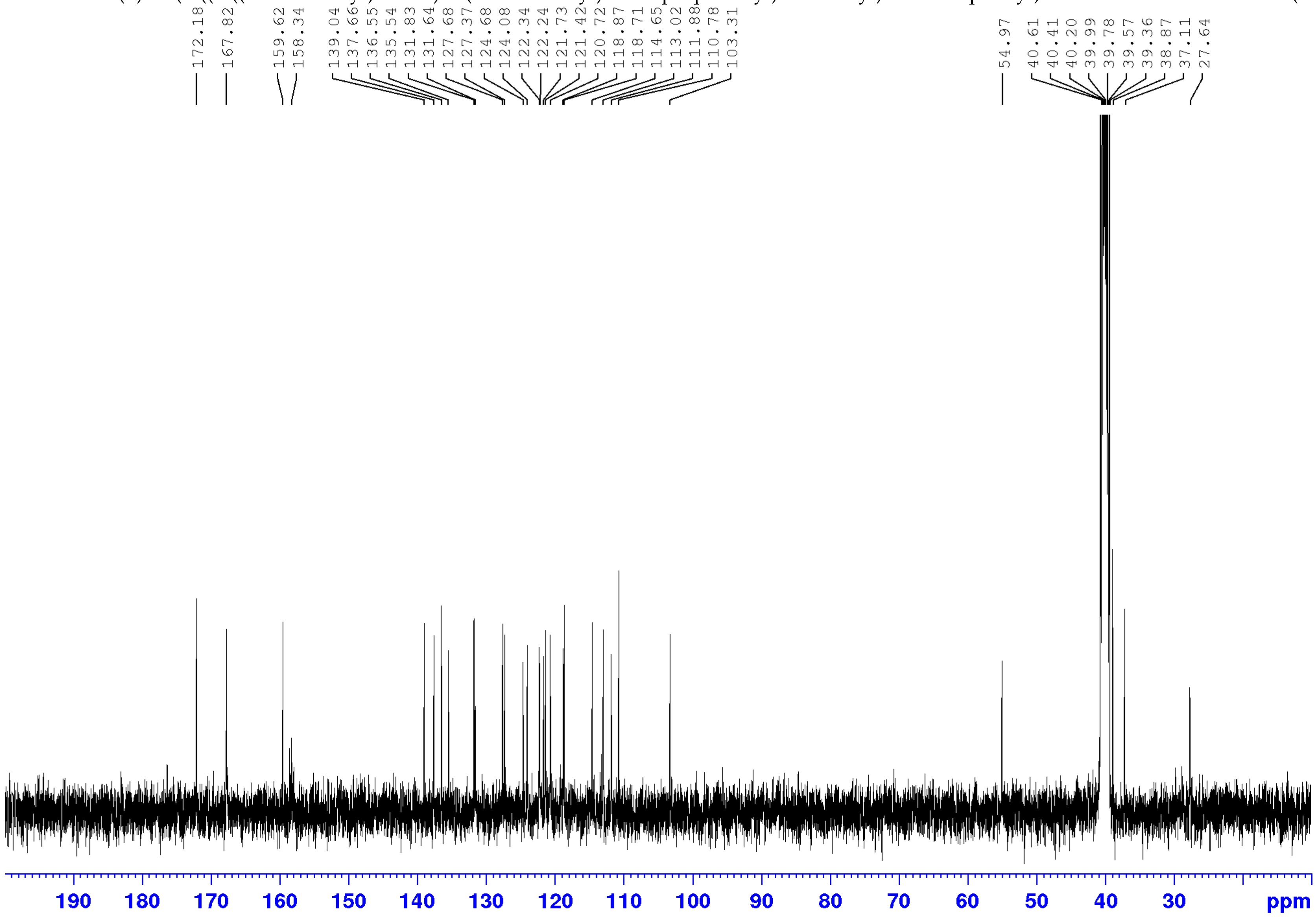

^{1}H NMR of (*S*)-N-(2-((1-((2-aminoethyl)amino)-3-(1*H*-indol-3-yl)-1-oxopropan-2-yl)carbamoyl)-4-bromophenyl)thiophene-2-carboxamide (12g)

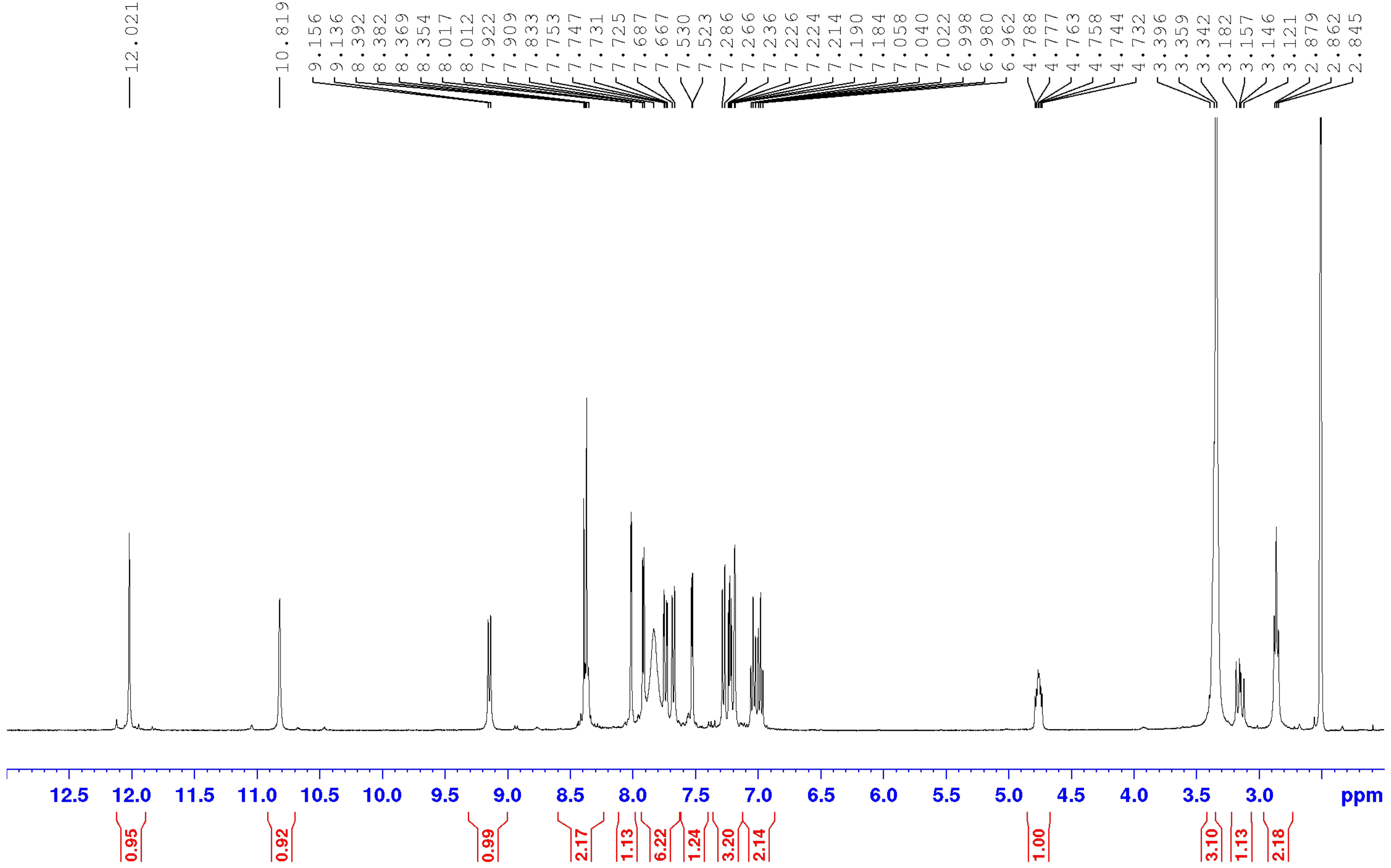

^{13}C NMR of (*S*)-N-(2-((1-((2-aminoethyl)amino)-3-(1*H*-indol-3-yl)-1-oxopropan-2-yl)carbamoyl)-4-bromophenyl)thiophene-2-carboxamide (12g)

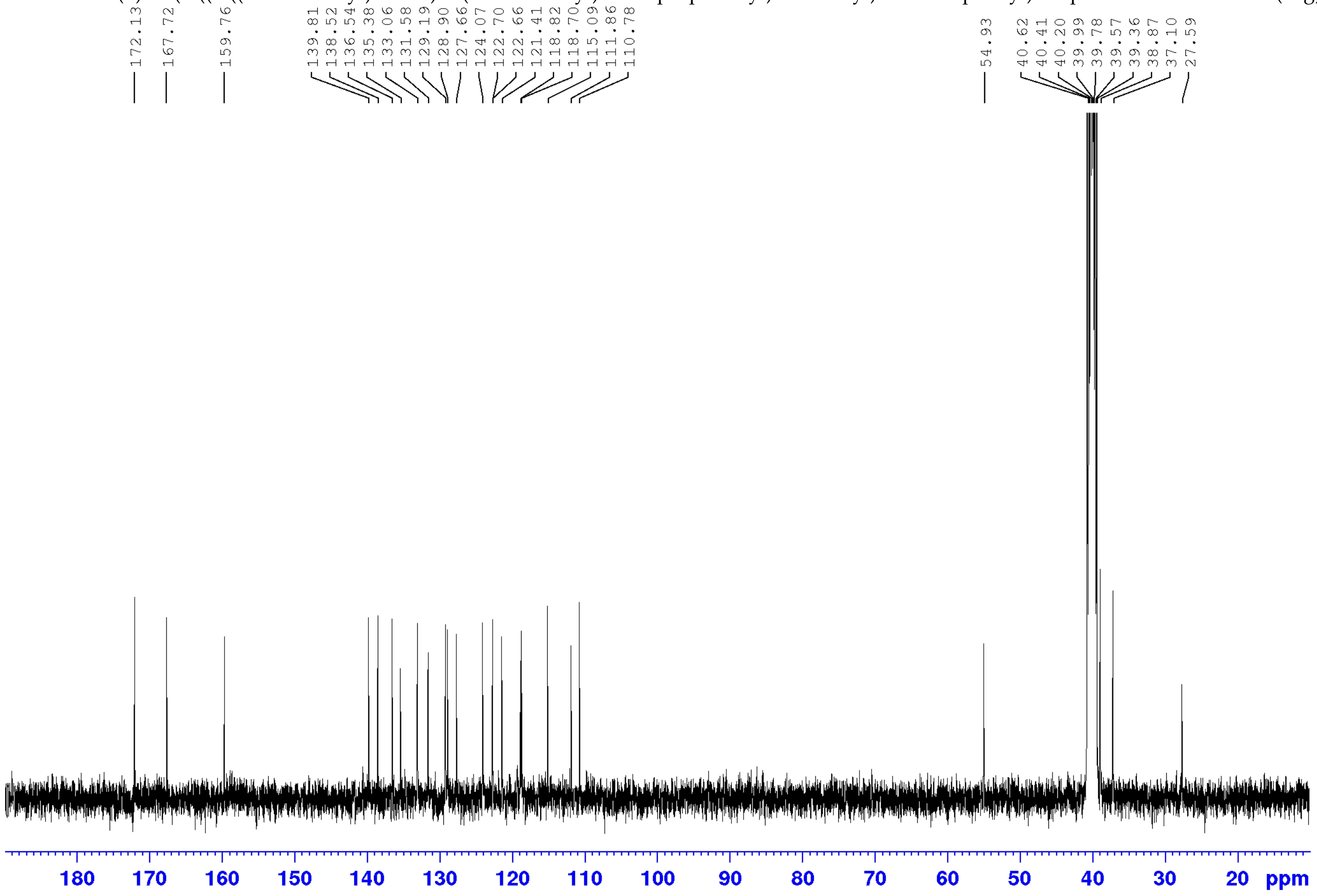

^{1}H NMR of (S)-N-(2-((1-((2-aminoethyl)amino)-3-(1H-indol-3-yl)-1-oxopropan-2-yl)carbamoyl)-4-bromophenyl)thiophene-3-carboxamide (12h)

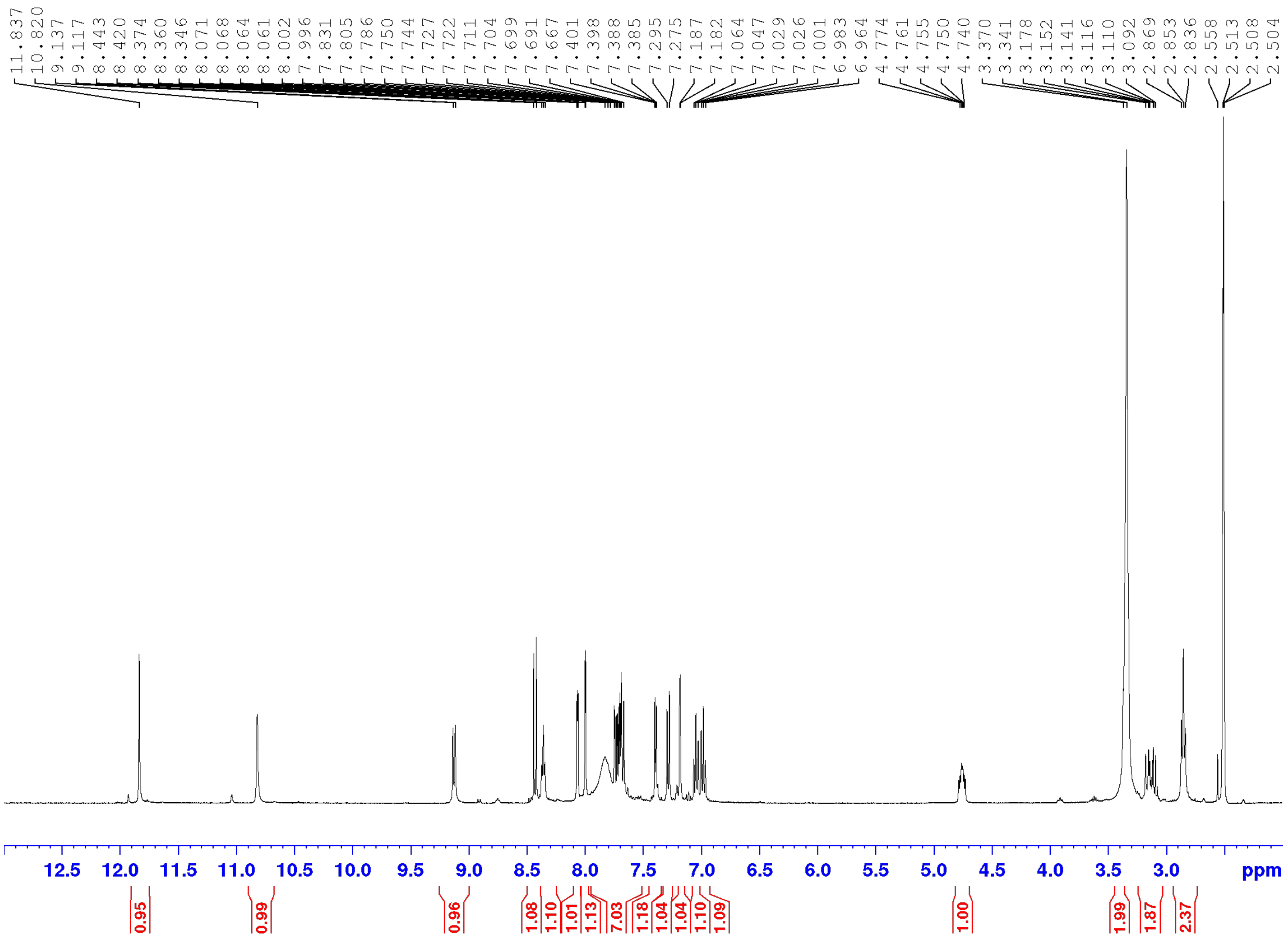

^{13}C NMR of (S)-N-(2-((1-((2-aminoethyl)amino)-3-(1H-indol-3-yl)-1-oxopropan-2-yl)carbamoyl)-4-bromophenyl)thiophene-3-carboxamide (12h)

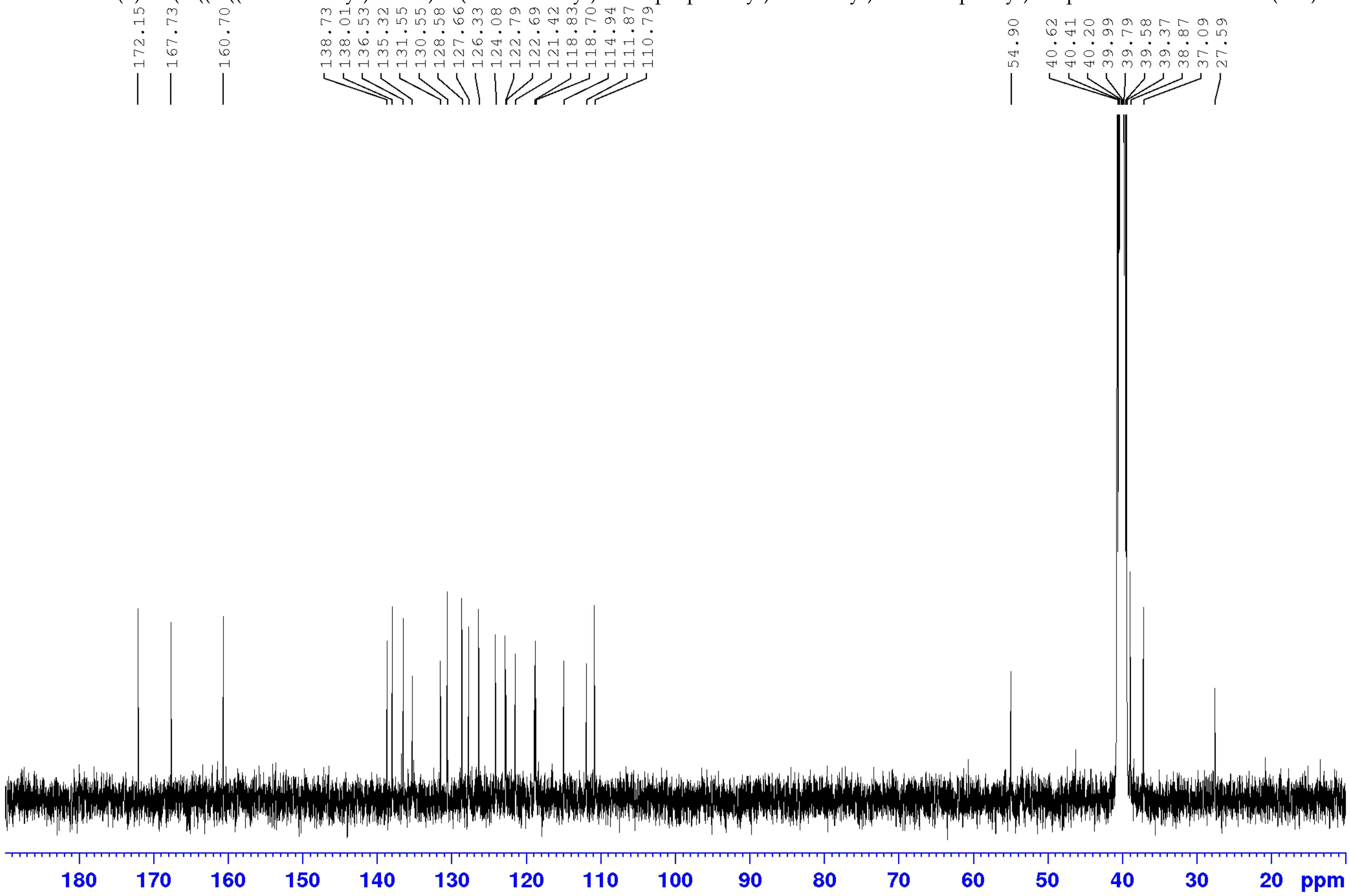

^{1}H NMR of (S)-*N*-(2-((1-((2-aminoethyl)amino)-3-(1H-indol-3-yl)-1-oxopropan-2-yl)carbamoyl)-4-bromophenyl)-[1,1'-biphenyl]-2-carboxamide (12i)

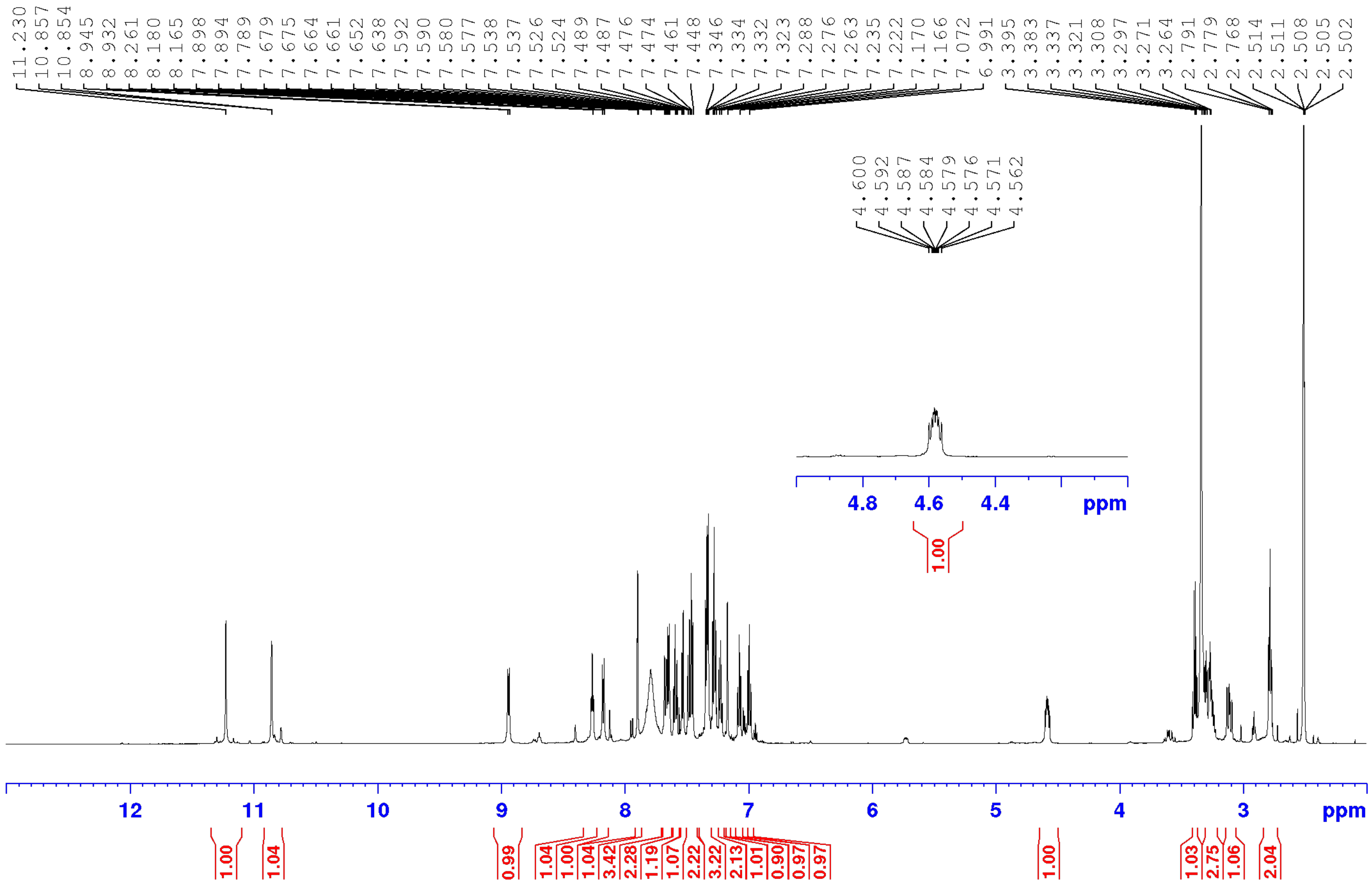

^{13}C NMR of (S)-*N*-(2-((1-((2-aminoethyl)amino)-3-(1H-indol-3-yl)-1-oxopropan-2-yl)carbamoyl)-4-bromophenyl)-[1,1'-biphenyl]-2-carboxamide (12i)

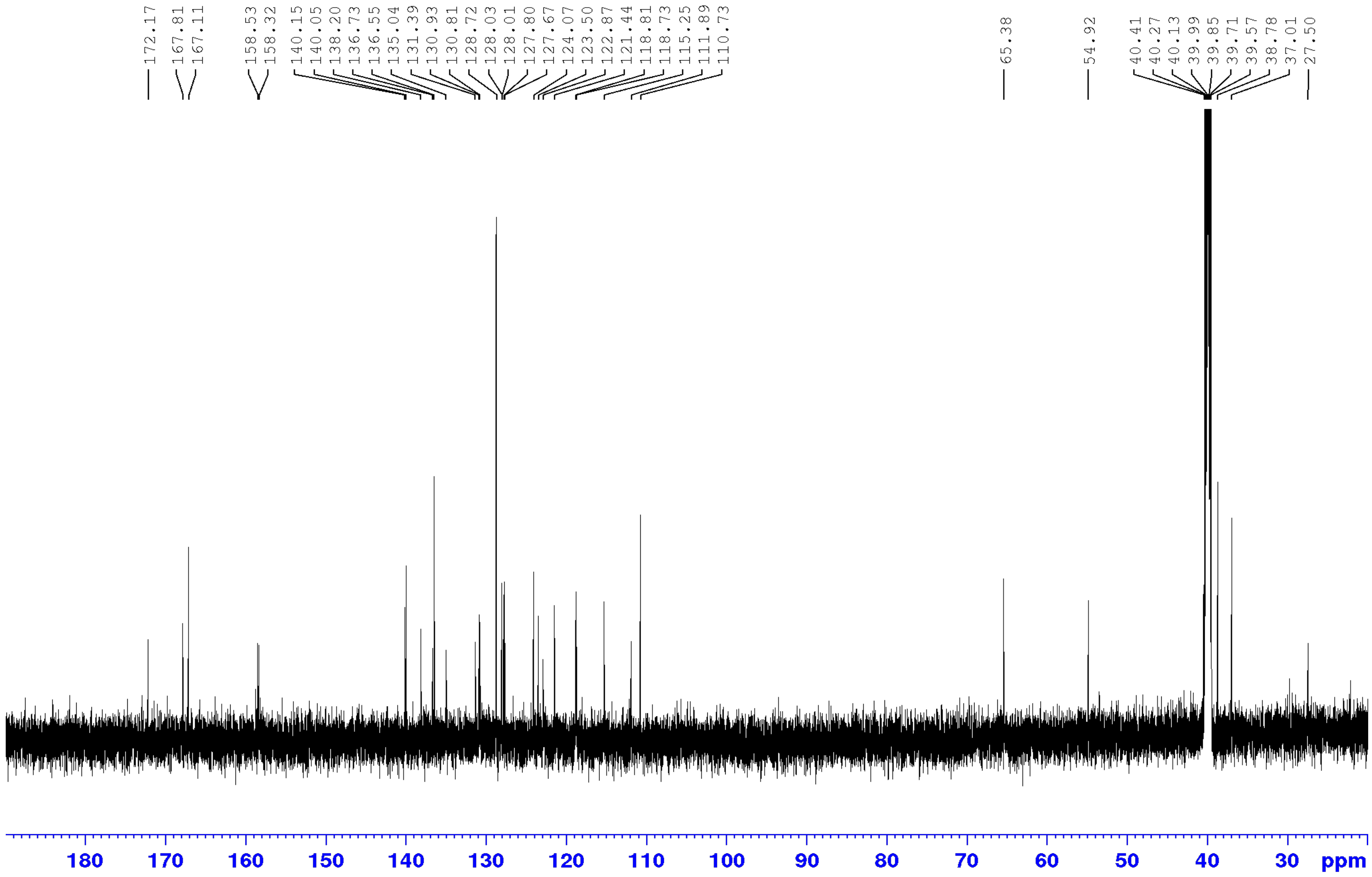

1H NMR of (*S*)-N-(2-((1-((2-aminoethyl)amino)-3-(1*H*-indol-3-yl)-1-oxopropan-2-yl)carbamoyl)-4-bromophenyl)-[1,1'-biphenyl]-3-carboxamide (12j)

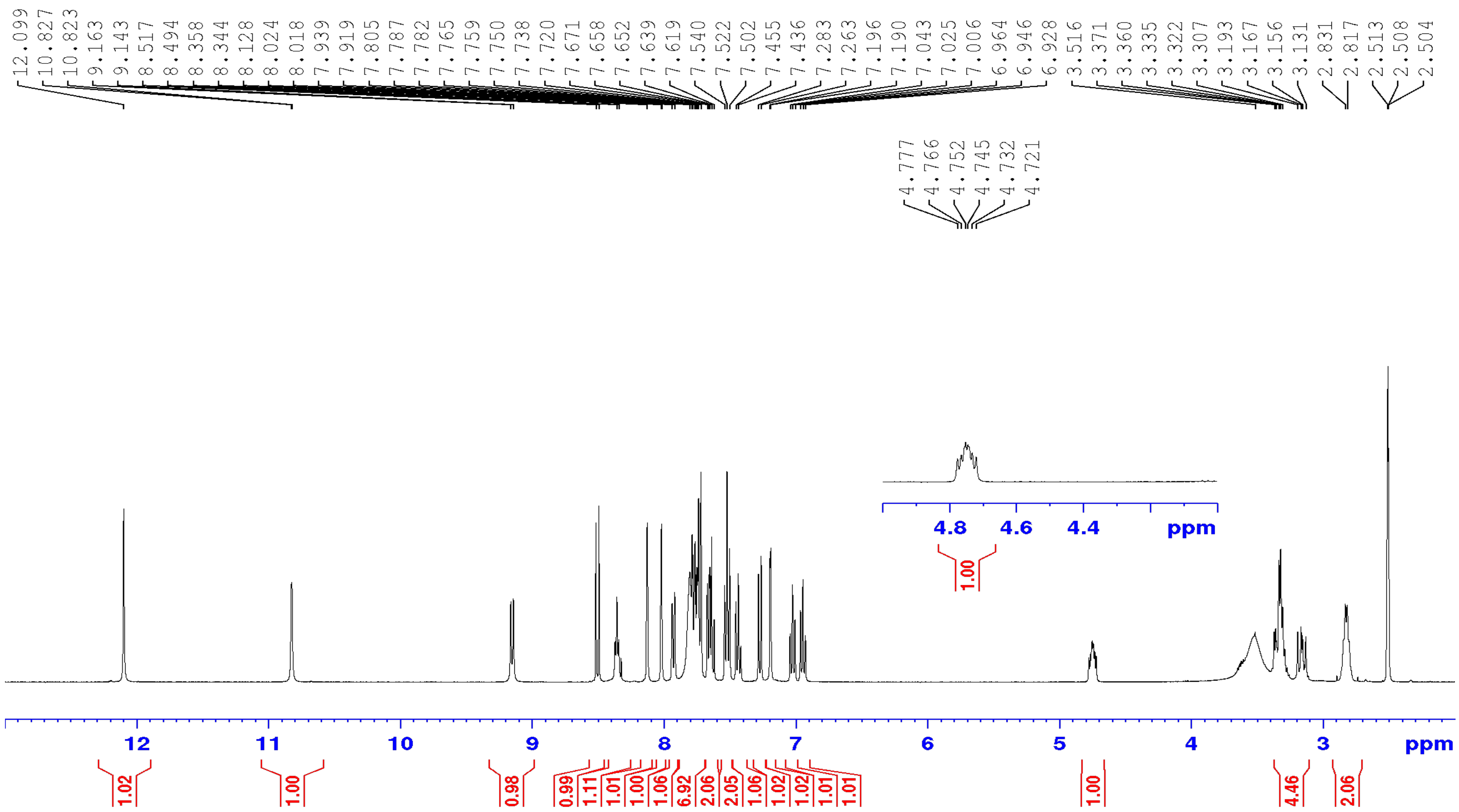

^{13}C NMR of (*S*)-N-(2-((1-((2-aminoethyl)amino)-3-(1*H*-indol-3-yl)-1-oxopropan-2-yl)carbamoyl)-4-bromophenyl)-[1,1'-biphenyl]-3-carboxamide (12j)

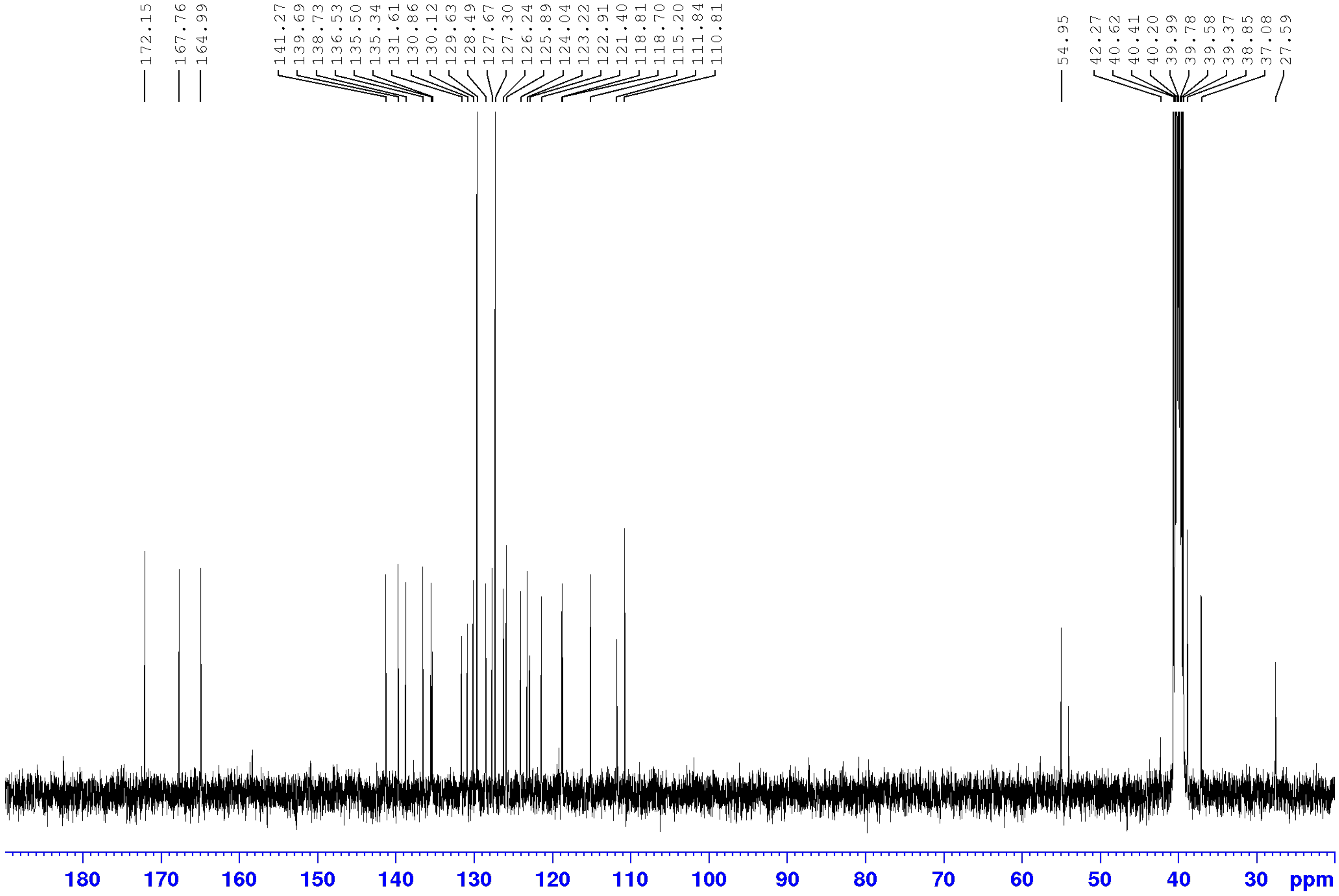

^{1}H NMR of (*S*)-N-(2-((1-((2-aminoethyl)amino)-3-(1*H*-indol-3-yl)-1-oxopropan-2-yl)carbamoyl)-4-bromophenyl)-[1,1'-biphenyl]-4-carboxamide (12k)

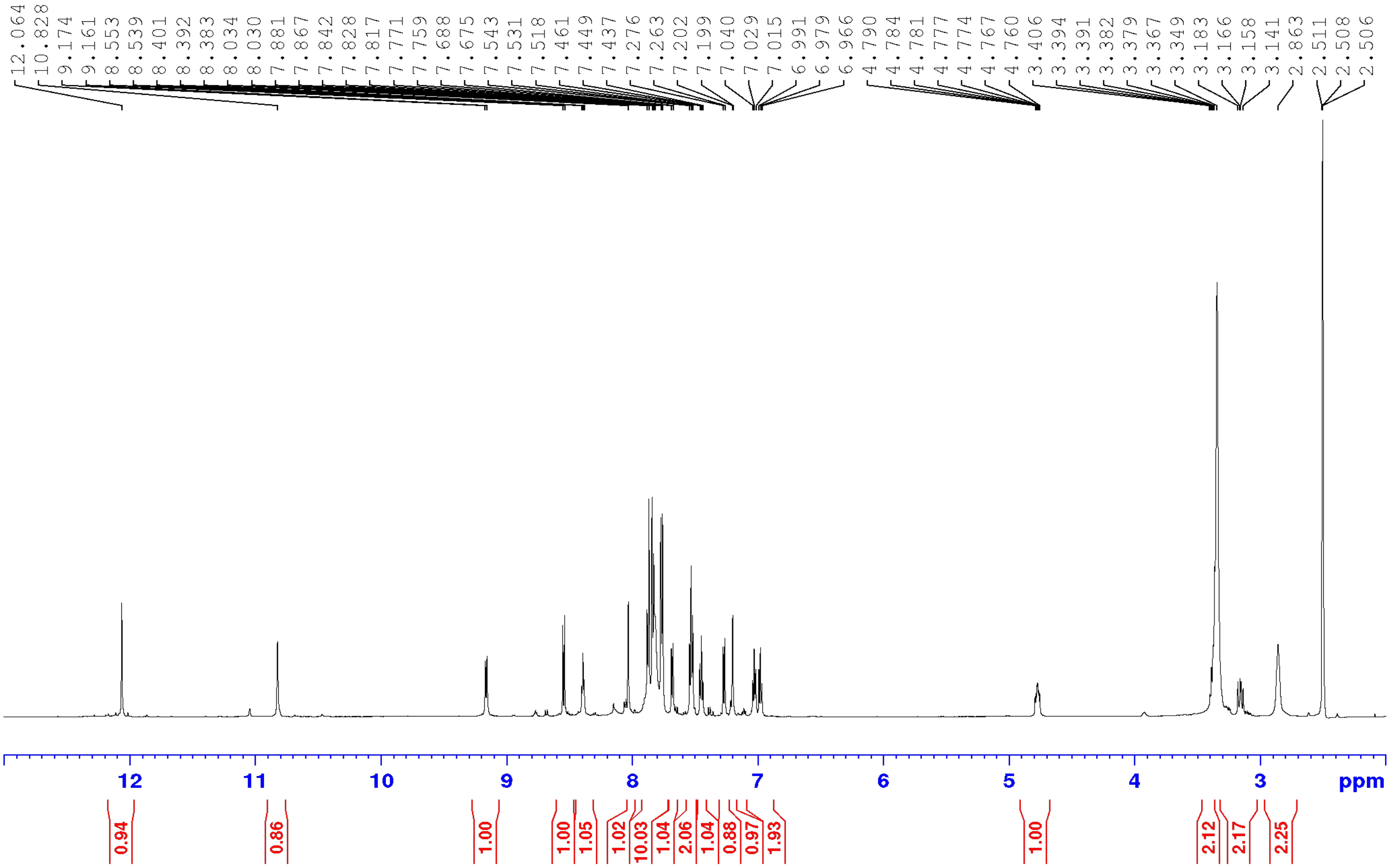

^{13}C NMR of (*S*)-N-(2-((1-((2-aminoethyl)amino)-3-(1*H*-indol-3-yl)-1-oxopropan-2-yl)carbamoyl)-4-bromophenyl)-[1,1'-biphenyl]-4-carboxamide (12k)

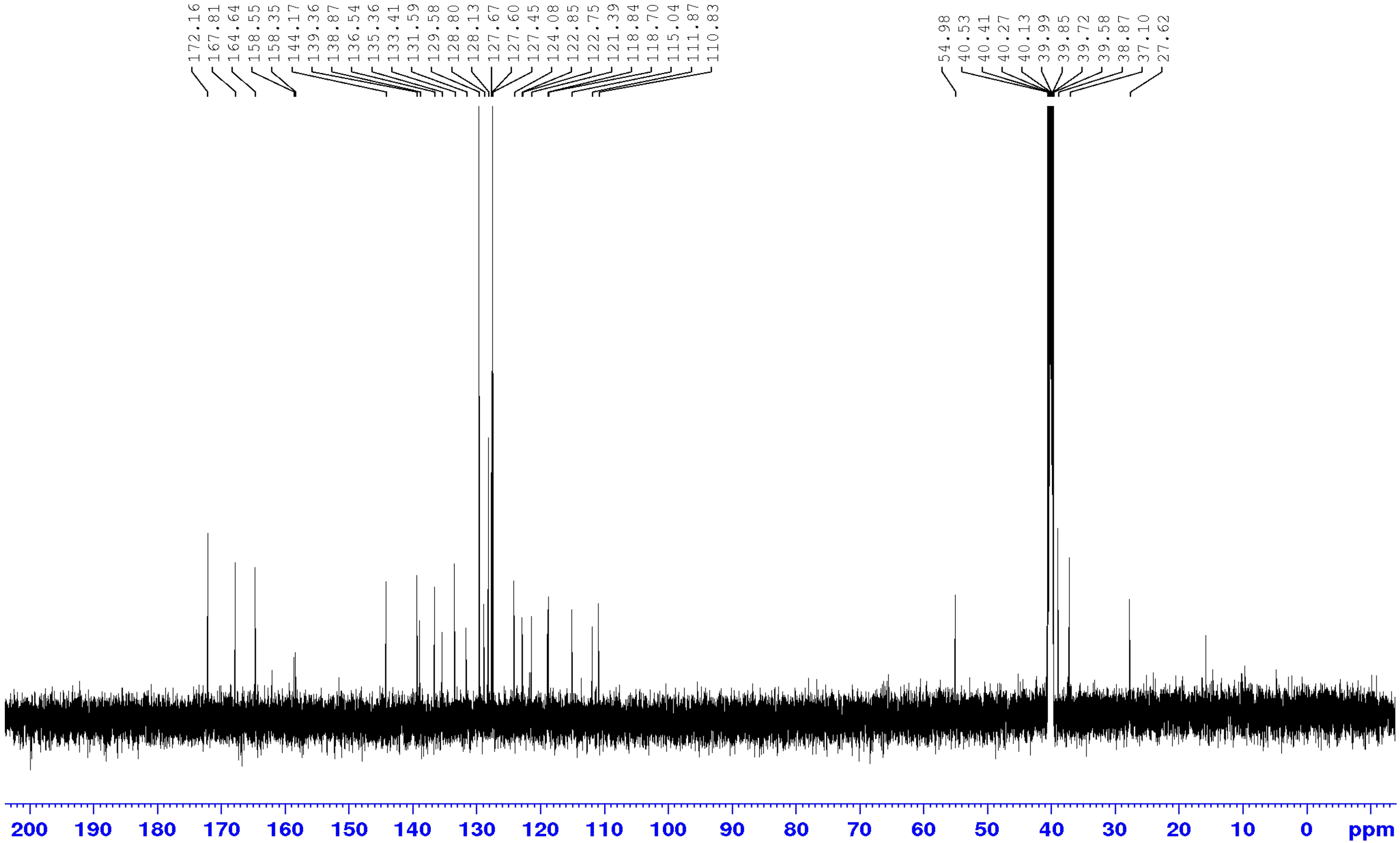

^{1}H NMR of (S)-*N*-(1-((3-aminopropyl)amino)-3-(1*H*-indol-3-yl)-1-oxopropan-2-yl)-5-bromo-2-(2-(naphthalen-1-yl)acetamido)benzamide (13b)

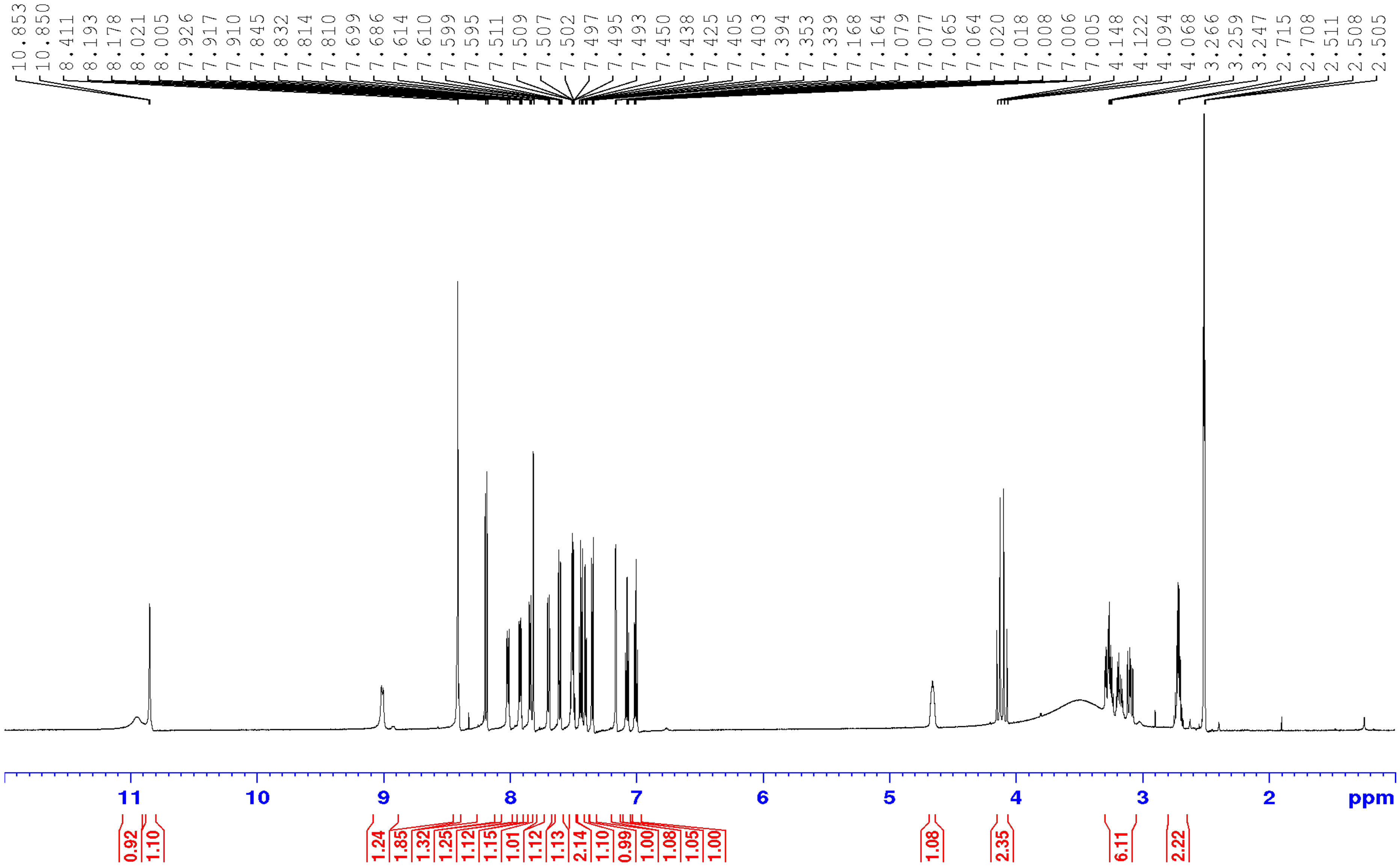

^{13}C NMR of (S)-*N*-(1-((3-aminopropyl)amino)-3-(1*H*-indol-3-yl)-1-oxopropan-2-yl)-5-bromo-2-(2-(naphthalen-1-yl)acetamido)benzamide (13b)

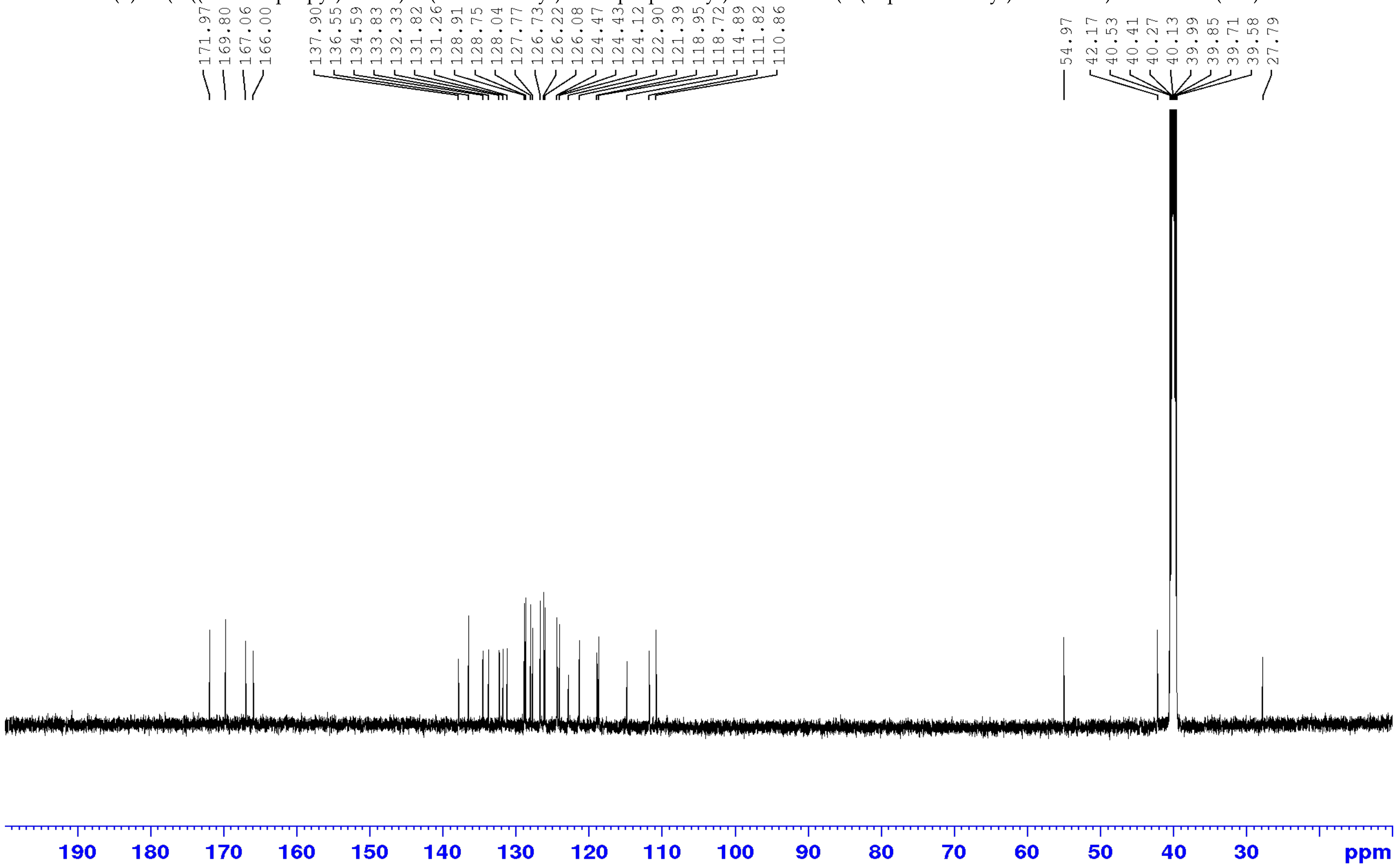

^{1}H NMR of (S)-N-(2-((1-((3-aminopropyl)amino)-3-(1*H*-indol-3-yl)-1-oxopropan-2-yl)carbamoyl)-4-bromophenyl)-2-methoxy-1-naphthamide (13c)

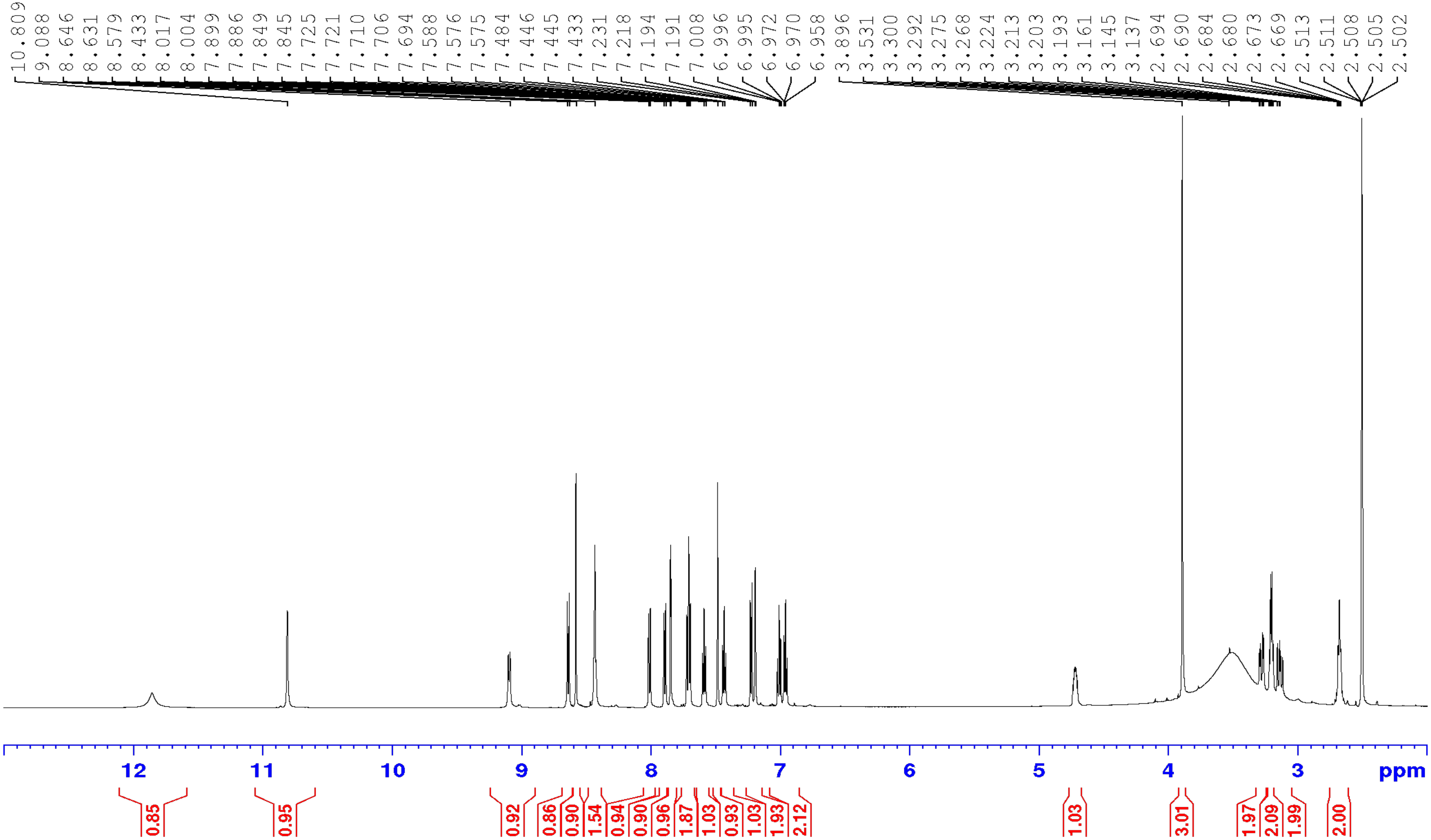

^{13}C NMR of (S)-N-(2-((1-((3-aminopropyl)amino)-3-(1*H*-indol-3-yl)-1-oxopropan-2-yl)carbamoyl)-4-bromophenyl)-2-methoxy-1-naphthamide (13c)

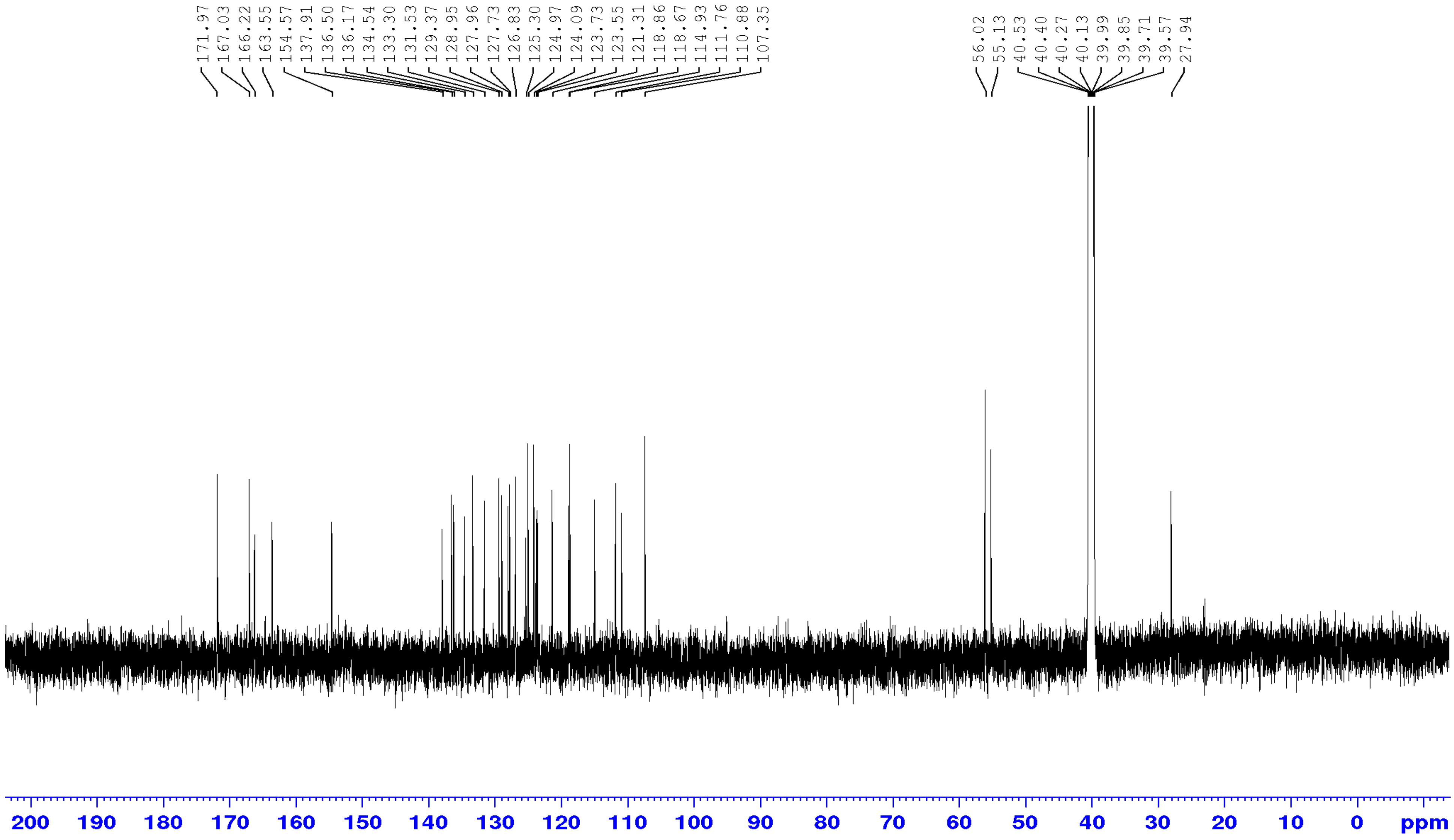

^{1}H NMR of (S)-N-(2-((1-((3-aminopropyl)amino)-3-(1*H*-indol-3-yl)-1-oxopropan-2-yl)carbamoyl)-4-bromophenyl)-3-methoxy-2-naphthamide (13d)

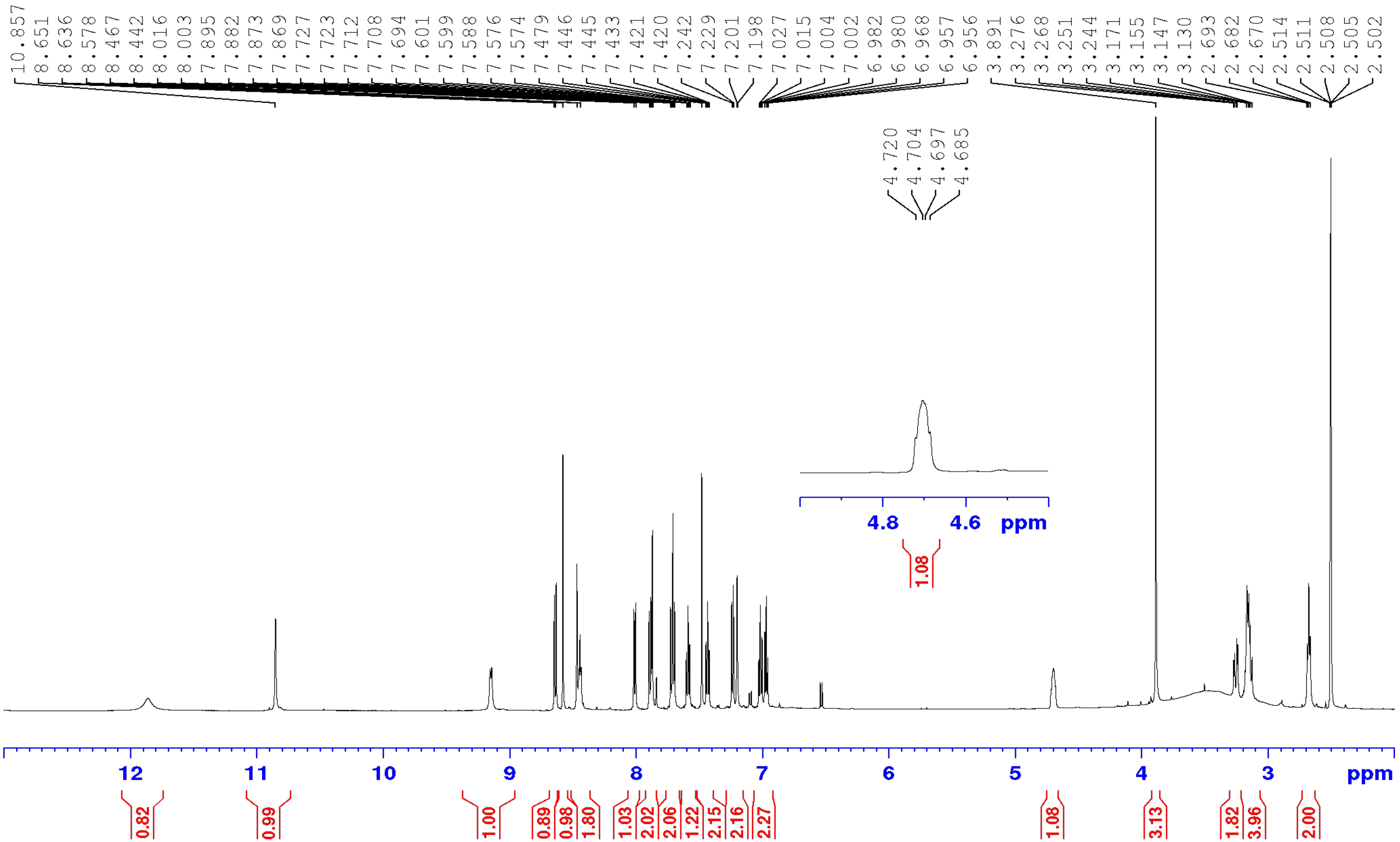

^{13}C NMR of (S)-N-(2-((1-((3-aminopropyl)amino)-3-(1*H*-indol-3-yl)-1-oxopropan-2-yl)carbamoyl)-4-bromophenyl)-3-methoxy-2-naphthamide (13d)

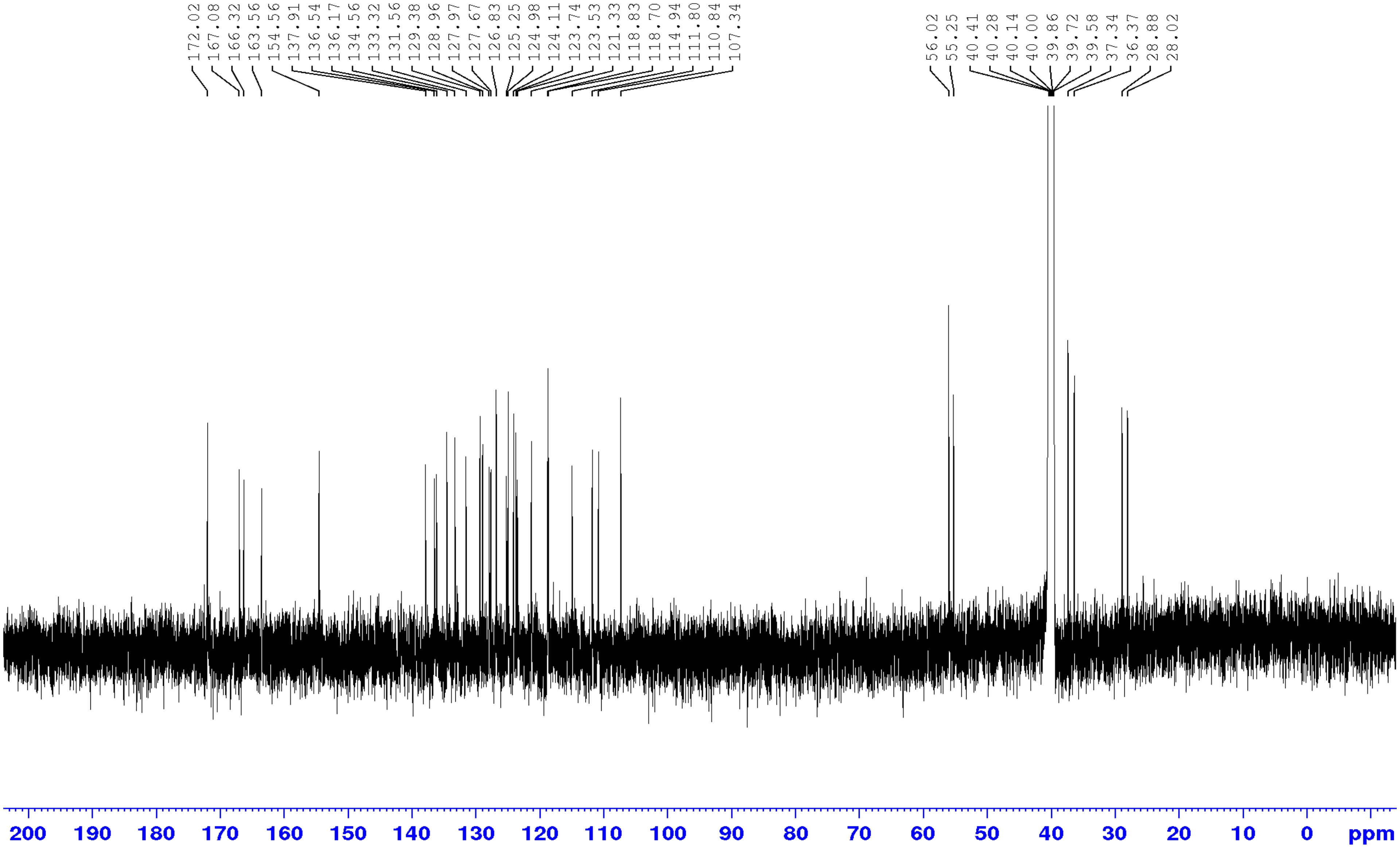

^{1}H NMR of (S)-N-(2-((1-((3-aminopropyl)amino)-3-(1*H*-indol-3-yl)-1-oxopropan-2-yl)carbamoyl)-4-bromophenyl)quinoline-2-carboxamide (13e)

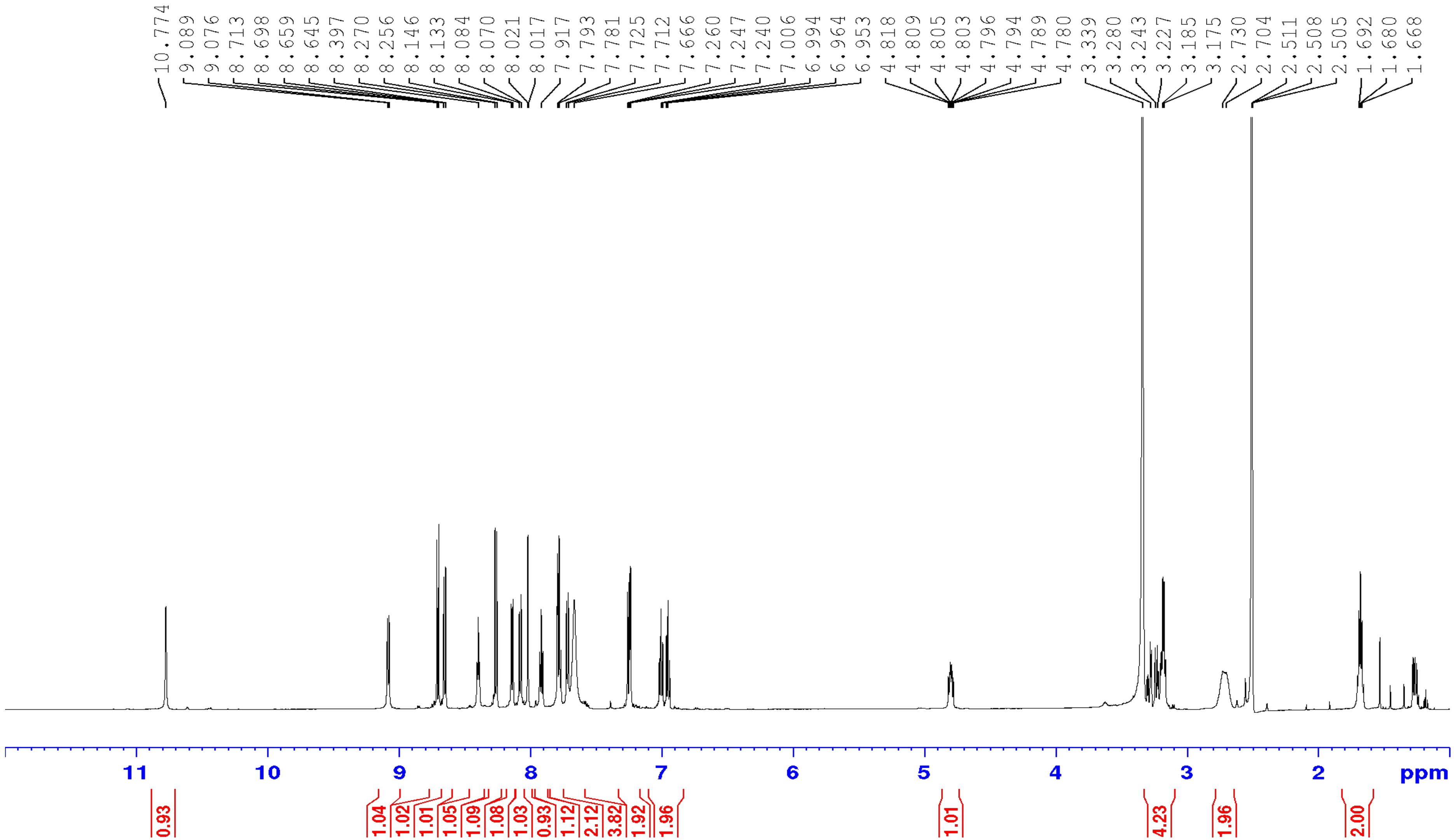

^{13}C NMR of (S)-N-(2-((1-((3-aminopropyl)amino)-3-(1*H*-indol-3-yl)-1-oxopropan-2-yl)carbamoyl)-4-bromophenyl)quinoline-2-carboxamide (13e)

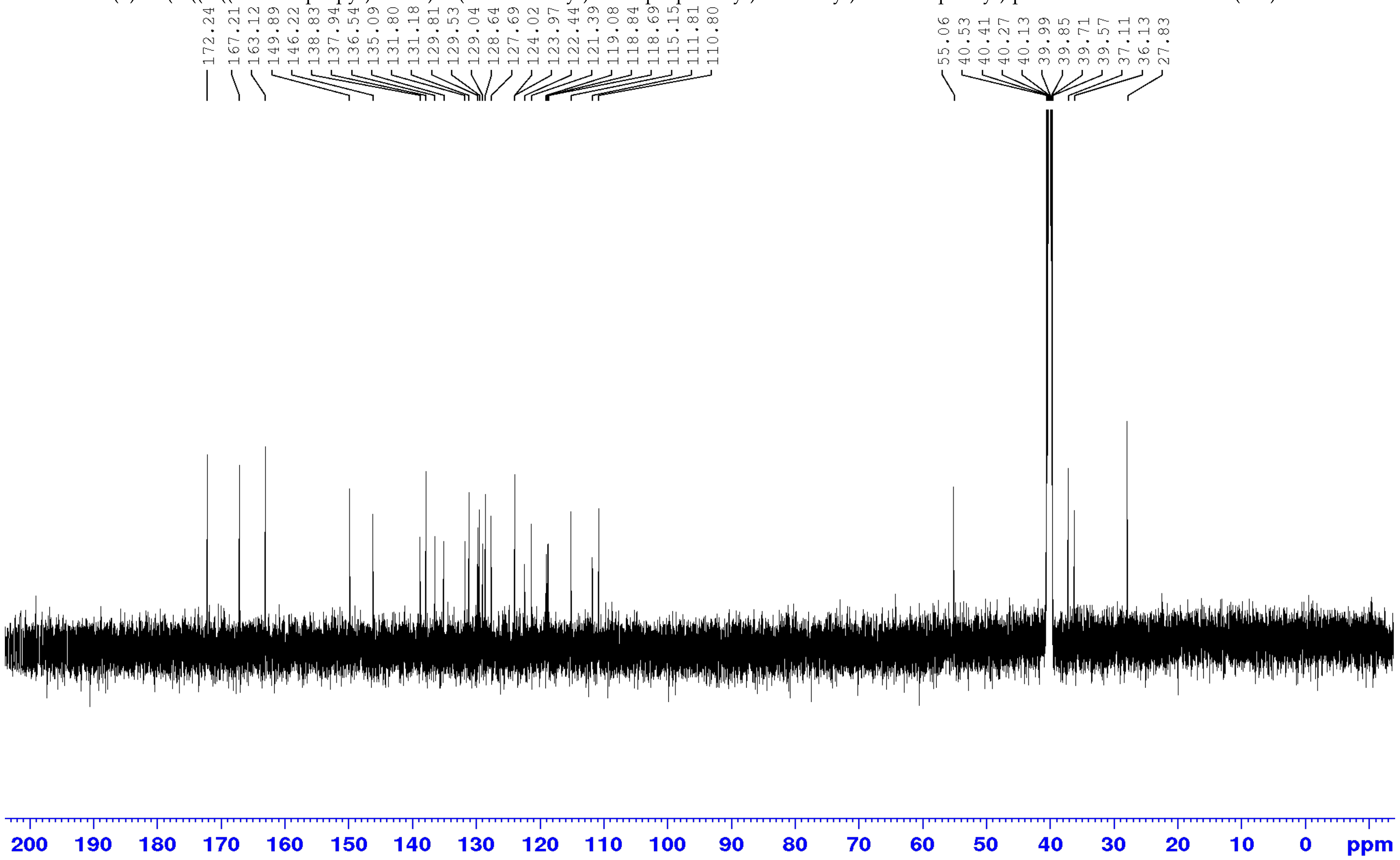

^{1}H NMR of *tert*-butyl (S)-(2-(2-(4-amino-4'-(tert-butyl)-[1,1'-biphenyl]-3-carboxamido)-3-(1*H*-indol-3-yl)propanamido)ethyl)carbamate (15a)

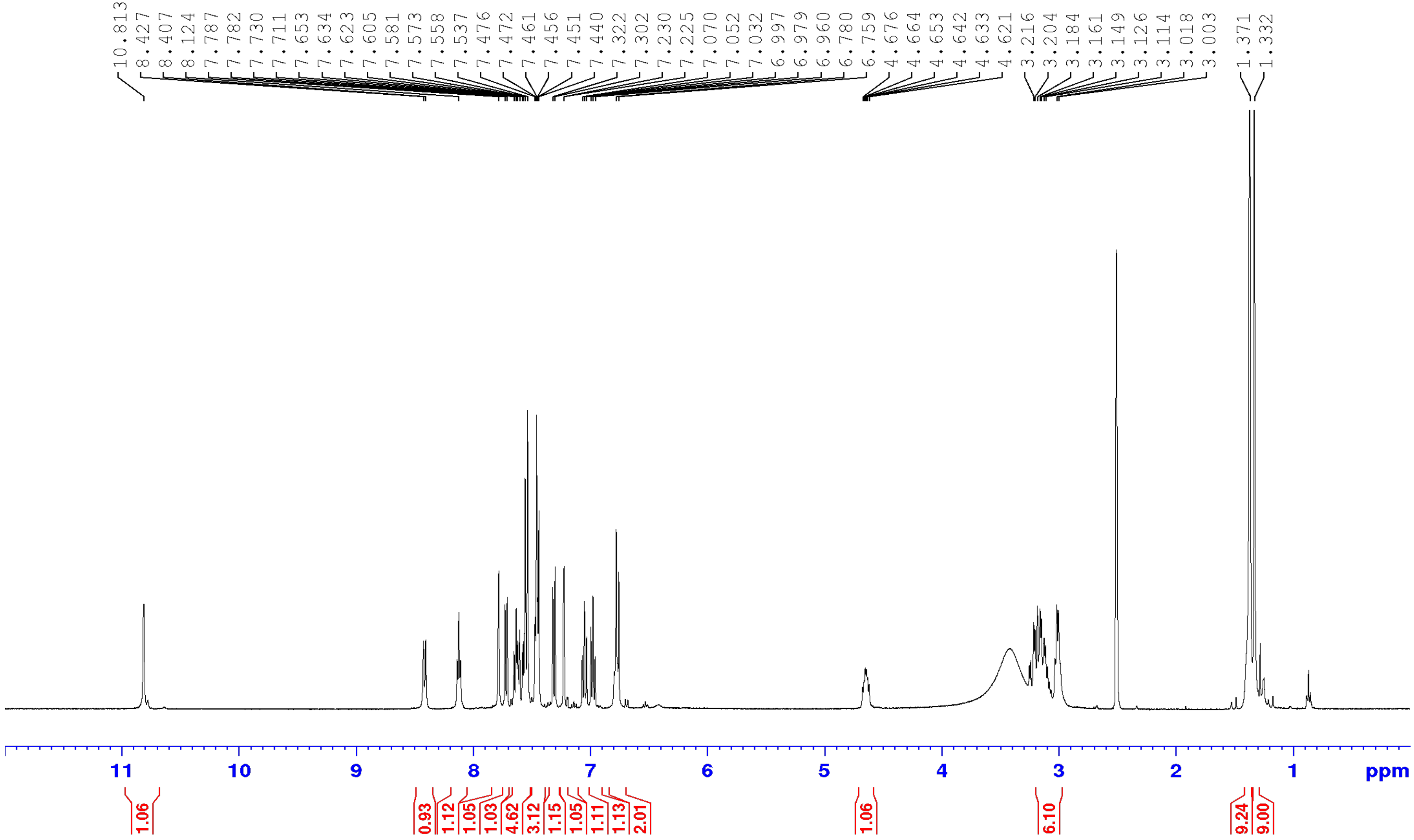

^{13}C NMR of *tert*-butyl (S)-(2-(2-(4-amino-4'-(tert-butyl)-[1,1'-biphenyl]-3-carboxamido)-3-(1*H*-indol-3-yl)propanamido)ethyl)carbamate (15a)

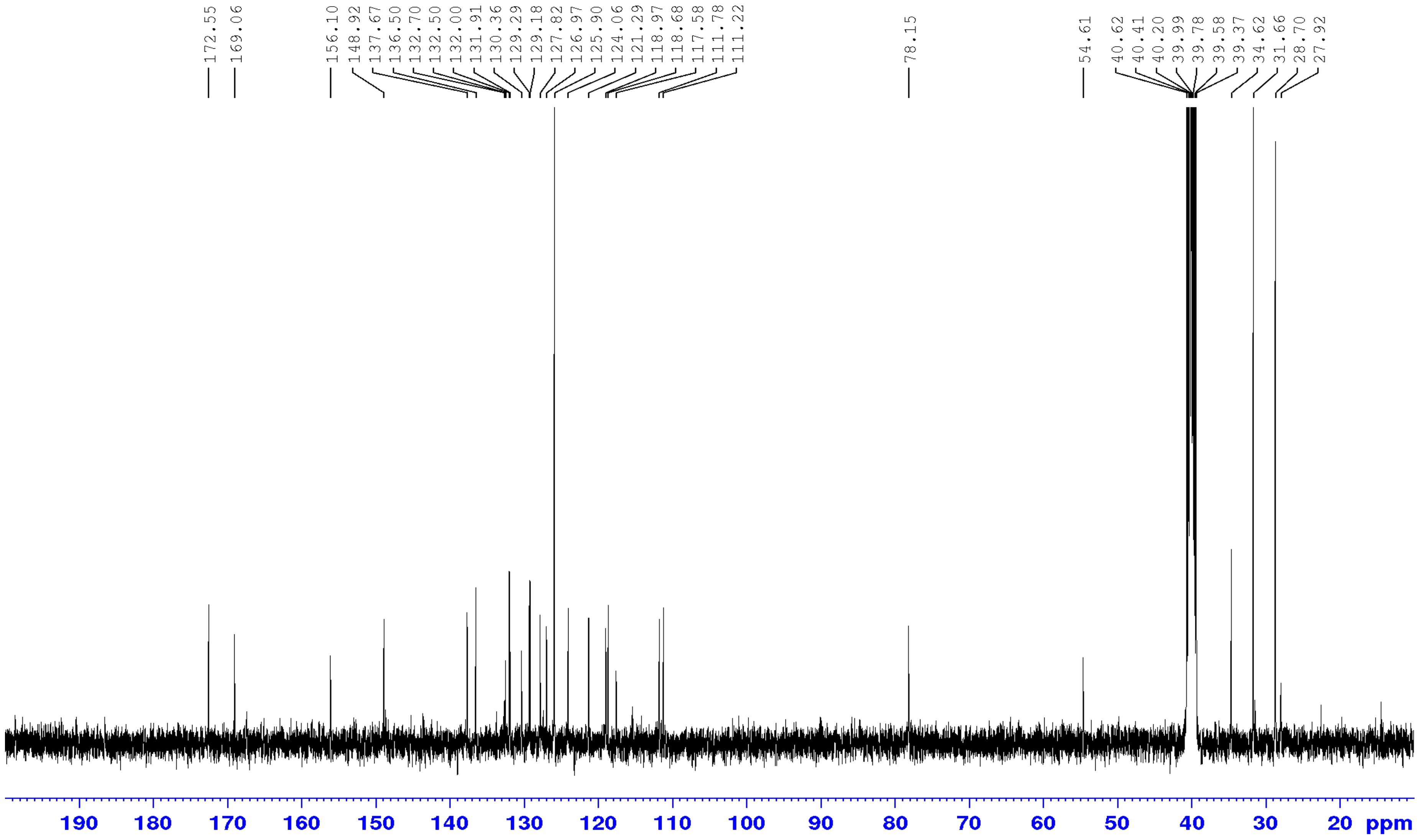

^{1}H NMR of *tert*-butyl (S)-(2-(2-(2-amino-5-(naphthalen-2-yl)benzamido)-3-(1H-indol-3-yl)propanamido)ethyl)carbamate (15b)

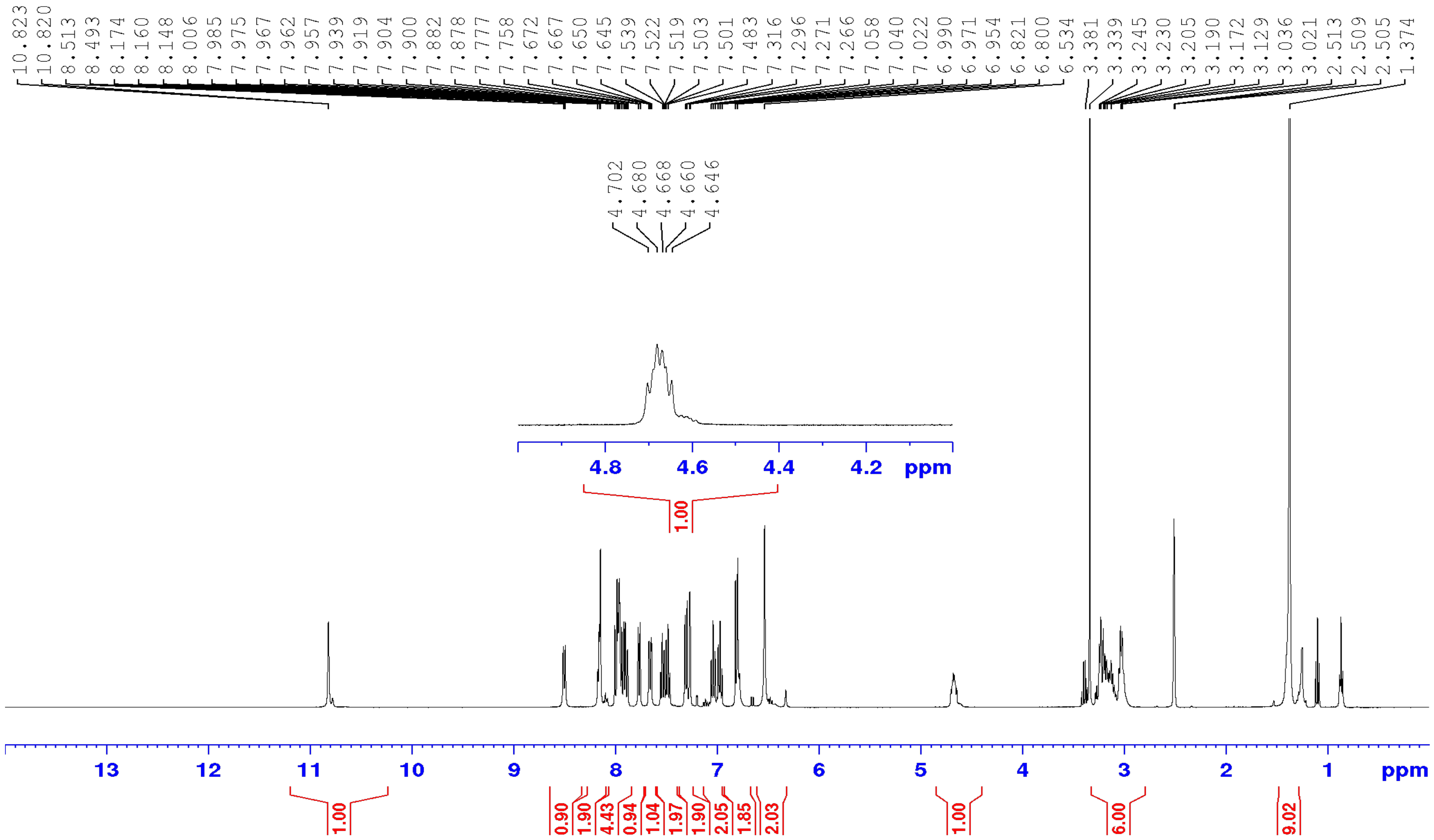

^{13}C NMR of *tert*-butyl (S)-(2-(2-(2-amino-5-(naphthalen-2-yl)benzamido)-3-(1H-indol-3-yl)propanamido)ethyl)carbamate (15b)

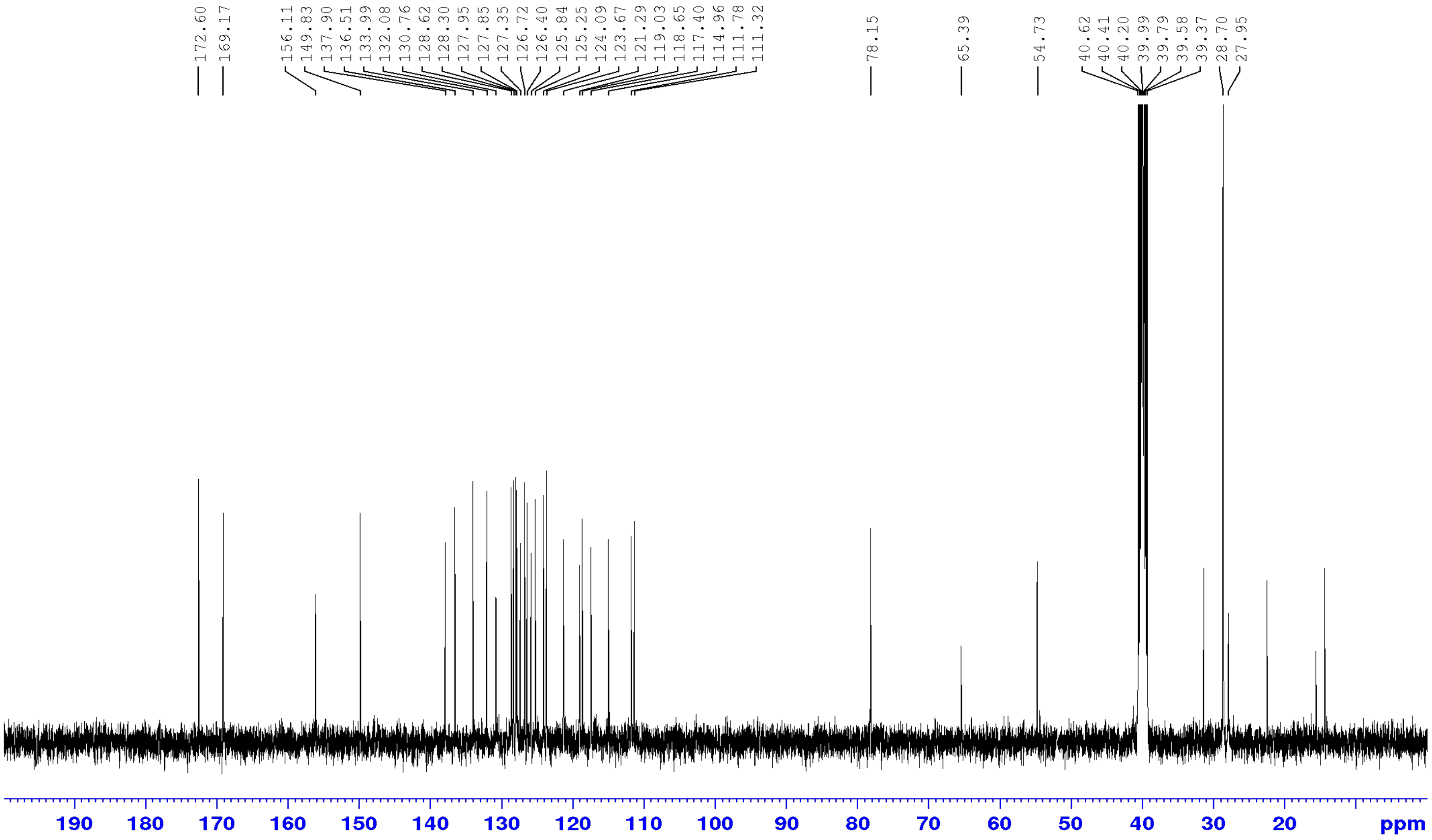

^{1}H NMR of *tert*-butyl (S)-(2-(2-(4-amino-4'-fluoro-[1,1'-biphenyl]-3-carboxamido)-3-(1H-indol-3-yl)propanamido)ethyl)carbamate (15c)

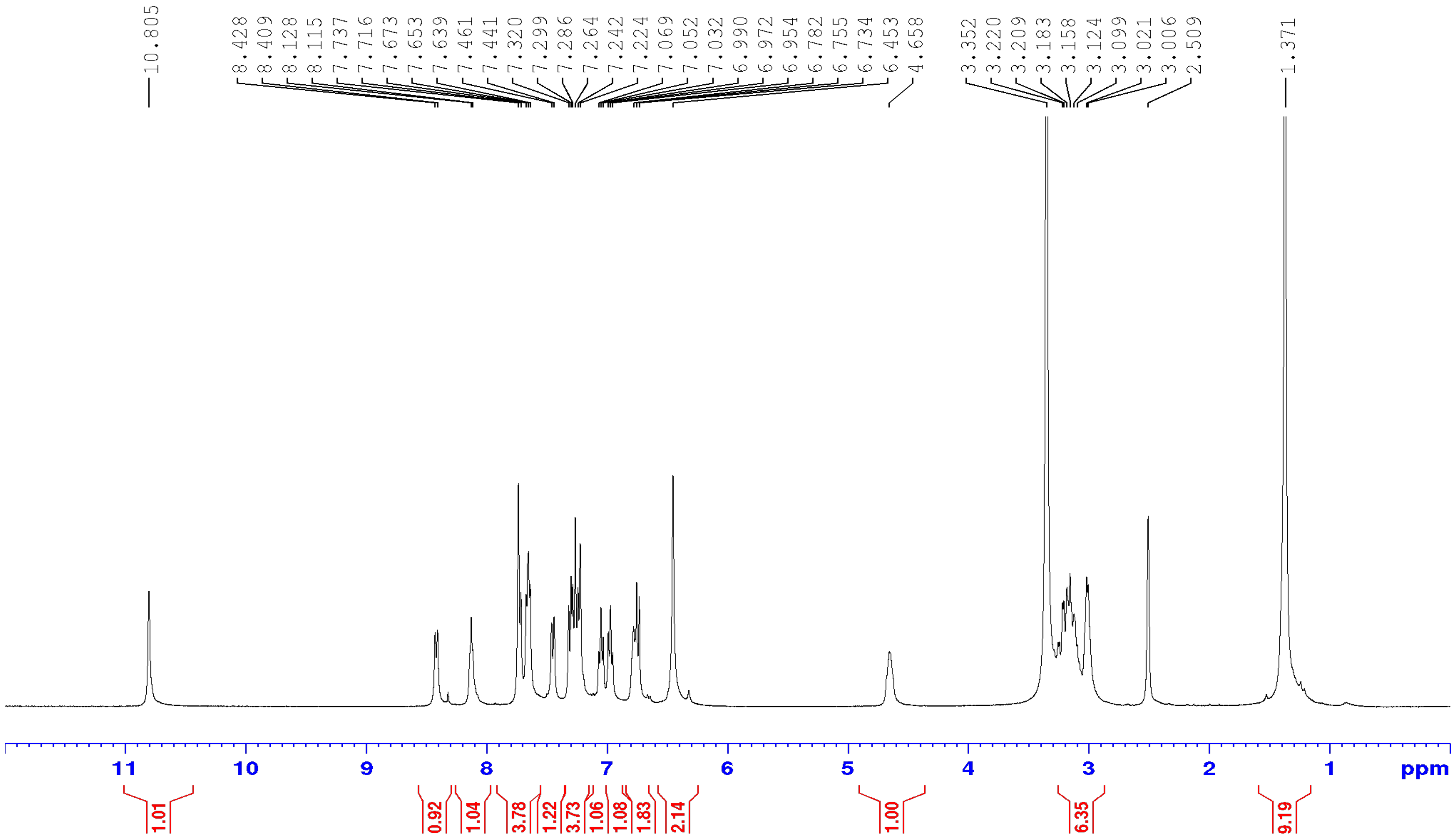

^{13}C NMR of *tert*-butyl (S)-(2-(2-(4-amino-4'-fluoro-[1,1'-biphenyl]-3-carboxamido)-3-(1H-indol-3-yl)propanamido)ethyl)carbamate (15c)

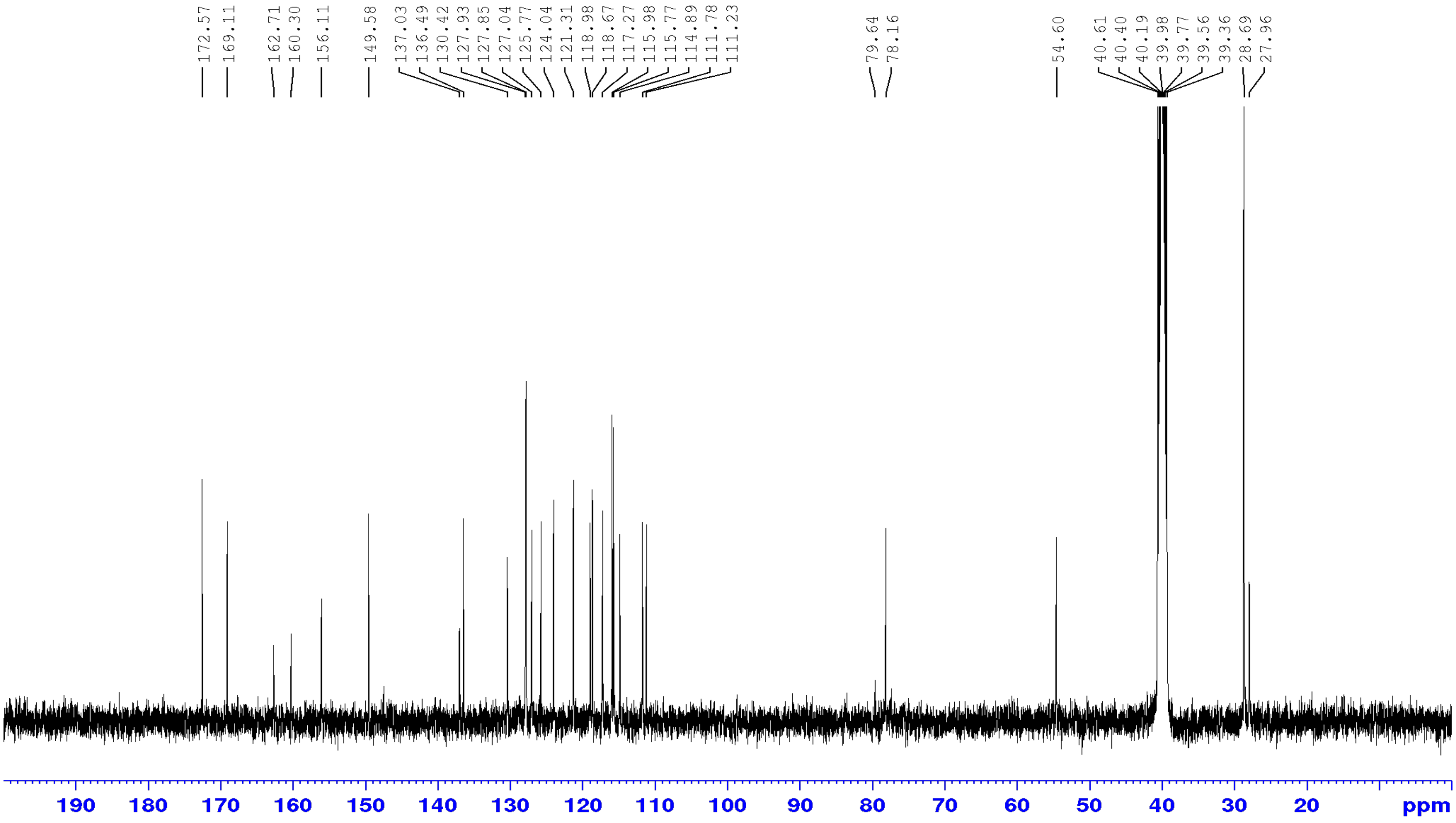

^{1}H NMR of *tert*-butyl (S)-(2-(2-(4-amino-4'-(trifluoromethyl)-[1,1'-biphenyl]-3-carboxamido)-3-(1H-indol-3-yl)propanamido)ethyl)carbamate (15d)

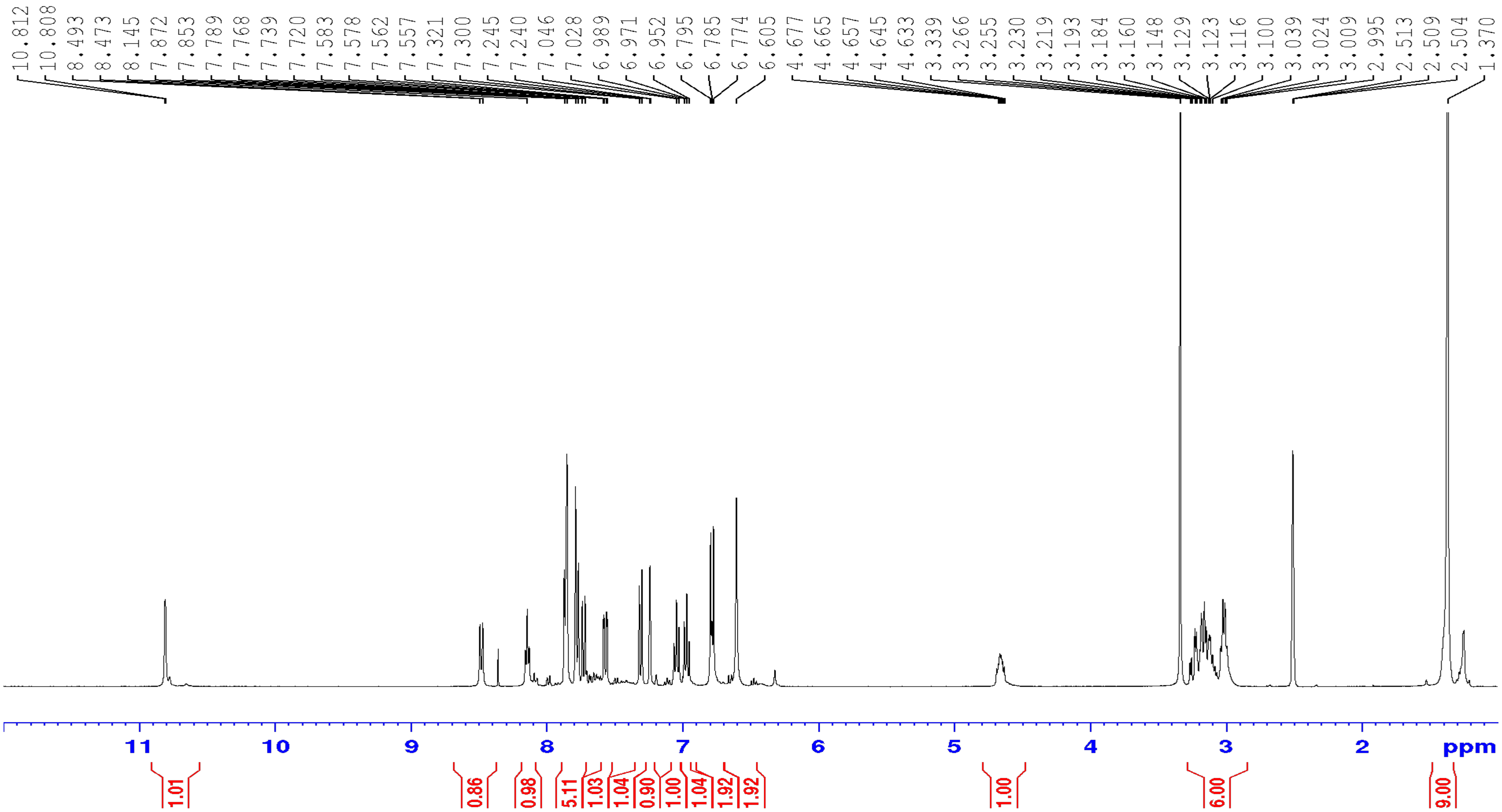

^{13}C NMR of *tert*-butyl (S)-(2-(2-(4-amino-4'-(trifluoromethyl)-[1,1'-biphenyl]-3-carboxamido)-3-(1H-indol-3-yl)propanamido)ethyl)carbamate (15d)

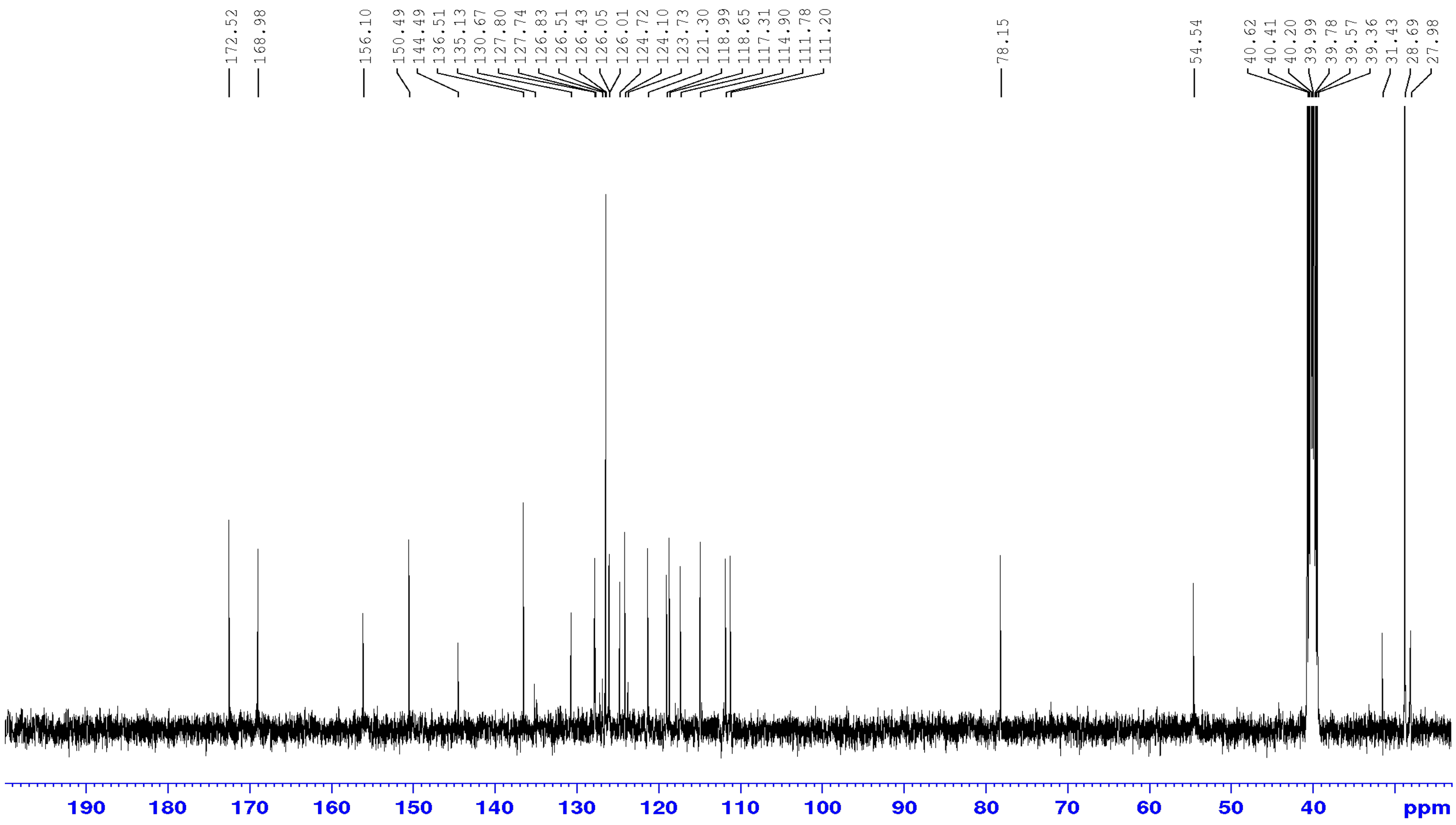

1H NMR of (S)-4-amino-N-(1-((2-aminoethyl)amino)-3-(1H-indol-3-yl)-1-oxopropan-2-yl)-4'-(tert-butyl)-[1,1'-biphenyl]-3-carboxamide (16a)

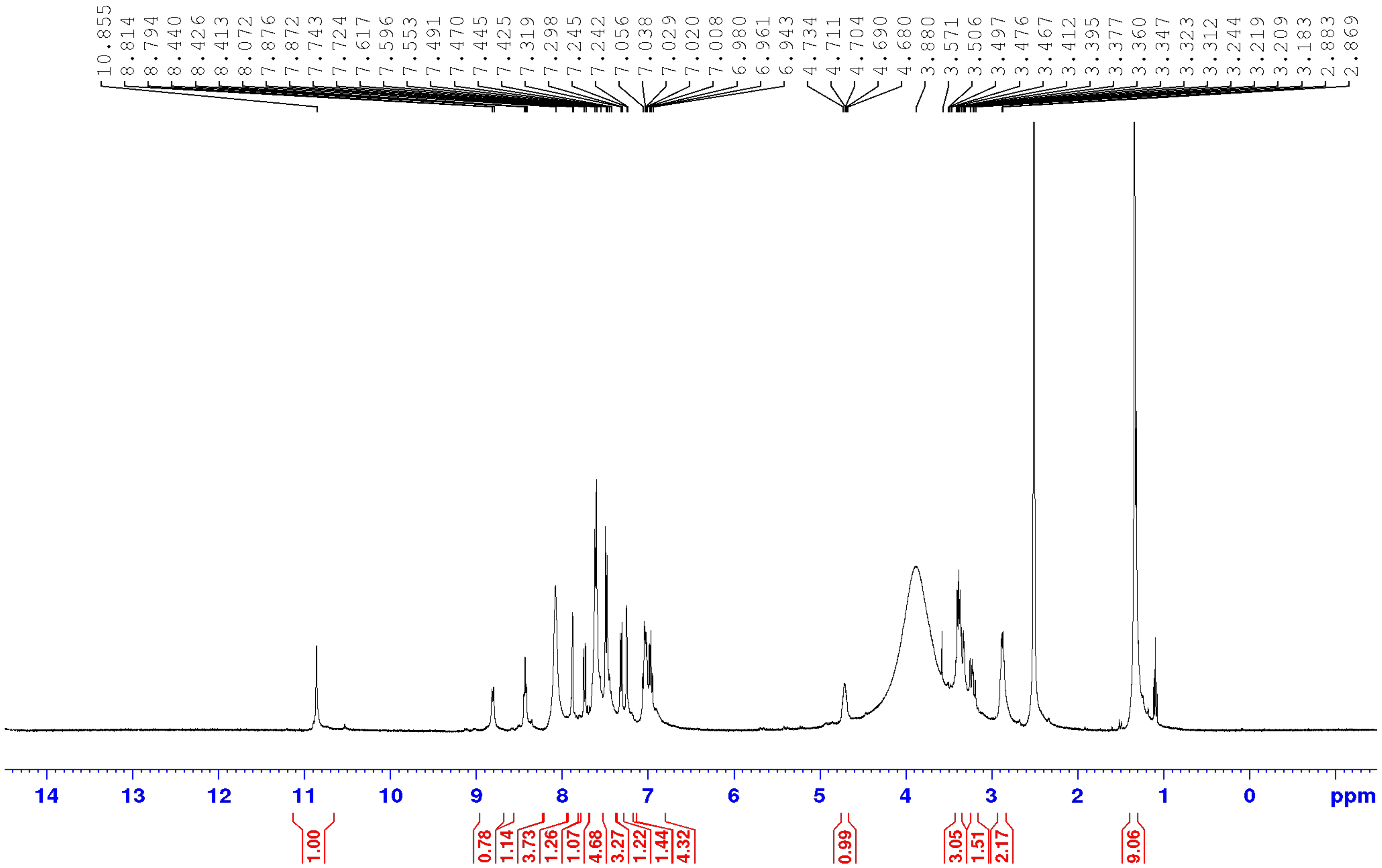

13C NMR of (S)-4-amino-N-(1-((2-aminoethyl)amino)-3-(1H-indol-3-yl)-1-oxopropan-2-yl)-4'-(tert-butyl)-[1,1'-biphenyl]-3-carboxamide (16a)

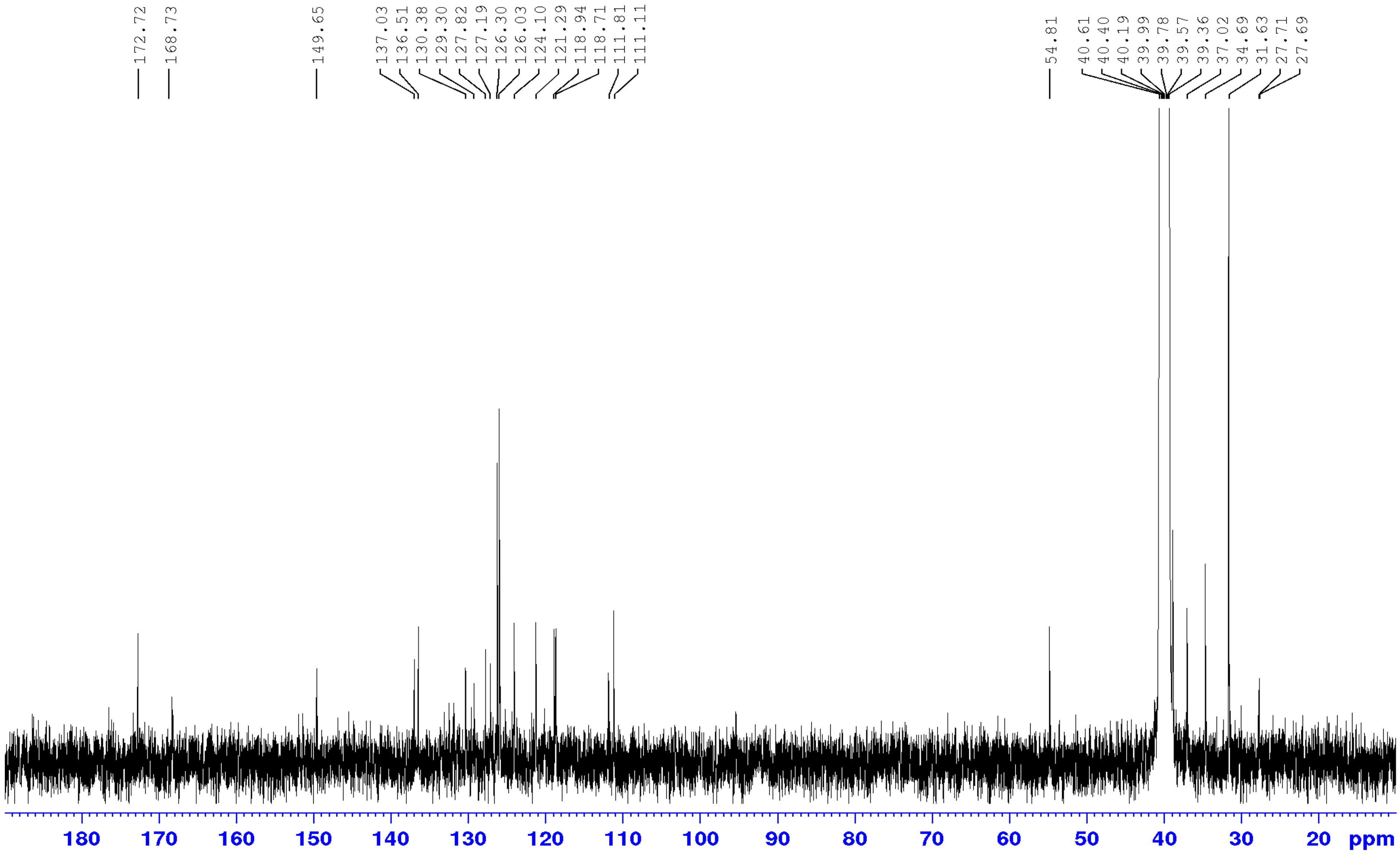

^{1}H NMR of (S)-2-amino-N-(1-((2-aminoethyl)amino)-3-(1*H*-indol-3-yl)-1-oxopropan-2-yl)-5-(naphthalen-2-yl)benzamide (16b)

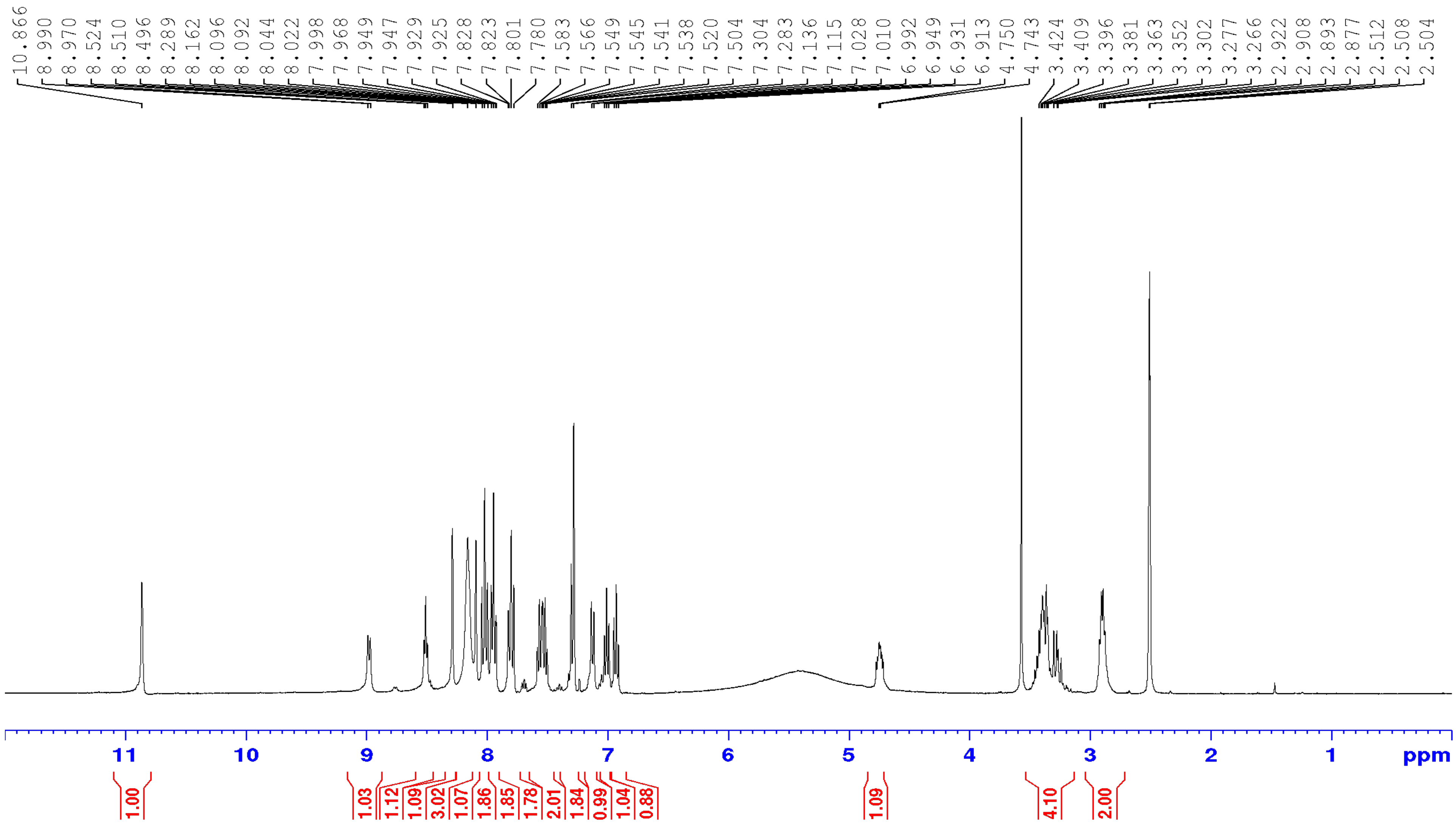

^{13}C NMR of (S)-2-amino-N-(1-((2-aminoethyl)amino)-3-(1*H*-indol-3-yl)-1-oxopropan-2-yl)-5-(naphthalen-2-yl)benzamide (16b)

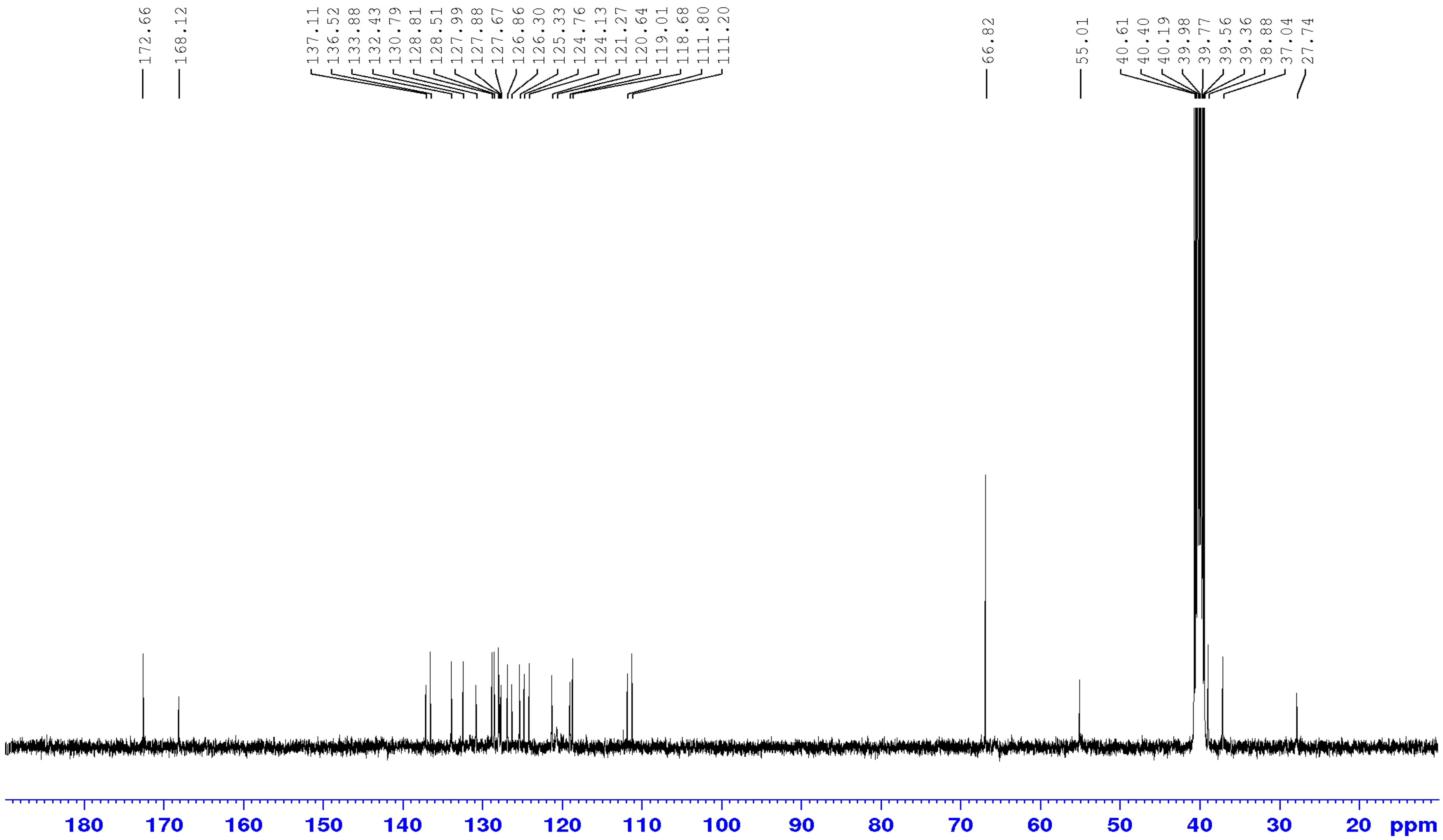

¹H NMR of (S)-4-amino-N-(1-((2-aminoethyl)amino)-3-(1H-indol-3-yl)-1-oxopropan-2-yl)-4'-fluoro-[1,1'-biphenyl]-3-carboxamide (16c)

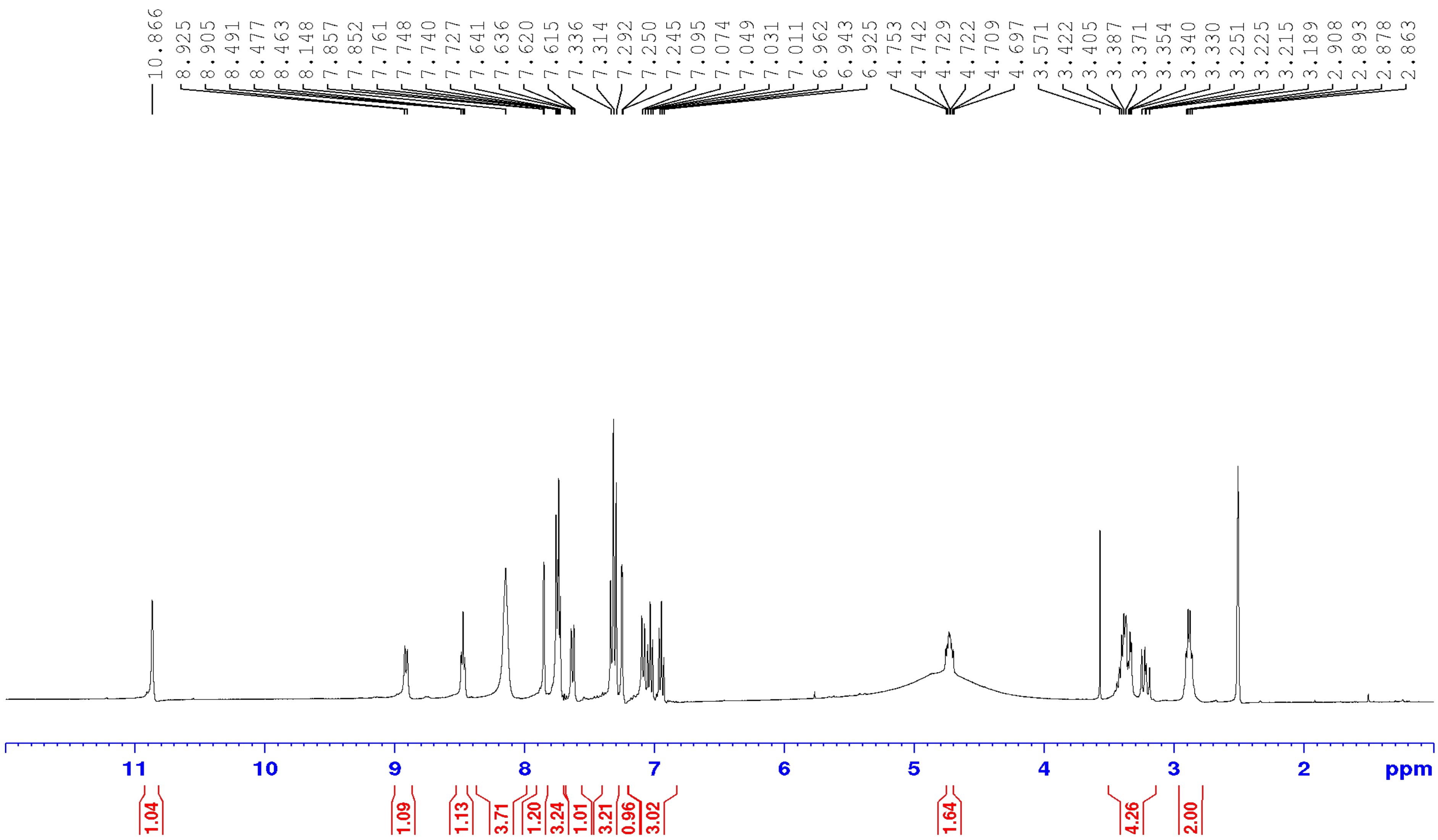

^{1}H NMR of (S)-4-amino-N-(1-((2-aminoethyl)amino)-3-(1H-indol-3-yl)-1-oxopropan-2-yl)-4'-(trifluoromethyl)-[1,1'-biphenyl]-3-carboxamide (16d)

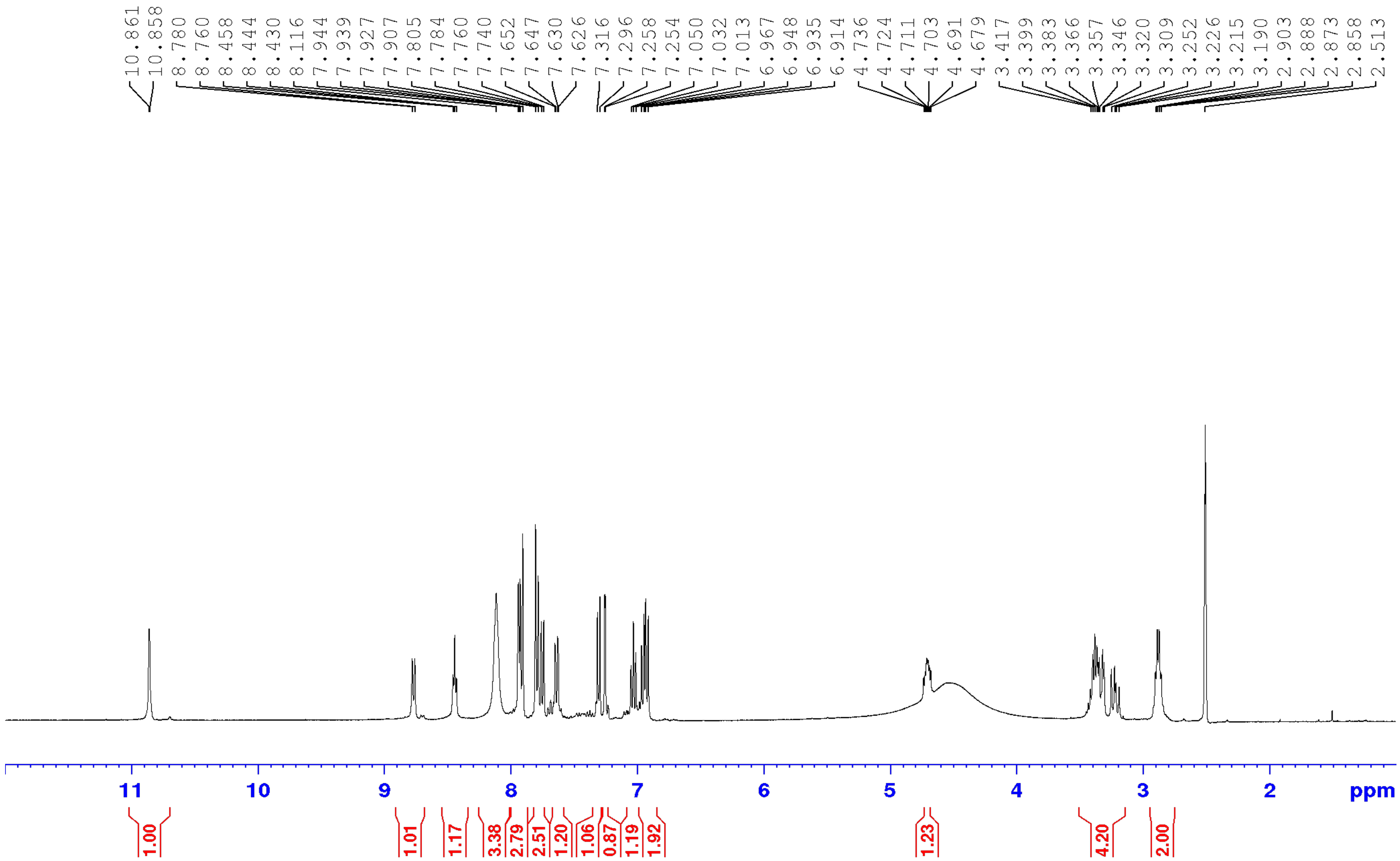

13C NMR of (S)-4-amino-N-(1-((2-aminoethyl)amino)-3-(1H-indol-3-yl)-1-oxopropan-2-yl)-4'-(trifluoromethyl)-[1,1'-biphenyl]-3-carboxamide (16d)

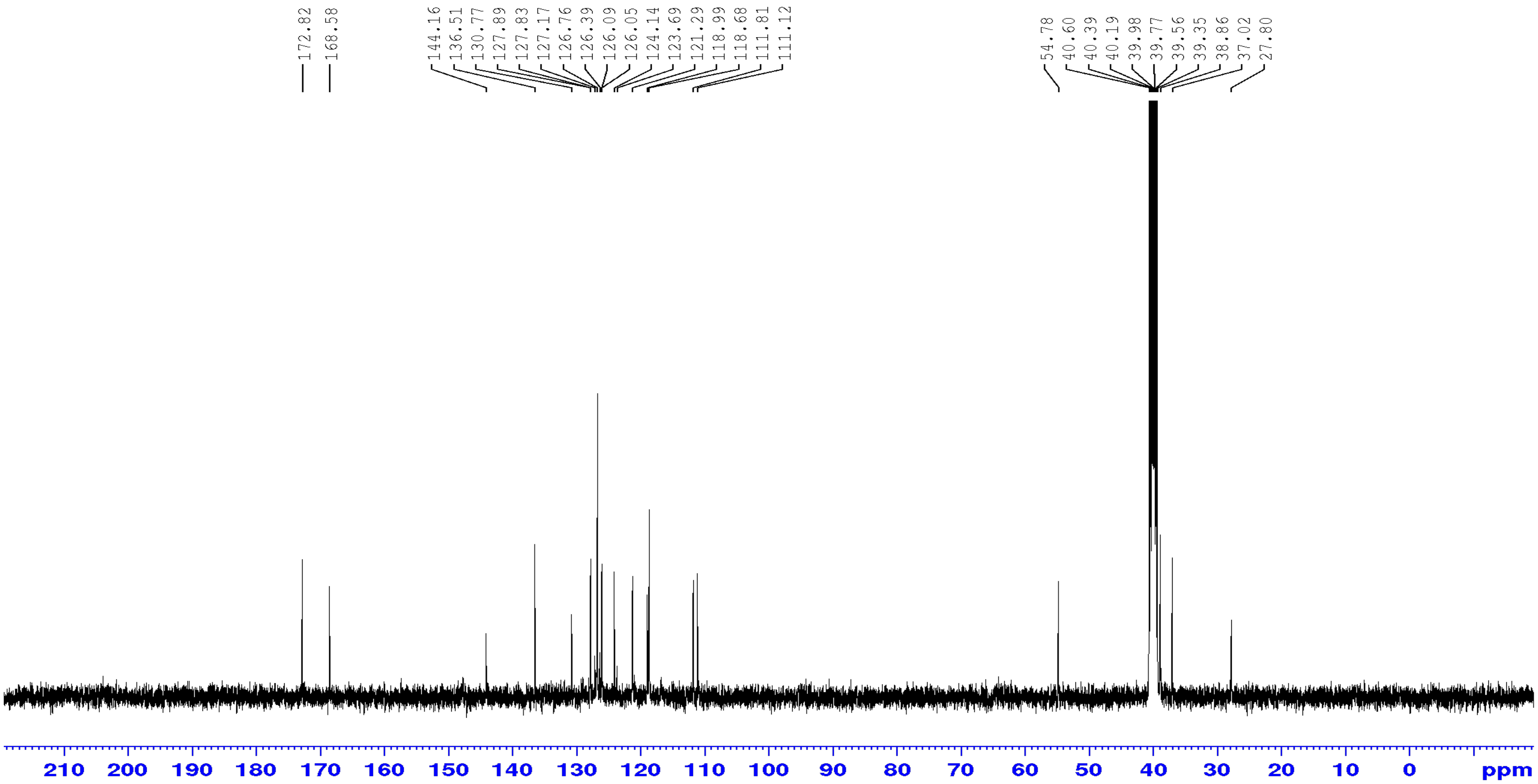

^{1}H NMR of di-*tert*-butyl ((S)-6-((2-((S)-2-(2-(2-naphthamido)-5-bromobenzamido)-3-(1H-indol-3-yl)propanamido)ethyl)amino)-6-oxohexane-1,5-diyl)dicarbamate (18a)

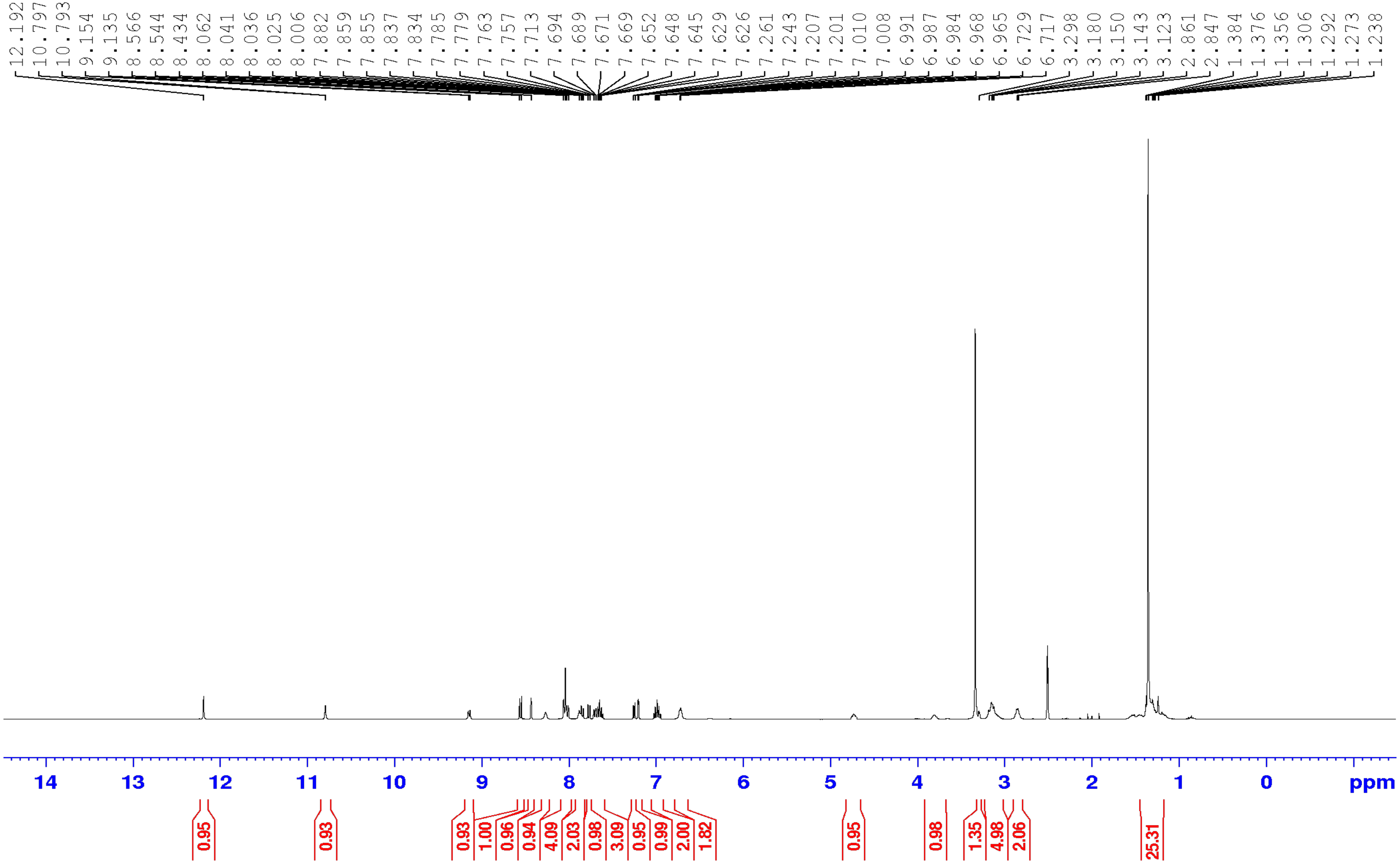

^{13}C NMR of di-*tert*-butyl ((S)-6-((2-((S)-2-(2-(2-naphthamido)-5-bromobenzamido)-3-(1H-indol-3-yl)propanamido)ethyl)amino)-6-oxohexane-1,5-diyl)dicarbamate (18a)

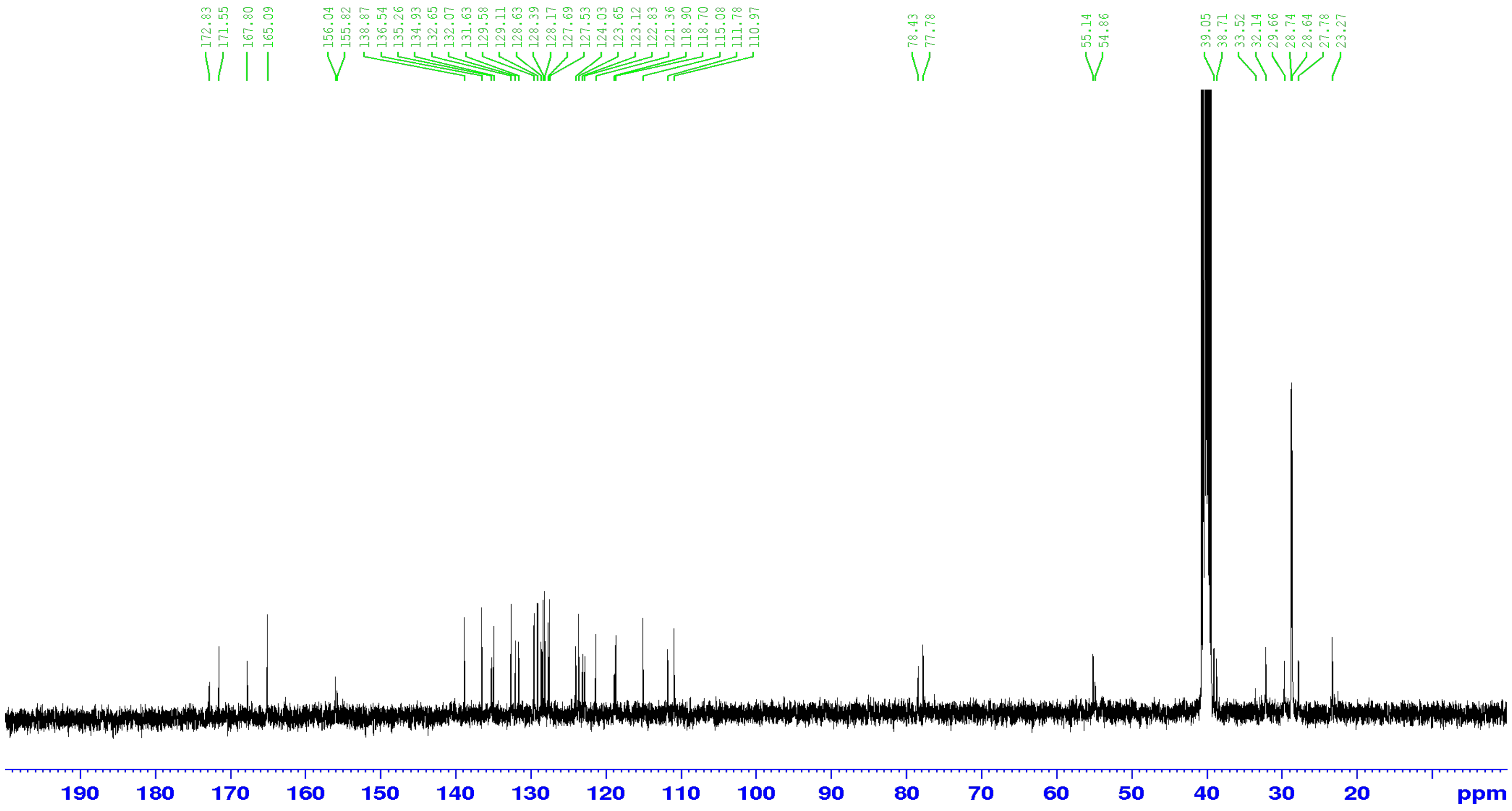

^{1}H NMR of *di-tert*-butyl ((S)-6-((2-((S)-2-(2-([1,1'-biphenyl]-3-carboxamido)-5-bromobenzamido)-3-(1H-indol-3-yl)propanamido)ethyl)amino)-6-oxohexane-1,5-diyl)dicarbamate (**18b**)

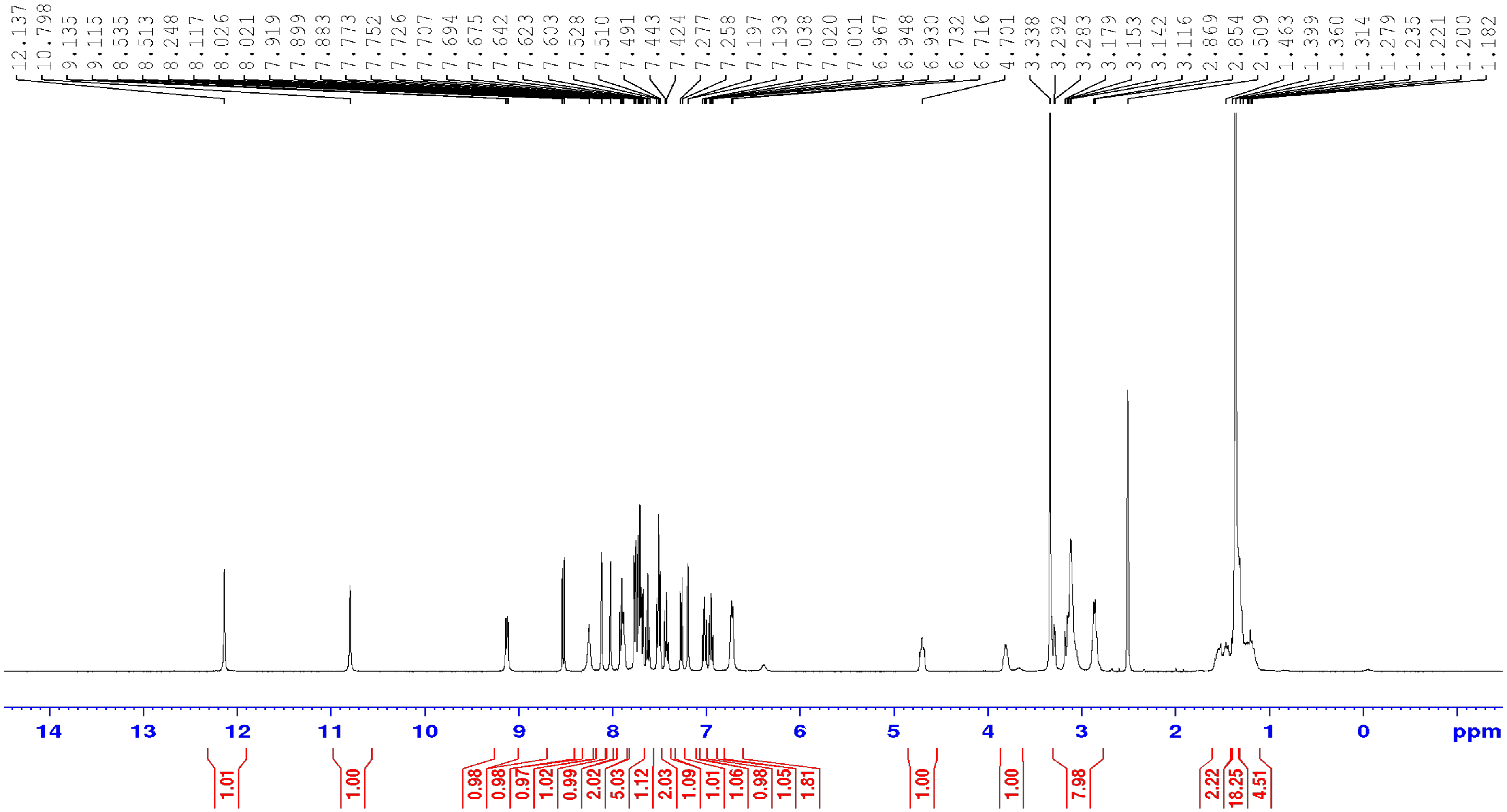

^{13}C NMR of di-*tert*-butyl ((S)-6-((2-((S)-2-(2-([1,1'-biphenyl]-3-carboxamido)-5-bromobenzamido)-3-(1H-indol-3-yl)propanamido)ethyl)amino)-6-oxohexane-1,5-diyl)dicarbamate (18b)

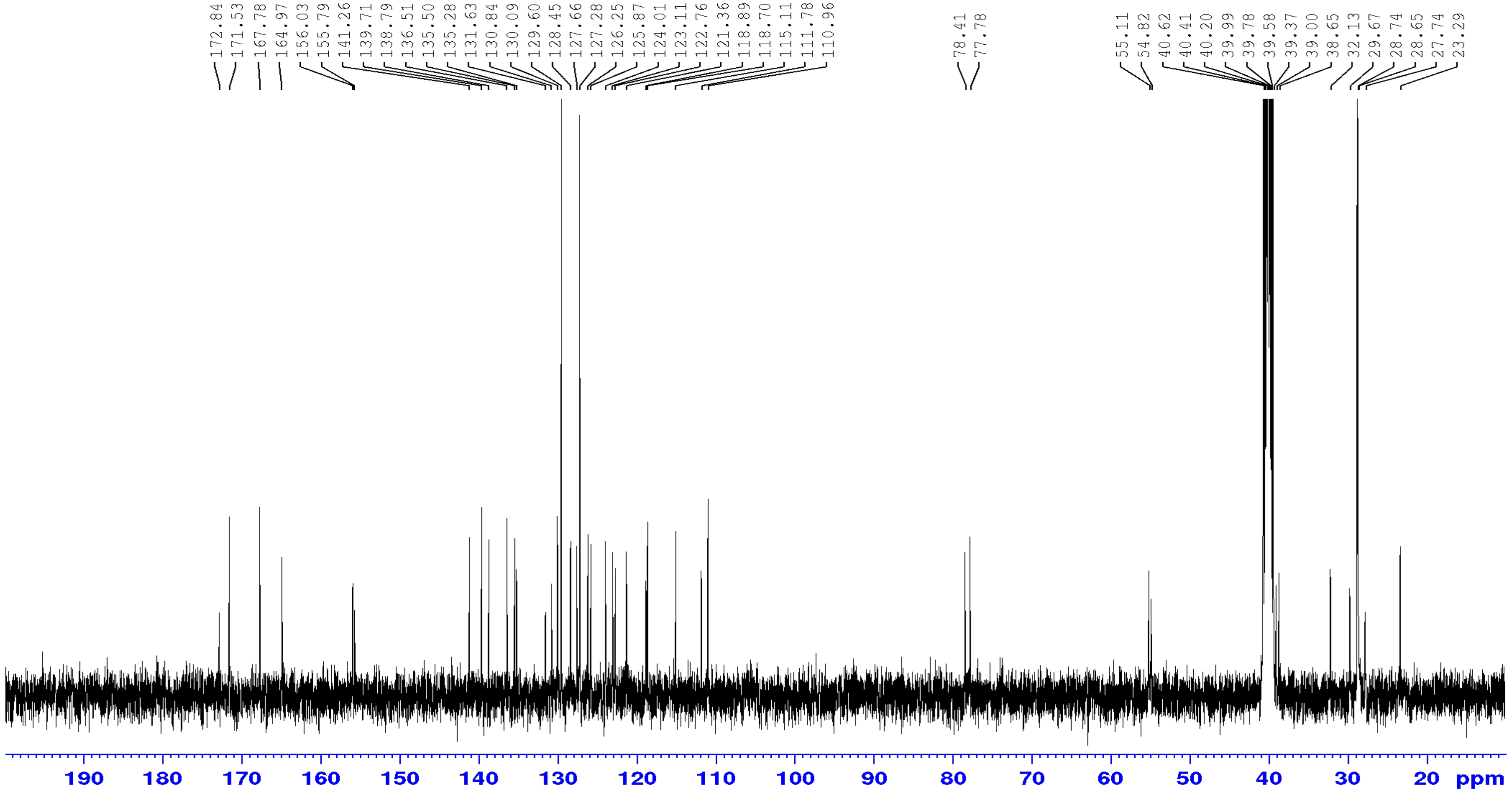

^{1}H NMR of di-*tert*-butyl ((S)-6-((2-((S)-2-(2-([1,1'-biphenyl]-4-carboxamido)-5-bromobenzamido)-3-(1H-indol-3-yl)propanamido)ethyl)amino)-6-oxohexane-1,5-diyl)dicarbamate (18c)

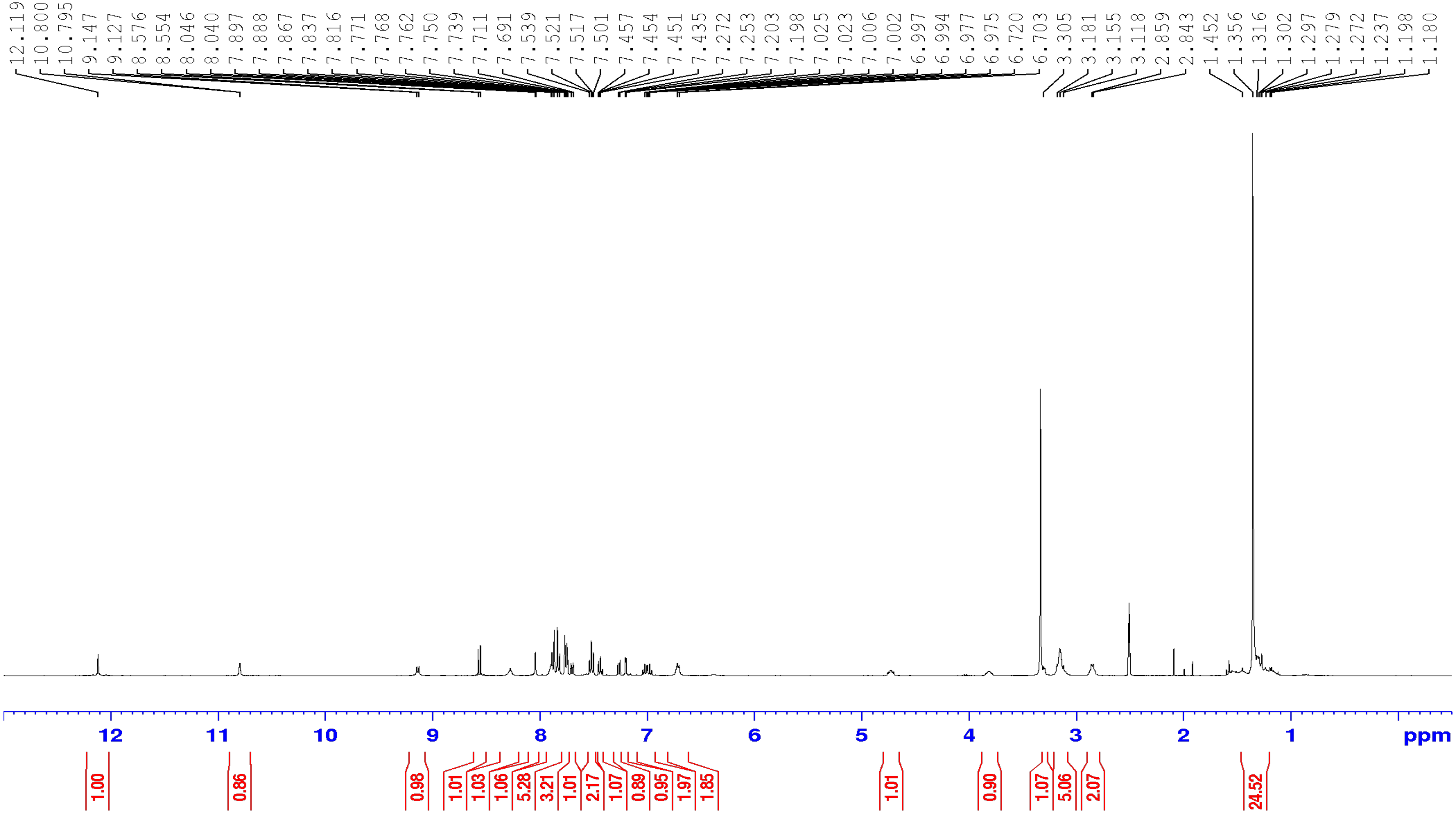

^{13}C NMR of di-*tert*-butyl ((S)-6-((2-((S)-2-(2-([1,1'-biphenyl]-4-carboxamido)-5-bromobenzamido)-3-(1H-indol-3-yl)propanamido)ethyl)amino)-6-oxohexane-1,5-diyl)dicarbamate (18c)

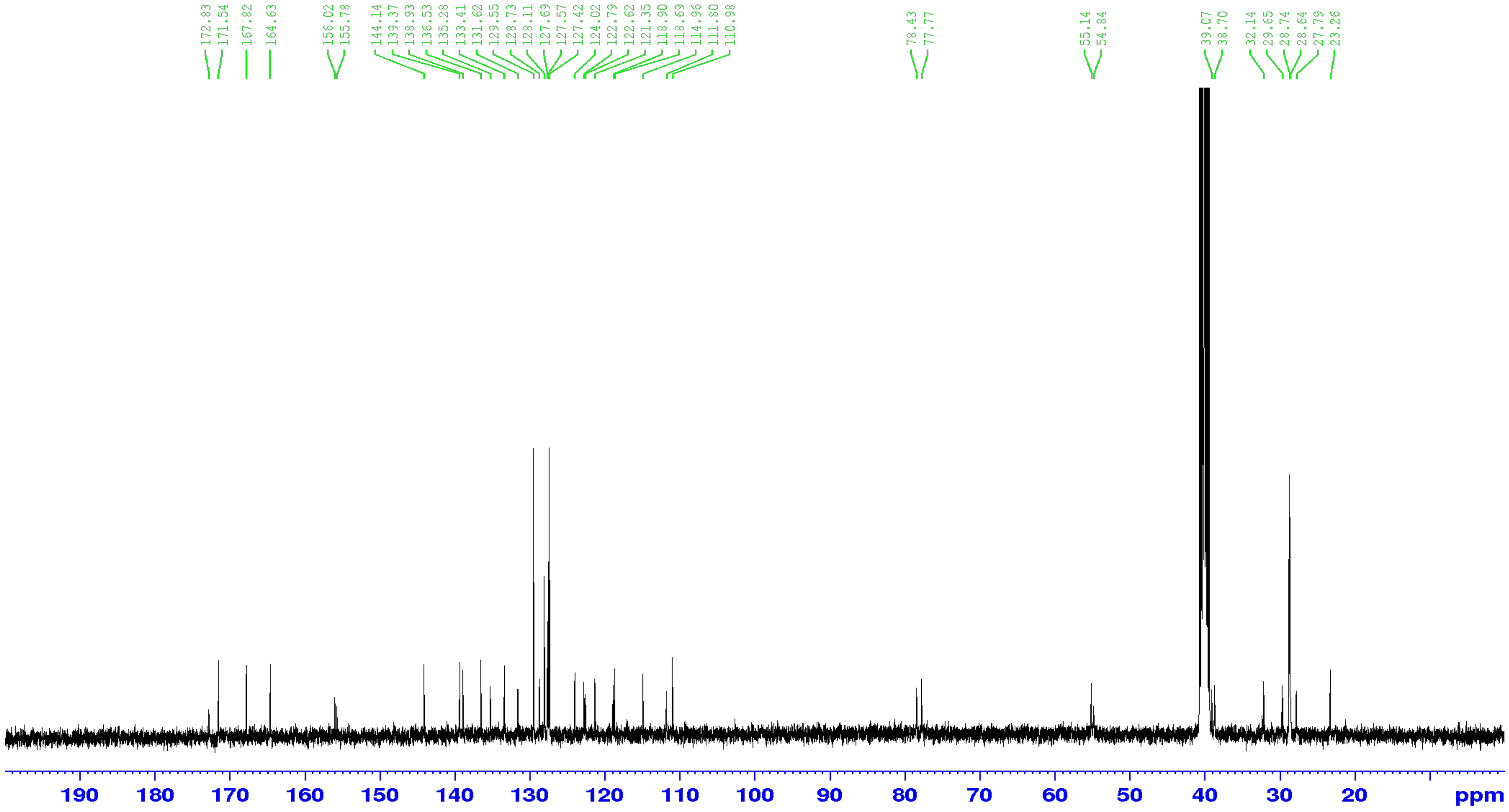

^{1}H NMR of *N*-(4-bromo-2-(((S)-1-((2-((S)-2,6-diaminohexanamido)ethyl)amino)-3-(1*H*-indol-3-yl)-1-oxopropan-2-yl)carbamoyl)phenyl)-2-naphthamide (19a)

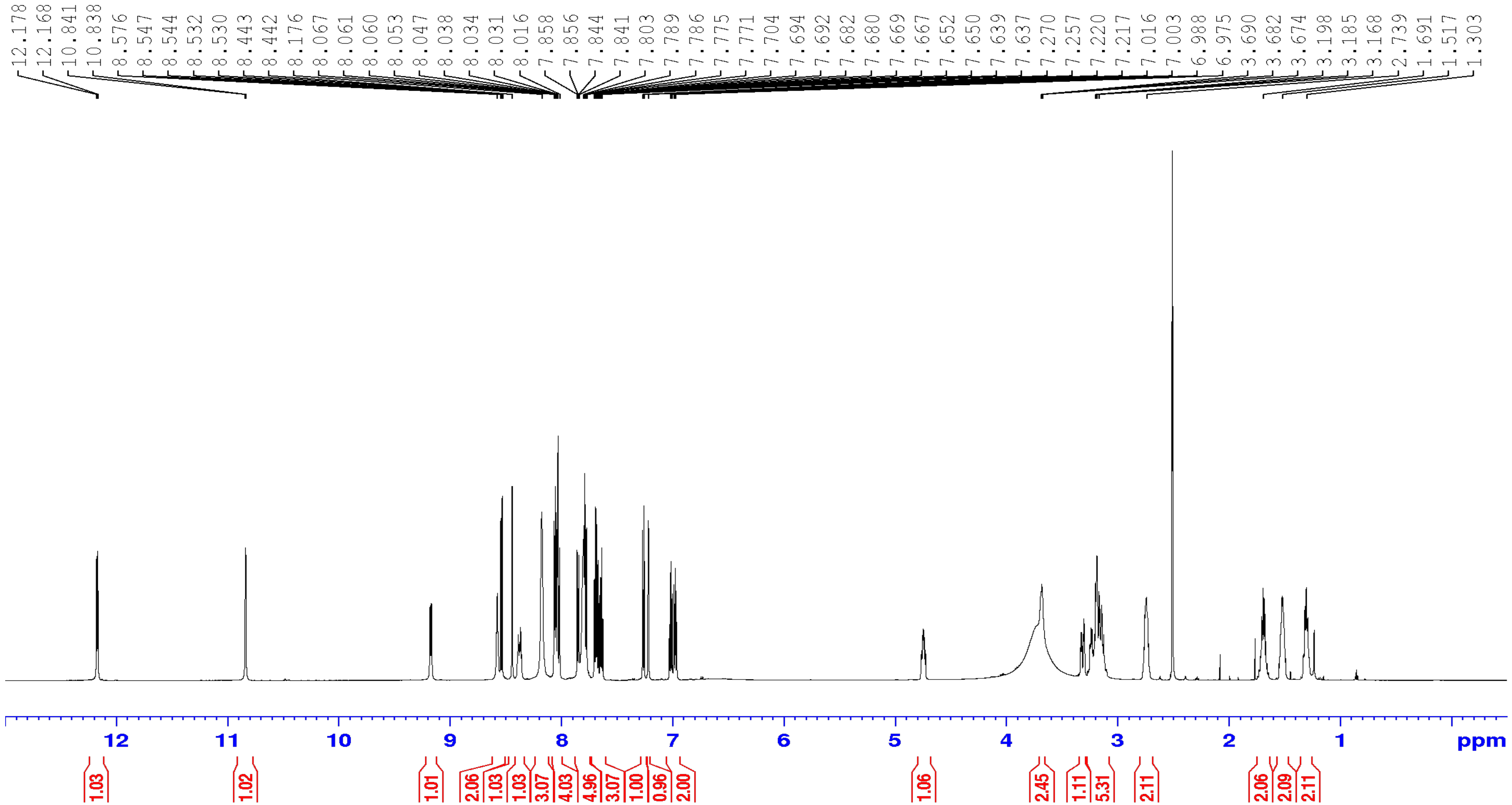

^{13}C NMR of *N*-(4-bromo-2-(((S)-1-((2-((S)-2,6-diaminohexanamido)ethyl)amino)-3-(1*H*-indol-3-yl)-1-oxopropan-2-yl)carbamoyl)phenyl)-2-naphthamide (19a)

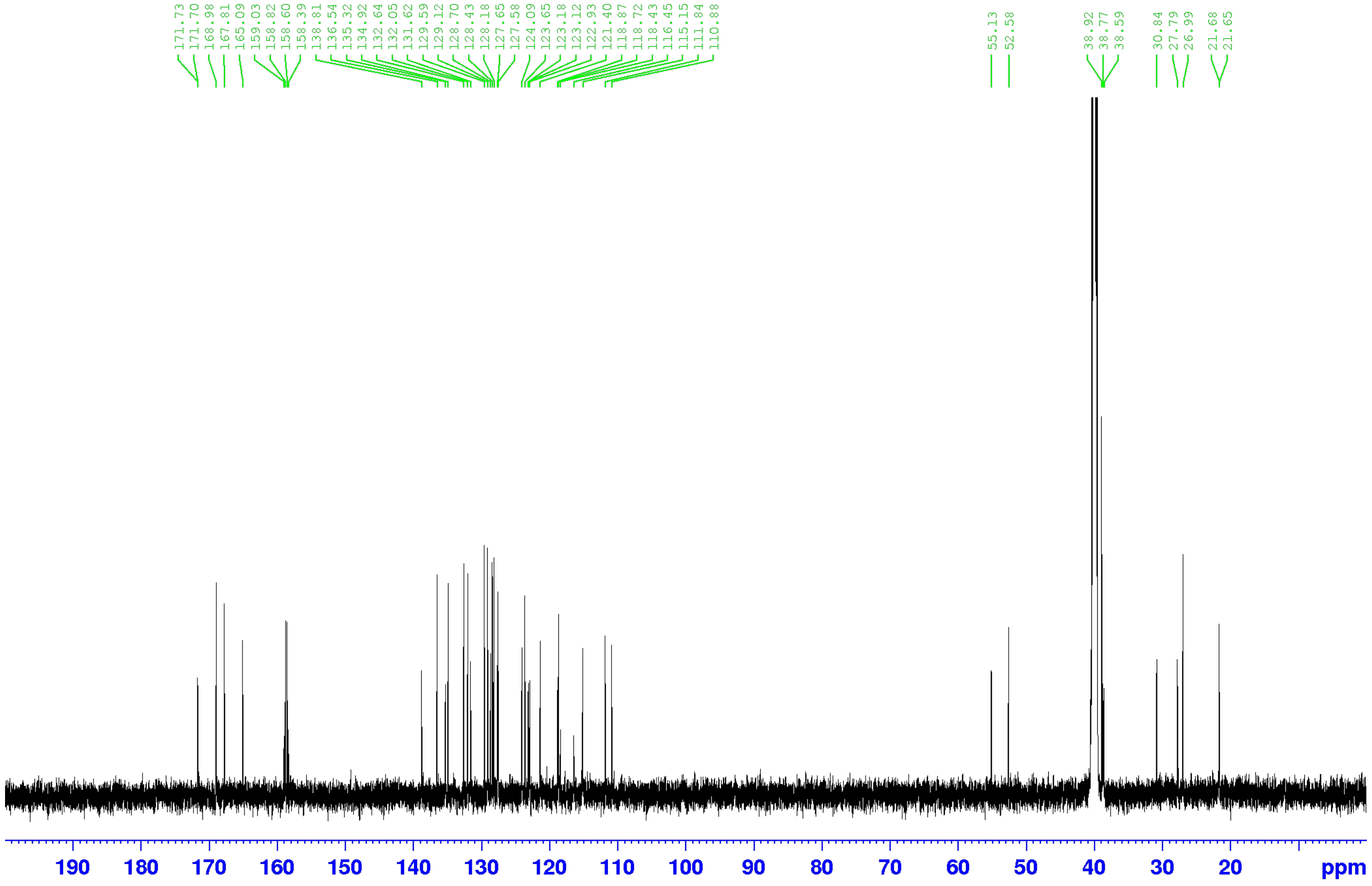

^{1}HNMR of *N*-(4-bromo-2-(((S)-1-((2-((S)-2,6-diaminohexanamido)ethyl)amino)-3-(1*H*-indol-3-yl)-1-oxopropan-2-yl)carbamoyl)phenyl)-[1,1'-biphenyl]-3-carboxamide (19b)

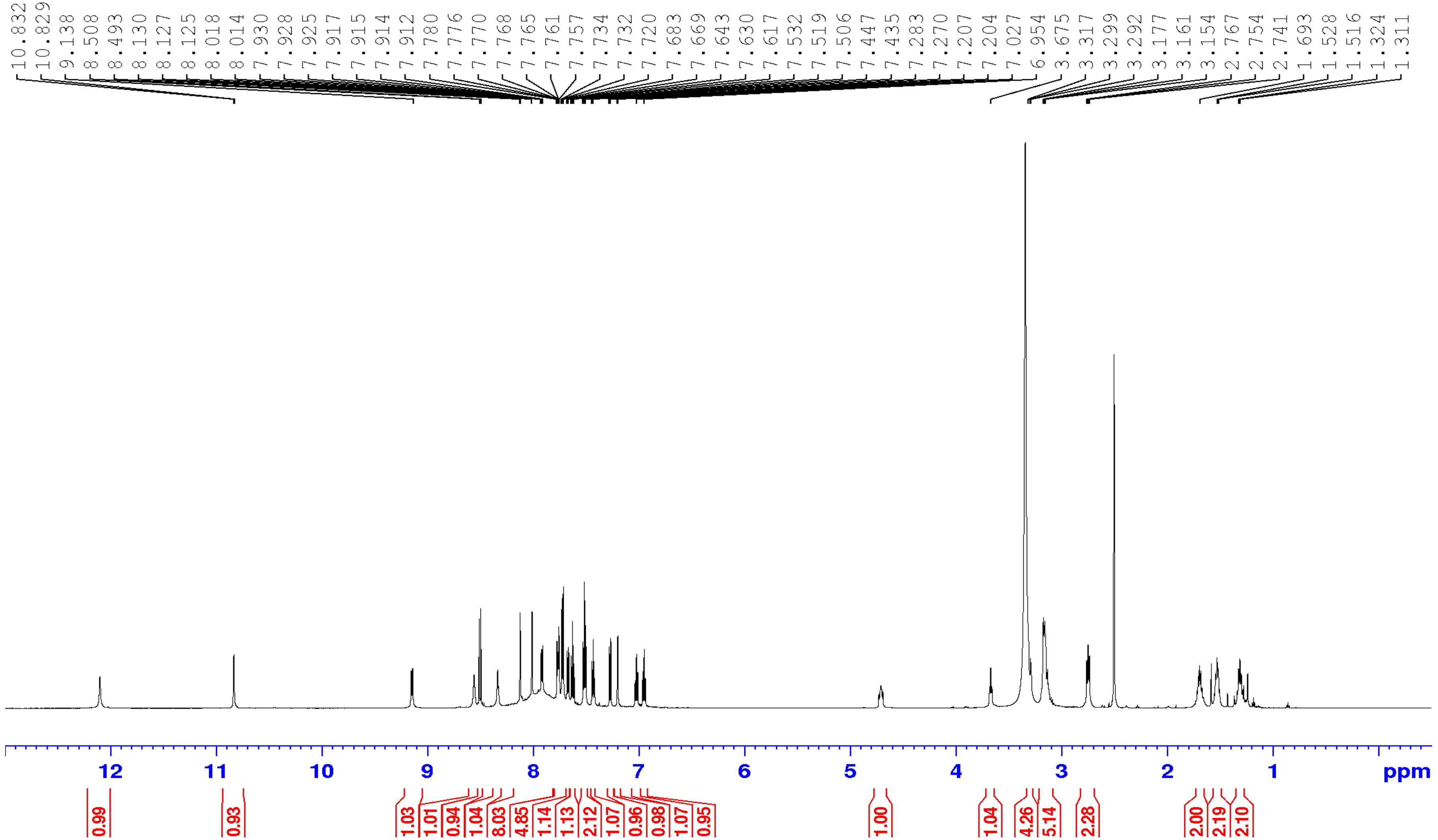

^{13}C NMR of *N*-(4-bromo-2-(((S)-1-((2-((S)-2,6-diaminohexanamido)ethyl)amino)-3-(1*H*-indol-3-yl)-1-oxopropan-2-yl)carbamoyl)phenyl)-[1,1'-biphenyl]-3-carboxamide (19b)

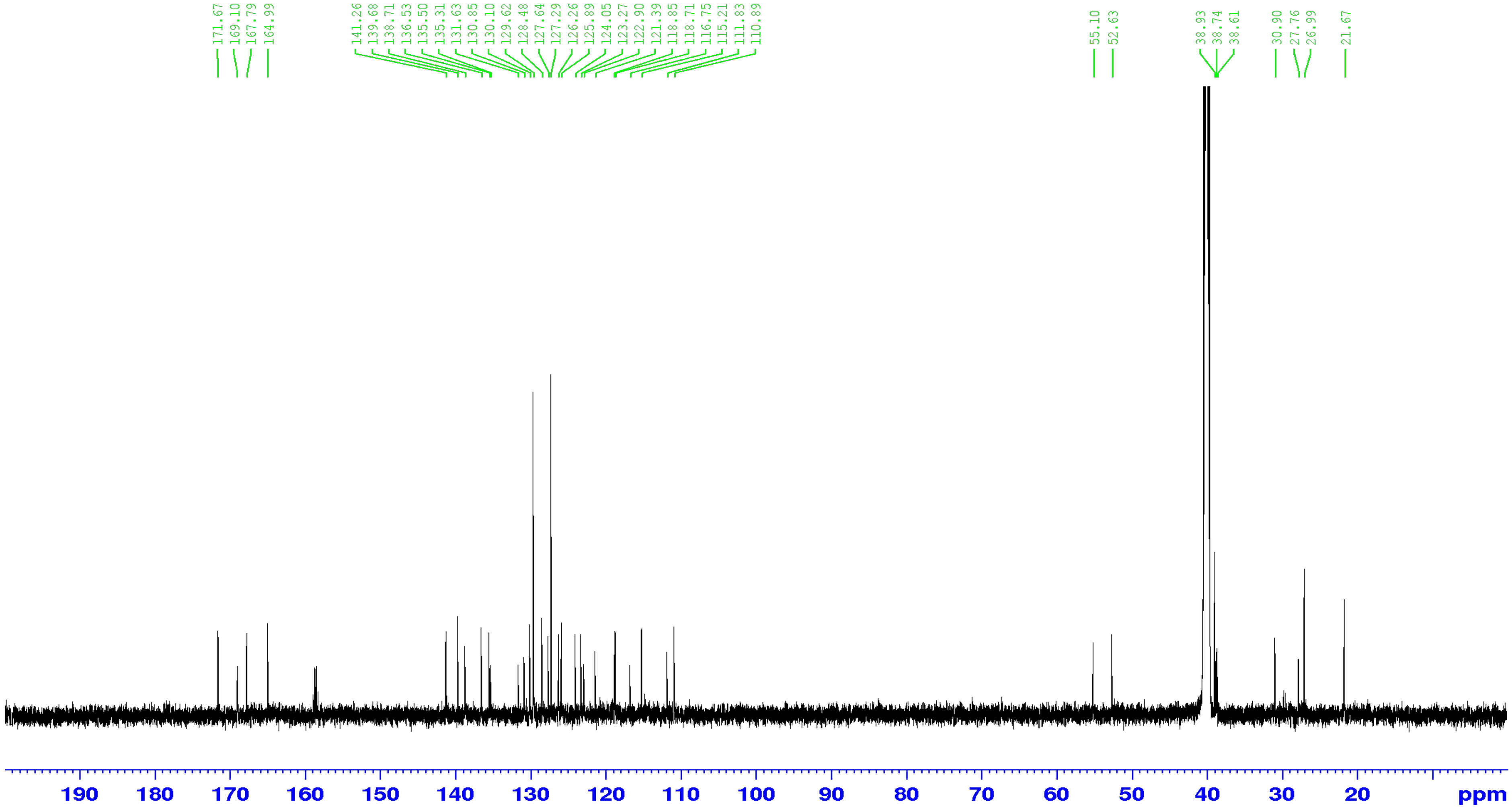

^{1}H NMR of *N*-(4-bromo-2-(((S)-1-((2-((S)-2,6-diaminohexanamido)ethyl)amino)-3-(1*H*-indol-3-yl)-1-oxopropan-2-yl)carbamoyl)phenyl)-[1,1'-biphenyl]-4-carboxamide (19c)

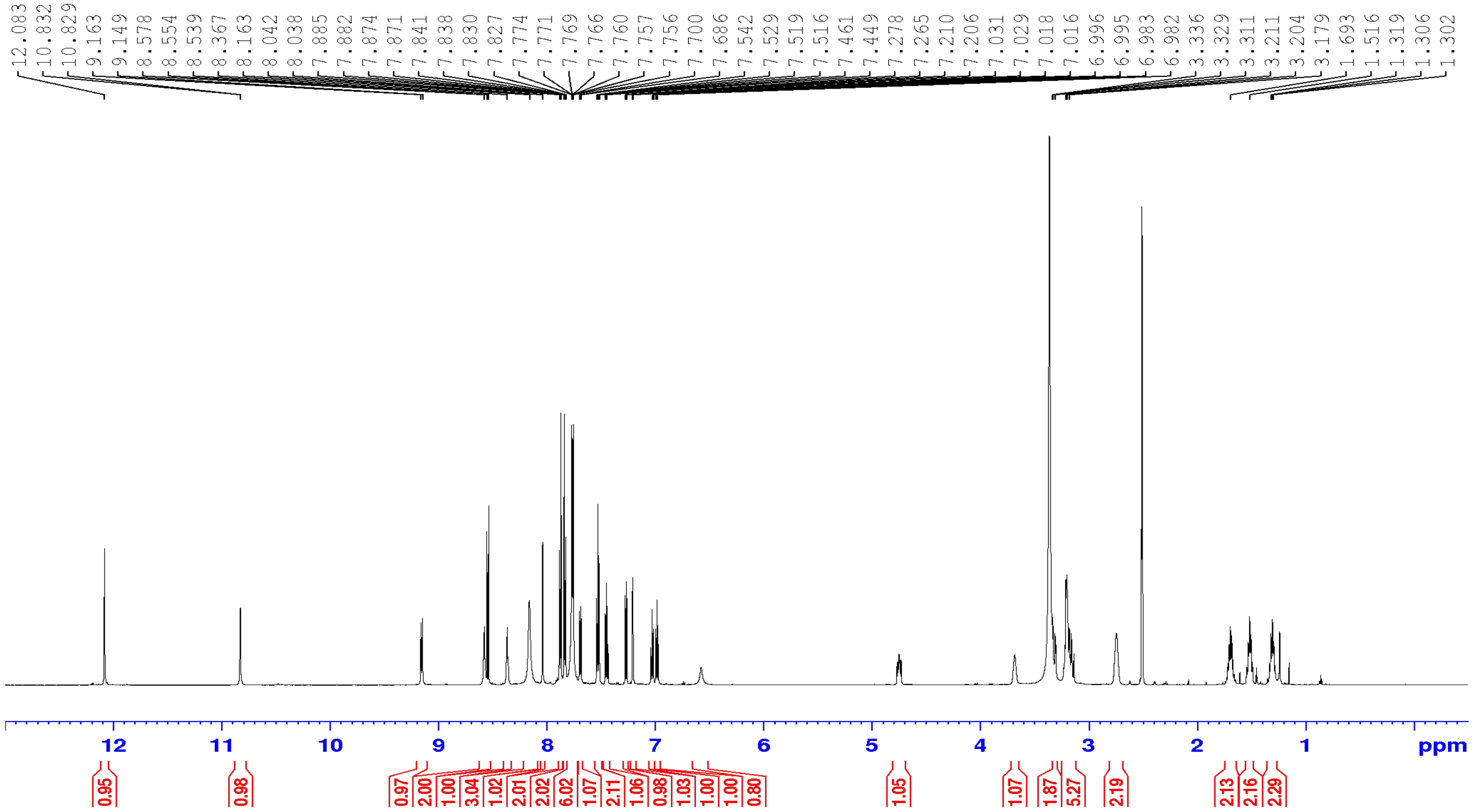

^{13}C NMR of *N*-(4-bromo-2-(((S)-1-((2-((S)-2,6-diaminohexanamido)ethyl)amino)-3-(1*H*-indol-3-yl)-1-oxopropan-2-yl)carbamoyl)phenyl)-[1,1'-biphenyl]-4-carboxamide (19c)

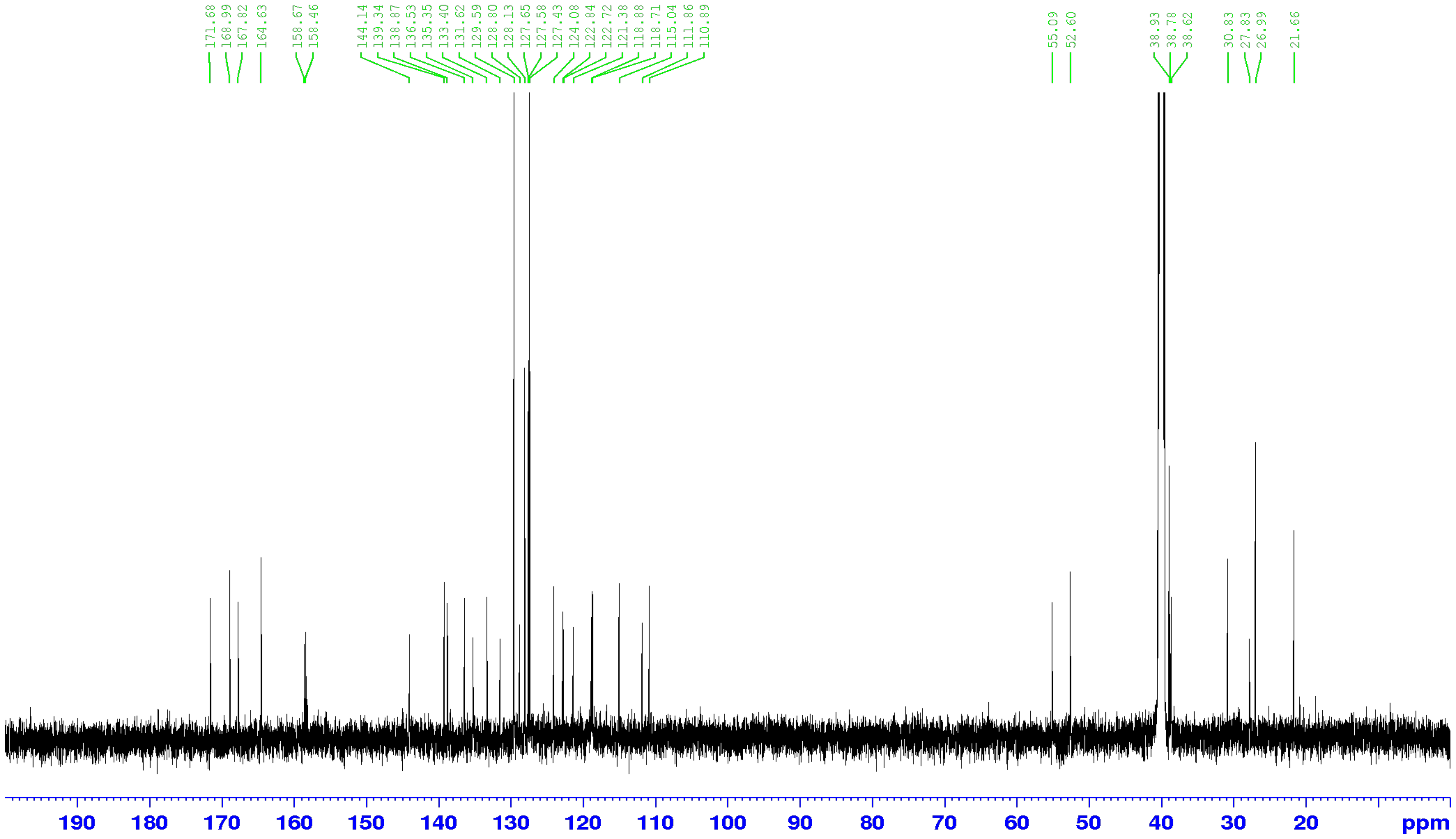

Supplement: Supplementary file 1 [file antibiotics-12-00585-s001.zip › antibiotics-2264288-supplementary.pdf]
